# Construction, De-Novo Assembly and Analysis of Transcriptome for Identification of Reproduction-Related Genes and Pathways from Rohu, *Labeo rohita* (Hamilton)

**DOI:** 10.1371/journal.pone.0132450

**Published:** 2015-07-06

**Authors:** Dinesh Kumar Sahu, Soumya Prasad Panda, Prem Kumar Meher, Paramananda Das, Padmanav Routray, Jitendra Kumar Sundaray, Pallipuram Jayasankar, Samiran Nandi

**Affiliations:** 1 Fish Genetics and Biotechnology Division, ICAR-Central Institute of Freshwater Aquaculture, Kausalyaganga, Bhubaneswar, Orissa, India; 2 Aquaculture Production and Environment Division, ICAR-Central Institute of Freshwater Aquaculture, Kausalyaganga, Bhubaneswar, Orissa, India; Glasgow Caledonian University, UNITED KINGDOM

## Abstract

Rohu is a leading candidate species for freshwater aquaculture in South-East Asia. Unlike common carp the monsoon breeding habit of rohu restricts its seed production beyond season indicating strong genetic control over spawning. Genetic information is limited in this regard. The problem is exacerbated by the lack of genomic-resources. We identified 182 reproduction-related genes previously by Sanger-sequencing which were less to address the issue of seasonal spawning behaviour of this important carp. Therefore, the present work was taken up to generate transcriptome profile by mRNAseq. 16GB, 72bp paired end (PE) data was generated from the pooled-RNA of twelve-tissues from *pre-spawning* rohu using IlluminaGA-II-platform. There were 64.97 million high-quality reads producing 62,283 contigs and 88,612 numbers of transcripts using velvet and oases programs, respectively. Gene ontology annotation identified 940 reproduction-related genes consisting of 184 mainly associated with reproduction, 223 related to hormone-activity and receptor-binding, 178 receptor-activity and 355 embryonic-development related-proteins. The important reproduction-relevant pathways found in KEGG analysis were GnRH-signaling, oocyte-meiosis, steroid-biosynthesis, steroid-hormone biosynthesis, progesterone-mediated oocyte-maturation, retinol-metabolism, neuroactive-ligand-receptor interaction, neurotrophin-signaling and photo-transduction. Twenty nine simple sequence repeat containing sequences were also found out of which 12 repeat loci were polymorphic with mean expected-&-observed heterozygosity of 0.471 and 0.983 respectively. Quantitative RT-PCR analyses of 13-known and 6-unknown transcripts revealed differences in expression level between *preparatory* and *post-spawning* phase. These transcriptomic sequences have significantly increased the genetic-&-genomic resources for reproduction-research in *Labeo rohita*.

## Introduction

The Indian major carp *Labeo rohita* (Hamilton), a cyprinid is a leading candidate species for freshwater aquaculture not only in India but also in the whole sub-continent of South-East Asia with the annual production of 1.5 million tonnes in 2012 [1]. Due to its immense economic, ecological and cultural importance it received lot of research interest in different areas including culture [2], breeding [3] immunology [4], disease ecology [5], reproductive physiology [6] and nutrition [7]. One of the major problem in this species is highly monsoon dependent breeding habit [8] and inability to breed in confined pond water without hormonal induction [9]. This restricts seed production beyond the breeding season leading to suboptimal utilization of cultivable water area. Like other Indian teleost the reproductive cycle of *L*. *rohita* may be divided into four stages, *preparatory* period (February–April), *pre-spawning* period (May–June), *spawning* period (July–August), and *post-spawning* period (September–January) [10] and at each stage gonads show discrete change. Unprecedented summer temperature, scanty, irregular and shifted monsoon in recent years has further complicated seed production by affecting one or more reproductive stages. However, common carp (*Cyprinus carpio*) is from the same cyprinid group, shows prolific breeding in the similar environmental condition. Attempt has been made to study the effects of environmental manipulations on gonadal development [11] through dietary manipulation [12], multiple induced breeding [13], offseason breeding [14] of Indian major carp etc. Although literatures on different aspects of reproduction and breeding are available [3], the genetic mechanism underlying gonad maturation and seasonal breeding in tropical climate has not been fully understood. Genetic studies have been performed in the past decade, which focused on selective breeding [15], development of genetic markers [16], development of linkage map [17] and collection of immune related genes [4]. Reproduction of fish is centrally controlled by brain, pituitary liver and gonad (BPGL axis) [18] and multiple genes are involved in this process. Addressing issues like gonad maturation; spawning and seed production under changing climatic conditions will require comprehensive information on a number of reproduction-related genes. In our earlier attempt 182 reproduction-related genes were identified from 4,642 high-quality ESTs by Sanger sequencing [6], but the number was less. High throughput next generation sequencing technologies provide the platforms to generate transcriptome sequences with much lower cost than traditional Sanger method, thus the transcripts generated by NGS technology can boost genetic and genomic research of relatively lagging species [19]. Until the reference genome sequence becomes available, transcriptome sequencing is a fast and efficient means for gene discovery and genetic marker development. The simple sequence repeat (SSRs) markers are important resources for determining functional genetic variation and among the various molecular markers, SSRs are highly polymorphic [20], and serve as rich resource of diversity. SSRs derived from expressed sequence tags (ESTs) have special advantages such as those might be linked to known genes, having higher transferability among related species, lower cost for development and higher proportion of high-quality markers [20]. Transcriptome resources for reproductive tissues are currently available for other commercially important fishes, including channel catfish (*Ictalurus punctatus*) [21], common carp (*Cyprinus carpio*) [19], zebrafish (*Danio rerio*) [22], rainbow trout (*Oncorhynchus mykiss*) [23,24], coho salmon (*Oncorhynchus kisutch*) [25], tilapia (*Oreochromis mossambicus*) [26], Atlantic halibut (*Hippoglossus hippoglossus*) [27], senegalese sole (*Solea senegalensis*) [28], Atlantic salmon (*Salmo salar*) [29], and cod (*Gadus morhua*) [30] but less information is available for *L rohita* [5,6].

Hence, this work was taken up with the primary objective to generate transcripts sequence using mRNA-seq and provide well-assembled transcriptome sequences from the pooled RNA samples of brain, liver, intestine, kidney, tongue, nose, eye, gill, muscle, heart, ovary and testis tissues to identify reproduction relevant genes, verify their association with reproduction by transcript expression pattern and to develop EST-SSR markers within these transcripts of *L rohita* which may be utilized further in future for genetic diversity analysis, gene mapping, marker-assisted breeding as well as studying reproductive issues in this species.

## Materials and Methods

### Ethics Statement

This study was approved by the Animal Ethics Committee of ICAR-CIFA, Bhubaneswar. All the fishes (rohu, *Labeo rohita*) used in the experiments were handled according to the prescribed guidelines of the Institute.

### Maintenance of Animals and tissue collection

The brood stock of rohu as well as common carp was reared in CIFA farm ponds under carp breeding unit following standard procedures [3,31]. The stocking density was maintained at the rate of 1500kg/ha with 1:1 ratio of rohu and common carp (Lat.20°1'06"–20°11'45"N, Long.80°50'52"–85°51'35"E). Physico-chemical parameters of water were monitored routinely during the entire course of investigation. The water temperature, pH, D.O., total alkalinity and total hardness were 28–30°C, 7.5–8.5, 100–140 ppm, and 100–130 ppm, respectively. Adult males and females of *L rohita* (800–1200g) were collected during May-June, (*pre-spawning*). The fishes were euthanized with MS-222 at 300 mg/L before dissection. Brain, liver, intestine, kidney, tongue, nose, eye, gill, muscle, heart, ovary and testis tissues were collected from minimum five fishes for each tissue, quickly frozen in liquid nitrogen and stored at –80°C, until used for RNA extraction.

### Illumina sequencing and quality controls

Total RNA was extracted from 12 different tissues (50–100mg) following the Guanidium Thiocyanate method [32] using the TRIzol-Reagent (Invitrogen, Carlsbad, CA, USA) according to manufacturer’s instructions. RNA samples were then treated with RNase free DNase I (Qiagen) to remove potential genomic DNA. RNA integrity was initially checked in 1% denaturing gel. The RNA samples showing clear separation of 28S and 18S bands in the gel and spectrophotometric (Varian Cary 50 Bio) A^260/280^ absorption ratio greater than 1.9 were taken for further work. RNA size distribution and integrity were further analyzed in Bioanalyzer 2100 (Agilent, Santa Clara, CA, USA) and samples having RIN (RNA Integrity Number) more than 7.0 were used for library preparation. One paired- end (PE) cDNA library was generated from the pooled total RNA (4μg) of rohu tissues in equal quantity using mRNA-Seq assay for transcriptome sequencing on Illumina Genome Analyzer II platform. The library was constructed according to the Illumina TruSeq RNA library protocol outlined in “TruSeq RNA Sample Preparation Guide” (Part # 15008136). The prepared library was quantified using Qubit (Invitrogen) and validated for quality by running an aliquot on High Sensitivity chip (Agilent, Cat # 5067–4626) on Bioanalyzer 2100 (Agilent technologies, Santa Clara, CA, USA). Sequencing was done in one lane to generate 72bp PE reads. The raw sequences generated by Illumina Genome Analyzer were processed with SeqQC-V2.1 (In-house tool kit of Genotypic Technology) for various quality controls, including filtering of high-quality reads based on the score value, removal of reads containing primer/ adaptor sequences and trimming of read length.

### De novo assembly

All the assemblies were performed on a server with 48 cores processor and 256 GB random access memory. Two programs were used for de novo assembly of the PE sequence reads of rohu. Publicly available program, velvet (version 0.7.62; http://www.ebi.ac.uk/~zerbino/velvet/) was used to generate a non-redundant set of transcripts which has been developed for assembly of short reads using de-Bruijn-graph algorithm. Various assembly parameters were also optimized for best result. The trimmed high-quality sequence reads were assembled using velvet program at different (Hash length) k-mer length such as 31, 33, 35, 37, 39, 41, 43, 45, 47, 49, 51, 53, 55, 57, 59, 61 and 65 with various output parameters like number of used reads, nodes, total number of contigs, contigs longer than 100 bp, N50 length, longest contig length and average contig length as a function of k-mer. Assembly by velvet was followed by oases (version 0.1.8; http://www.ebi.ac.uk/~zerbino/oases/), which has also been developed for de novo assembly of short reads, which takes the assembly generated by velvet as input and exploits the read sequence and pairing information to obtain better contigs/transcripts particularly to get the isoforms.

### Similarity search and functional annotation

Putative function of the velvet assembled contigs was deduced by using them as queries against the UniProtKB/SwissProt database and the non-redundant (nr) protein database in the BLASTX program. The cut off E-value was set at 1e-6 and only the top gene-ID and name were initially assigned to each contig. Gene ontology (GO) annotation analysis was performed in Blast2GO (http://www.blast2go.org/) version 2.5.0 for the assignment of gene ontology terms. The nr BLAST result was imported to Blast2GO. The final annotation file was produced after gene-ID mapping, GO term assignment, annotation augmentation and generic GO-slim process. The annotation result was categorized with respect to Biological Process, Molecular Function, and Cellular Component at level 2. Pathway analyses of unique sequences were carried out based on the Kyoto Encyclopedia of Genes and Genomes (KEGG) database using the online (http://www.genome.jp/tools/kaas/) KEGG Automatic Annotation Server (KAAS) by using Bi-directional Best Hit (BBH) method. Enzyme commission (EC) numbers were obtained and used to putatively map protein sequences to a specific biochemical pathway.

### Mapping and assessment of sequence reads onto rohu transcripts

To assess transcript and exon distribution in the genome, all the transcripts of oases assembly were mapped to the complete genome of zebrafish (zv9) and all the transcripts with significant hits were plotted by zebrafish chromosome number. For this, putative rohu non redundant transcripts were aligned with the reference sequences of zebrafish genome (zv9) using top hat alignment programme, compared and mapped in digital gene expression file using cufflinks program. In addition, the coverage of each transcript was determined in terms of number of fragment per kilobase of exon per million fragment mapped (FPKM). Further to assess transcript distribution in the genome of other species, the non redundant transcript data set of oases assembly was further subjected to BLAST against the mRNA databases of zebrafish (*Danio rerio*), salmon (*Salmo salar*) and catfish (*Ictalurus punctatus*) separately in NCBI. Those transcripts having more than 70% identity and 50% query coverage (mRNA) were taken for further analysed to get the mRNA annotated transcripts, mRNA overlapping transcripts and list of mRNA matching in that particular species.

### Identification of orthologous genes involved in reproduction

To identify the genes and transcripts that play important role in reproduction process, the transcripts associated with GO terms under reproduction (GO: 0000003), hormone activity (GO: 0005179), receptor binding (GO: 0005102), receptor activity (GO: 0004872) and embryonic development (GO: 0009792) were selected.

### Identification of EST-SSRs, SNPs and validation of microsatellite containing transcripts

To identify all the simple sequence repeats in assembled transcriptome of *L rohita*, perl script programme MISA (http://pgrc.ipk-gatersleben.de/misa/) was used. The mono-nucleotide repeats (more than 10 times), di-nucleotide (more than 6 times), tri, tetra, penta- nucleotide (more than 5 times) were considered as search criteria in MISA script. Maximum number of bases interrupting between two SSRs in a compound microsatellite was taken as 100. For the analysis of microsatellite polymorphism, DNA from 3 parents belonging to two linkage mapping panels of rohu [33] and 2 individuals (one each) from resistant and susceptible lines of rohu against aeromoniasis [5] was used to test the gene loci for diversity. Number of alleles, observed heterozygosity (HO), expected heterozygosity (HE) and polymorphic information content (PIC) were estimated using CERVUS software ver 3.0 (http://www.fieldgenetics.com/pages/home.jsp). The difference between observed and expected heterozygosity was tested using chi square test by SAS 9.2 version for significant deviation. Further, to study the presence of SSRs in other species, corresponding gene sequences from zebrafish as well as common carp were downloaded from the databases and SSRs were searched in these sequences as described for rohu. To identify SNPs in rohu contigs, reads were mapped with the complete genome of zebrafish (zv9) using Bowtie-0.12.7 and variations were detected by Sam tools 0.1.7 with maximum variant count ≥ 10.

### Putative ORF sequences searching

Unidentified sequences (no match found in BLASTX and BLASTN) among unique sequences were analyzed by star-orf software (http://web.mit.edu/star/orf/runapp.html) using parameter of 80bp minimal ORF length to search for putative ORF proteins, which could be used to distinguish between coding and non-coding sequences. Once the start codon, coding sequences, stop codon and poly (A) tail were identified, the cDNA sequences were considered a full-length cDNA, all the possible frames were searched against the protein database using BLASTP tool of NCBI.

### Expression analysis of selected orthologous gene using quantitative real-time PCR during *preparatory* and *post-spawning* phases

For the quantitative real time study, common carp and rohu were collected from the same earthen pond having same aquatic environment, as mentioned previously in animal maintenance section. Relative expression level of 19 transcripts (13 known and 6 unknown genes with putative ORFs) were measured by real-time PCR in brain, liver, pituitary, ovary and testis tissues collected from 15 individuals during initiation of gonad maturation (preparatory) and resting phase (post-spawning) each, using β-actin as a reference gene (GenBank accession no. EU184877) from rohu. Similarly, brain, liver, pituitary and ovary from common carp (Cyprinus carpio) was also collected from 15 individuals during preparatory phase for comparison with rohu. Equal quantity of tissue from each individual was pooled into three sets (5 fishes in each set) and RNA was extracted for each pooled tissues following the Guanidium Thiocyanate method [32] using the TRIzol-Reagent (Invitrogen, Carlsbad, CA, USA) according to manufacturer’s instructions. First strand cDNA was synthesized using M-MLV reverse transcriptase (Finnzymes, Vantaa, Finland). Validation of transcript specific primers (S1 Table) was checked by normal PCR and band intensities for different tissues were observed in agarose gel electrophoresis with β-actin as control (data not shown). The real time PCR amplifications were carried out using a Light Cycler 480 (Roche, Germany) with a negative control with no template. Real time PCR was repeated twice for each tissue for each sample. The crossing point, Cp values were acquired for both the target and reference gene using software version LCS480 1.5.0.39 of Light Cycler 480 (Roche, Germany). The relative transcript level of each transcript in each tissue was calculated by normalization of the value with the corresponding reference and compared among them using Cp values for brain cDNA as positive calibrator [34]. Comparison of relative expression level of each transcript between the two reproductive phases in individual tissue as well as between the two species was analyzed in REST 2009 software and the whisker-box plots were extracted with 2000 time iterations (http://www.REST.de.com).

## Results

### De novo assembly

**Fig 1:**
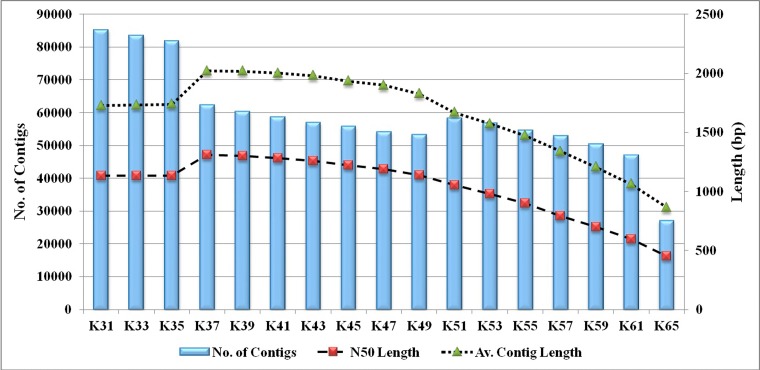
Comparison of number of contigs, N50 length, average contigs length, at different k-mer value.

A total of 74,725,656 PE (37,362,828 from each end) raw sequence reads with each 72 bp length were generated using Illumina Genome Analyzer II, encompassing about 16 GB of sequence data in fastq format. The raw reads produced have been deposited in the NCBI SRA database (accession number: SRA051586). After filtering the sequence data for low-quality reads at higher stringency and reads containing primer/adaptor sequence, resulted in a total of 64,971,614 (87%) high-quality sequence reads (more than 70% of bases in a read with more than 20 phred score). The final data set comprising 64.97 million high-quality reads was used for optimization of de novo assembly and analysis of rohu transcriptome (Table 1). N50 length of the contigs generated using velvet assembly program varied from 454 to 1309 of different k mer (31–65) values (Fig 1). Out of these the best k mer value found was 37, as it resulted in highest N50 length of 1309 bp, largest contig length of 16,961 bp and largest average contig length of 709 bp. The assembly resulted in a total of 62,283 contigs containing 8 contigs of 10 Kb, 13,707 contigs of 1 Kb, 26,145 contigs of 500 bp with a minimum of 100 bp lengths (Table 2). The total number of reads used for the assembly was also highest (69.61%) for k mer value of 37. The assembly of contigs generated by velvet at k-mer 37 was used again as input data in oases with default parameters. This resulted in a total number of 88,612 transcripts in comparison to the 62,283 contigs resulted from velvet.

**Table 1:** QC report for fastQ files.

	TruSeq Library 5' Adapter	TruSeq Library 3' Adapter
Fastq file size	8.08 GB	8.08 GB
Maximum Read Length	72	72
Total Number of Reads	37362828 (37.36 millions)	37362828 (37.36 millions)
Total Number of HQ Reads after removing primer/ adapter containing reads1*	33661189 (33.66 millions)	31310425 (31.31 millions)

^1^* >70% of bases in a read with >20 phred score

**Table 2:** Statistics of transcriptome assembly.

Contigs Generated:	62,283
Maximum Contig Length:	16,961
Minimum Contig Length:	100
Average Contig Length	709.968
Contigs > 100 bp:	62,030
Contigs > 500 bp:	26,145
Contigs > 1 Kb:	13,707
Contigs > 10 Kb:	8
n50_Perl	1,309
Percentage of reads assembled	69.61%

### Functional annotation and identification of reproduction-related genes

**Fig 2:**
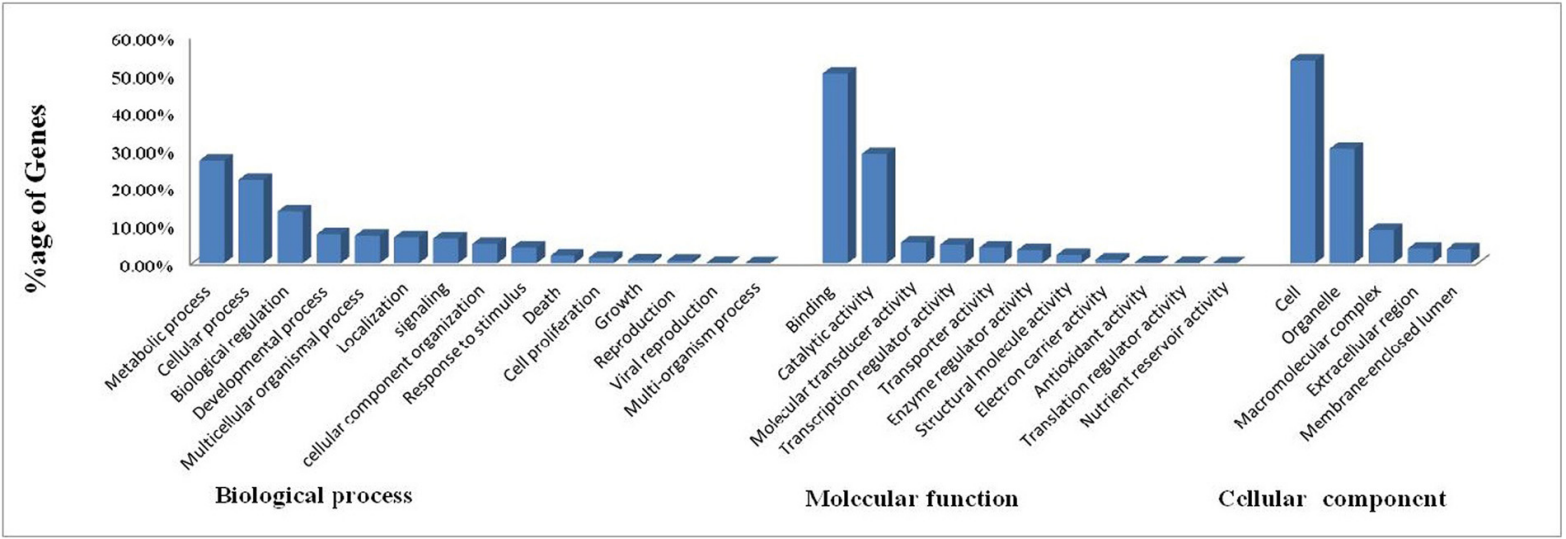
Percentages of annotated *Labeo rohita* sequences assigned with GO terms according to level 2 categories. GO-terms were processed by Blast2Go and categorized at level 2 under three main categories. Each of the three GO categories is presented including (left to right): biological process, molecular function and cellular component.

**Fig 3:**
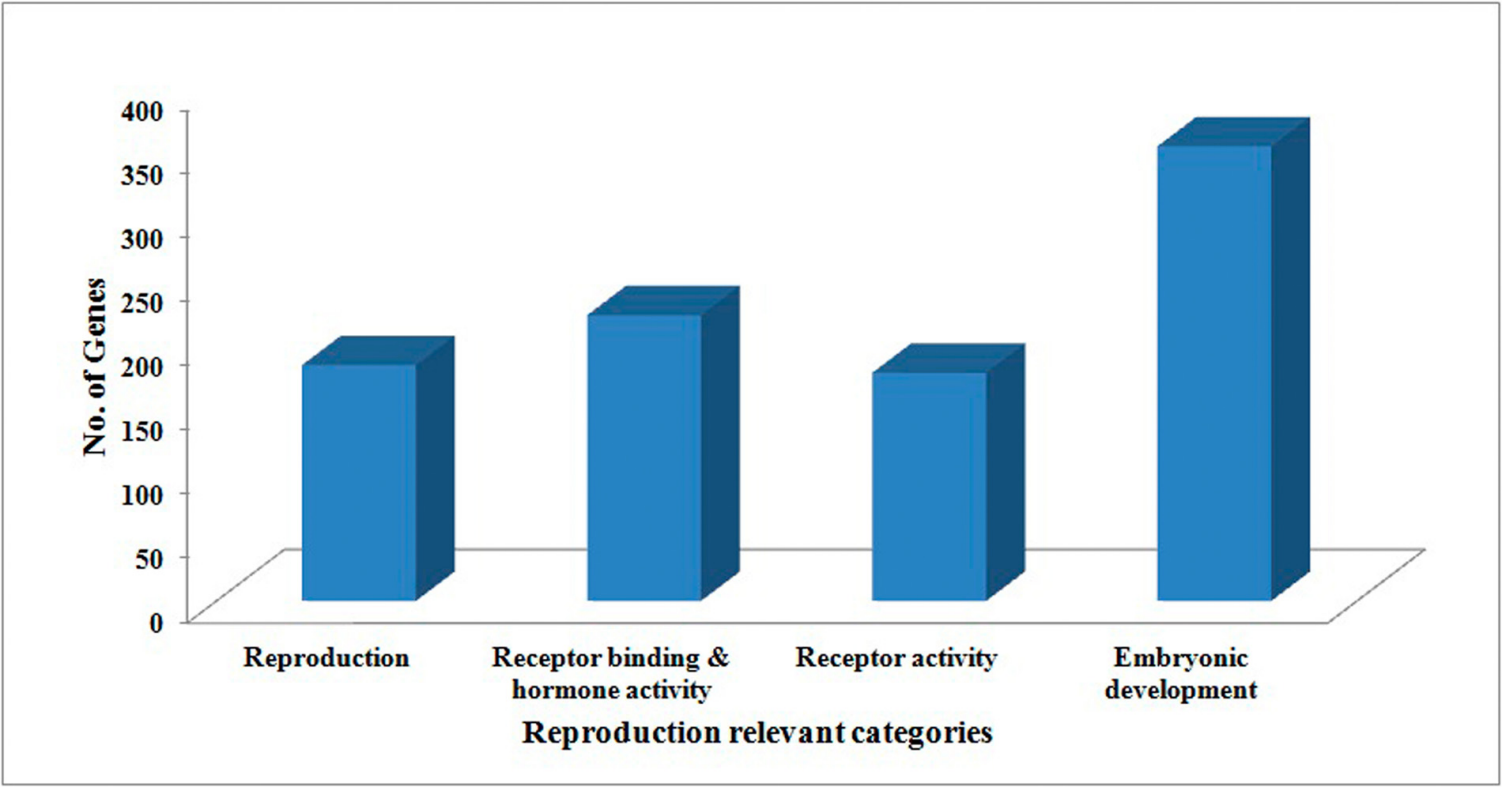
Distribution of reproduction-relevant transcripts identified in *Labeo rohita*. A total of 940 reproduction relevant genes were distributed among 184 reproduction related proteins (GO: 0000003), 223 related to hormone activity (GO: 0005179) and receptor binding related proteins (GO: 0005102), 178 receptor activity related proteins (GO: 0004872) and 355 embryonic development related proteins (GO: 0009792).

A total of 31,637 contigs had significant BLASTX hit corresponding to 17,925 unique protein accessions in the nr protein database. Gene ontology (GO) analysis of these 17,925 unique proteins resulted in a total of 78,317 annotations/GO terms including 33,343 (42.57%) biological process terms, 23,479 (29.98%) molecular function terms and 21,495 (27.44%) cellular component terms (Fig 2). Among the biological process category 7,841 and 7,375 genes were related to metabolic (GO: 0008152) and cellular processes (GO: 0009987) respectively; a significant numbers of genes were also identified from development process (2,534) and growth (248) sub-categories. Similarly under molecular function category, 11,798 genes were involved in the binding process (GO: 0005488) and 6,784 genes in the catalytic activity (GO: 0003824); whereas under the cellular component category, 11,533 genes from cell (GO: 0005623), and 6,500 genes corresponded to organelle (GO: 0043226) were the most represented categories. A total of 940 reproduction relevant genes were identified (Fig 3), among which 184 were mainly associated with reproduction related proteins (GO: 0000003), 223 related to hormone activity (GO: 0005179) and receptor binding related proteins (GO: 0005102), 178 receptor activity related proteins (GO: 0004872) and 355 embryonic development related proteins (GO: 0009792). Details of 940 reproduction related gene orthologues are given in S2 Table.

### Mapping of rohu transcripts with zebra fish, salmon, and catfish

**Fig 4:**
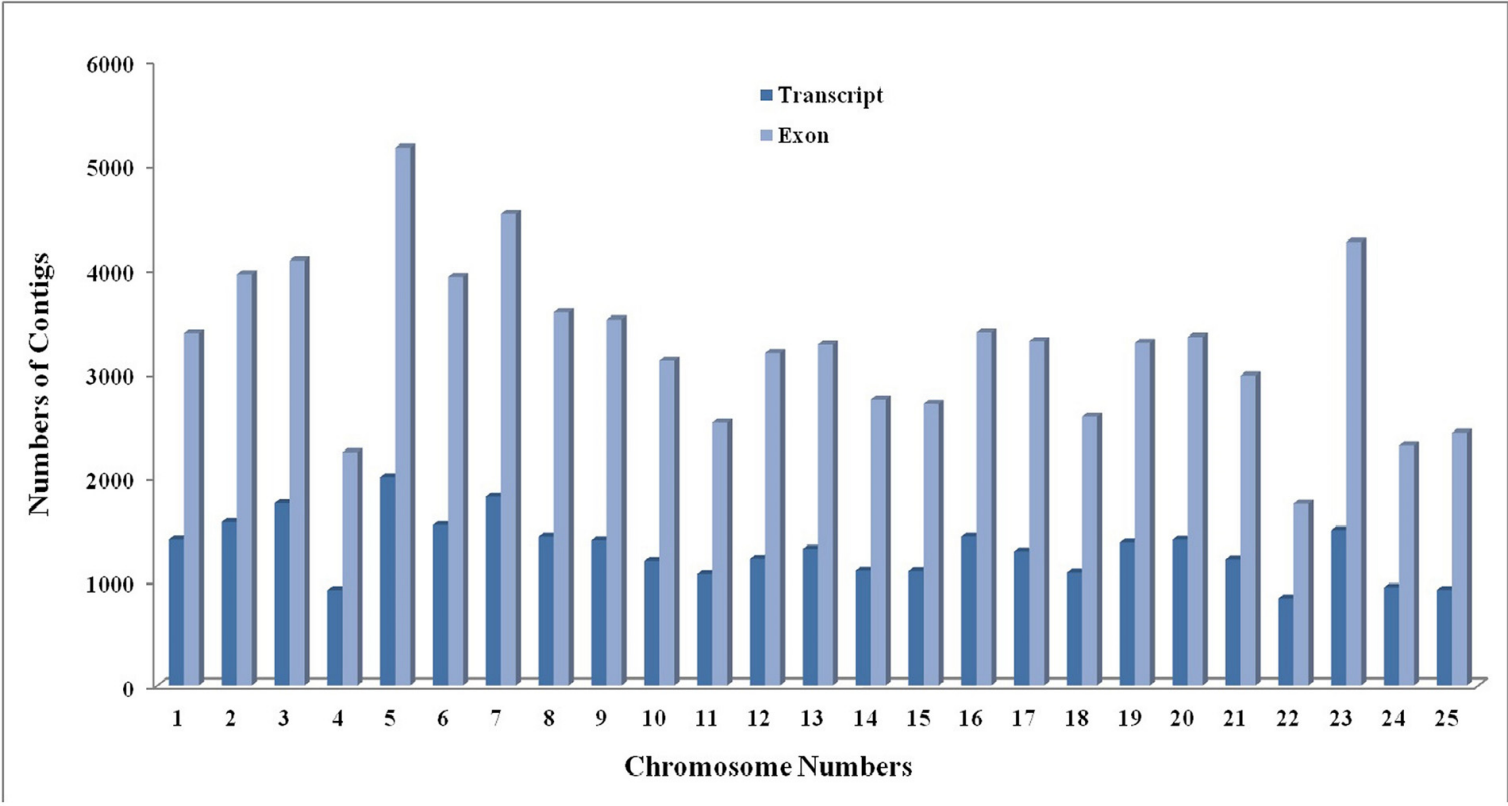
Distribution of rohu transcripts and exons on zebra fish chromosome. rohu transcripts found distributed in all 25 chromosomes of zebrafish.

Among 31,091 rohu transcripts, 81,622 exons were found distributed, and all exons were distributed in 25 chromosomes of zebrafish. Out of 25 chromosomes, chromosome number 5 and 23 showed maximum number of match with rohu transcripts (1998 and 1487) and exons (5166 and 4267) respectively (Fig 4). On the other hand, out of 88,612 oases based rohu transcripts when compared with zebra fish, salmon, and catfish mRNA showed match with 31,091, 4,894 and 1,071 numbers of annotated transcripts respectively (Table 3). Interestingly it also showed 794 transcripts common in all these species (S1 Fig).

**Table 3:** The oases contigs/transcripts blasted against the Salmon, Zebrafish, and Catfish mRNA sequences (from NCBI). Those contigs with more than 70% identity and 50% query coverage (mRNA) as cut offs was taken for analysis;

Contig annotation summary	Salmon mRNA	Zebrafish mRNA	Catfish mRNA
**Total contigs**	88,612	88,612	88,612
**Total Annotated contigs**	4,894	31,091	1,071
**Total Un-annotated contigs**	83,718	57,521	87,541

### KEGG-pathway analysis for identification of reproduction related pathways

**Fig 5:**
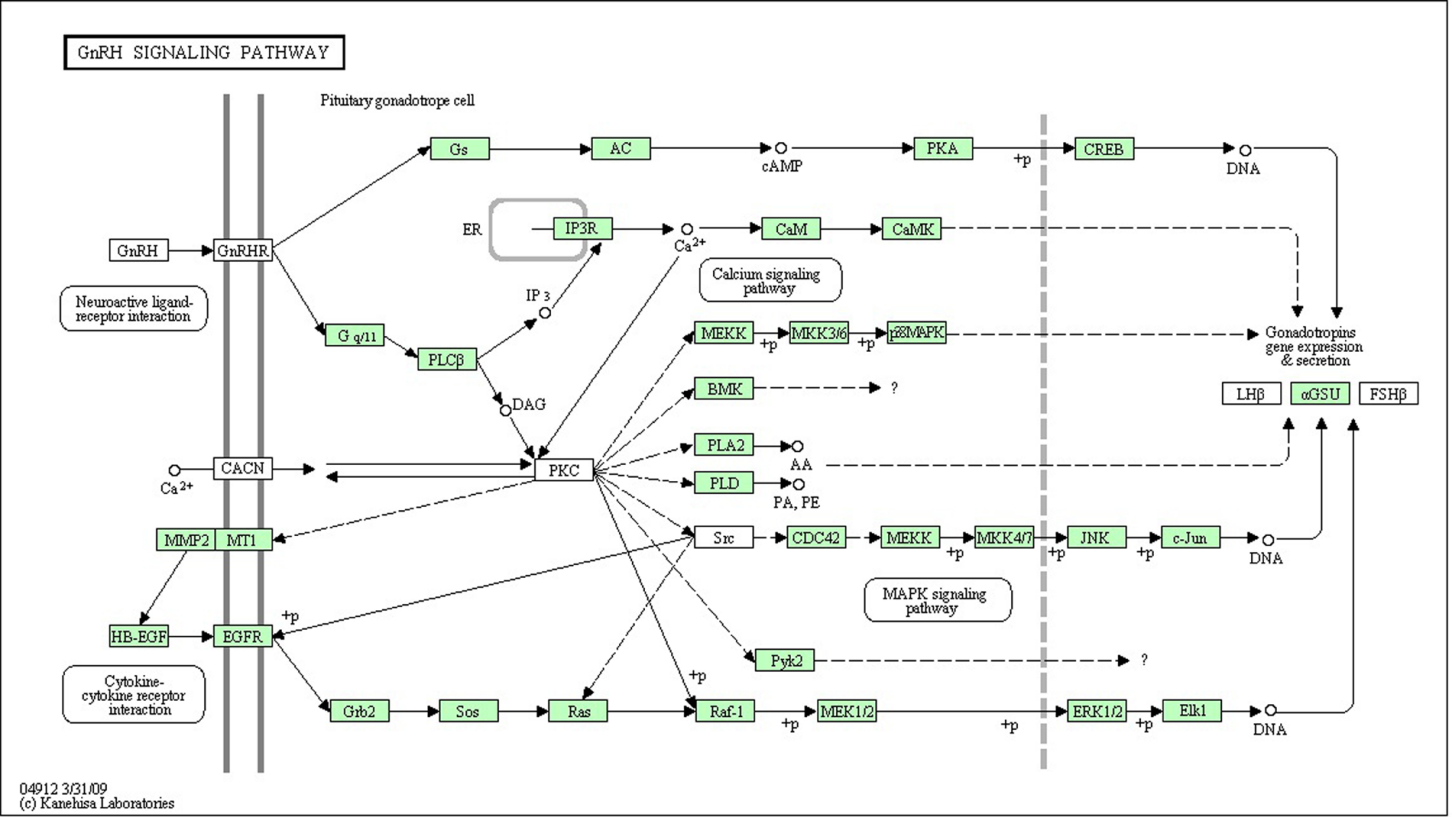
GnRH Pathway in rohu: 66 transcripts coding for 40 genes were mapped in rohu, out of 121 genes of GnRH pathway (KEGG databases).

KEGG (Kyoto Encyclopedia of Genes and Genomes) pathway analysis was performed on all assembled contigs as alternative approach for functional categorization and annotation. Several KEGG pathways were represented by more than 200 unique transcripts. Enzyme commission (EC) numbers were assigned with 5,938 enzyme codes for 8,683 unique sequences (Table 4). Briefly, 2,269 (26.13%) were classified into the metabolism, 1,365 (15.72%) sequences grouped into the Genetic information processing (GIP), 1,188 (13.68%) unique sequences come under Environmental information processing (EIP), 1,283 (14.77%) unique sequences under cellular processes and finally 2,578 (29.69%) sequences under organismal systems had match with KEGG annotation (Table 4). Among reproduction-relevant pathways, GnRH signalling pathway (Fig 5) (66 unique transcript sequences coding for 40 genes of rohu out of 121 genes), oocyte meiosis (out of 137 genes 86 unique transcript sequences coding for 53 genes), steroid biosynthesis (16 unique transcript sequences coding for 14 enzymes of rohu out of 28 genes), steroid hormone biosynthesis (18 unique transcript sequences coding for 15 enzymes of rohu out of 38 genes), progesterone-mediated oocyte maturation (72 unique transcript sequences coding for 46 genes of rohu out of 107 genes), retinol metabolism (21 unique transcript sequences coding for 17 enzymes of rohu out of 38 genes), neuroactive ligand-receptor interaction (70 unique transcript sequences coding for 63 genes), neurotrophin signaling pathway (98 unique transcript sequences coding for 67 genes) and phototransduction (19 unique transcript sequences coding for 13 enzymes) were the major pathways found.

**Table 4:** KEGG mappings for *Labeo rohita*.

KEGG categories represented	Number of KO	Unique sequences
**Metabolism**	**1,511**	**2,269**
Carbohydrate Metabolism	284	449
Energy Metabolism	150	195
Lipid Metabolism	246	377
Amino Acid Metabolism	265	362
Nucleotide Metabolism	178	247
Metabolism of Cofactors and Vitamins	111	140
Glycan Biosynthesis and Metabolism	182	222
Metabolism of Other Amino Acids	67	95
Xenobiotics Biodegradation and Metabolism	76	118
Biosynthesis of Secondary Metabolites	24	31
Metabolism of Terpenoids and Polyketides	28	33
**Genetic Information Processing**	**1,063**	**1,365**
Transcription	173	207
Translation	335	462
Folding, Sorting and Degradation	360	464
Replication and Repair	195	232
**Environmental Information Processing**	**808**	**1,188**
Membrane Transport	33	36
Signal Transduction	508	834
Cell Growth and Death	54	61
Signaling Molecules and Interaction	213	257
**Cellular Processes**	**888**	**1,283**
Transport and Catabolism	327	450
Cell Motility	99	154
Cell Growth and Death	233	316
Cell Communication	229	363
**Organismal Systems**	**1,668**	**2,578**
Immune System	543	789
Endocrine System	271	422
Circulatory System	67	112
Digestive System	264	418
Excretory System	77	130
Nervous System	281	456
Sensory System	34	60
Development	118	170
Environmental Adaptation	13	21
**Total**	**5,938**	**8,683**

### Identification of EST-SSRs and SNPs

A total of 22,383 EST-SSRs were identified in 17,244 transcripts of rohu with a frequency of one SSR per 3.41 kb of the sequence (Table 5). The mono-nucleotide repeats represented the largest fraction (60.33%) of SSRs identified followed by di-nucleotide (19.21%) and tri-nucleotide (9.83%) repeats. Only a small fraction of tetra- (390), penta- (70) and hexa-nucleotide (23) repeats were identified in rohu transcripts (Table 5 and S2 Fig). In total, there were 1893 compound repeats. Screening of microsatellites from reproduction relevant known genes revealed 29 different microsatellite containing sequences with different repeat motifs (Table 6). From 29 reproduction relevant microsatellite containing sequences 51 primers were designed from the flanking region, out of which 38 primers were selected for testing, but 32 (84%) primers showed PCR amplification. Checking further with these 32 loci in the mapping panel parents, twenty loci were found to be monomorphic and twelve polymorphic. The genetic diversity measures using CERVUS indicated mean expected and observed heterozygosity to be 0.471 and 0.983, respectively. Mean polymorphism information content was found to be 0.35 (Table 7). The observed heterozygosity was lower for the gene loci, SP02, SP04 and SP12, while it was higher in gene loci SP08, SP09, SP14, SP15, SP20 and SP21 than the expected. The difference between expected and observed heterozygosity was not significant (P>0.05). No linkage was detected among the loci. The difference between mean expected and mean observed heterozygosity was not significant (P>0.05). Observed and expected heterozygosity differed at individual locus level (Table 7). Corresponding to 29 microsatellite containing sequences in rohu 26 gene sequences were found for zebrafish and the matching percentage was greater (74%- 98%) at nucleotide level, as compared to only 3 sequences found for common carp with lower (10%- 91%) identity. Microsatellites could be observed in six zebrafish sequences but not in the common carp sequences (S3 Table). A total of 52925 SNPs were identified in 19402 transcripts of rohu (Table 8), out of which 1827 were homozygous and 51098 heterozygous (S4 Table).

**Table 5:** Statistics of microsatellite search in *Labeo rohita*.

Unit size of microsatellite	Number of repeats
Total number of sequences examined:	88,612
Total size of examined sequences (bp):	7,6487,670
Total number of identified SSRs:	22,383
Number of SSR containing sequences:	17,244
Number of sequences containing more than 1 SSR:	3,827
Number of SSRs present in compound formation:	1,893
Number of SSRs with mono-nucleotide:	13,505
Number of SSRs with di-nucleotide:	4,301
Number of SSRs with tri-nucleotide:	2,201
Number of SSRs with tetra-nucleotide:	390
Number of SSRs with penta-nucleotide:	70
Number of SSRs with hexa-nucleotide:	23

**Table 6:** List of microsatellites containing reproduction-relevant transcripts identified in *Labeo Rohita*.

Sl. No	Gene Identity	Repeats Types	Length (bp)	Similarity with organism	Expect	Locus
**Embryonic development related Proteins**						
1	Bromodomain, SNF2L2-like subfamily, specific to animals SNF2L2 (SNF2-alpha)	(AAG)_5_	4966	*Danio rerio*	2.00e-55	SP01
2	Myst3 Protein	(TCA)_5_(TCC)_4_	5344	*Danio rerio*	0.0	SP02, SP03
3	E3 ubiquitin-protein liqase TRIM33	(TGC)_5_(CCG)_4_	3239	*Danio rerio*	0.0	SP04, SP05
4	Glycylpeptide N-tetradecanoyltransferase 1	(AAG)_4_	3359	*Danio rerio*	0.0	SP06
5	N-terminal Src homology 2 (SH2) domain found in Spt6; N-terminal SH2 domain in Spt6	(ATC)_4_	5982	*Danio rerio*	1.16e-31	SP07
6	Transcription factor Sp3 isoform X4	(AT)_7_(GTGC)_3_	1408	*Danio rerio*	2e-100	SP08
7	Protein SMG5	(GA)_6_(GAT)_4_	2199	*Danio rerio*	0.0	SP09, SP10
8	Mitogen-activated protein kinase 3	(AC)_7_	2102	*Astyanax mexicanus*	0.0	SP11
9	Hepatic Leukemia factor	(TGG)_4_	1093	*Danio rerio*	4e-76	SP12
10	Histone acetyltransferase p300 isoform X2	(GCA)_4_(CAG)_4_(GCA)_7_(GCA)_4_(CAG)_7_(GCA)_5_(CAA)_4_(CAG)_5_(TAT)_7_	4583	*Danio rerio*	0.0	SP13, SP14, SP15, SP16, SP17
11	smg5 homolog nonsense mediated mRNA decay factorprotein	(CAA)_4_(CCTCT)_3_	4208	*Danio rerio*	0.0	SP18
12	Tubby-like protein 3	(GT)_10_	1240	*Danio rerio*	6.6E-63	SP19
13	Chromodomain-helicase-DNA-binding protein 7	(TCA)_4_	1688	*Danio rerio*	1.3E-134	SP20
14	Heavy chain non-muscle	(AAAAT)_3_	5265	*Danio rerio*	0.0	SP36
15	Mediator of RNA polymerase ii transcription subunit 12	(CTG)_5_(CTG)_4_(CTG)_4_(CTG)_4_(TTG)_5_(TGC)_4_	858	*Danio rerio*	4.8E-70	SP21, SP22
16	Leucine-zipper protein	(TTCT)_3_(AACAA)_3_	3284	*Danio rerio*	6.4E-92	SP23
**Hormone and Receptor binding related proteins**						
**17**	Docking protein isoform cra_a	(CCA)_7_	2052	*Danio rerio*	1.4E-179	SP24
**18**	Hepatoma-derived growth related protein 3	(TG)_7_	1323	*Danio rerio*	4.8E-93	SP25
19	Ephrin b2	(TCA)_6_	1234	*Danio rerio*	0.0	SP26
20	Neuregulin 1 type i isoform	(TTAT)_4_	2204	*Danio rerio*	3.0E-25	SP27
**Receptor activity related proteins**						
21	T-cell receptor beta chain ana	(AC)_7_	257	*Brugia malayi*	4.1E-17	SP28
22	Protein tyrosine non-receptor type 2	(GCA)_4_	1608	*Danio rerio*	0.0	SP29
23	Gravin	(TC)_6_	6690	*Danio rerio*	0.0	SP30
24	af177465_1 estrogen receptor beta2	(TC)_6_	2803	*Cyprinus carpio*	0.0	SP31
25	Growth factor receptor-bound protein 10	(AC)_6_(TAA)_4_	2667	*Danio rerio*	0.0	SP32, SP33
**Reproduction related proteins**						
26	b-cell leukemia lymphoma 6	(ATA)_5_	1825	*Danio rerio*	0.0	SP34
27	Diaphanous homolog 2	(AACA)_3_	2184	*Oreochromis niloticus*	0.0	SP37
28	HIV-1 rev binding protein	(CAG)4(GCA)_6_	1095	*Danio rerio*	4.5E-122	SP35,
29	TATA box binding protein	(TGCTGT)_3_	1155	*Danio rerio*	2.0E-141	SP38

**Table 7:** Microsatellite Locus, repeat type motif and PCR product size, amplification temp, Number of alleles, observed heterozygosity (HO), expected Heterozygosity (HE) and polymorphic in content (PIC) of 12 rohu microsatellite loci.

Sl.No.	Locus Name	Primer Pair Sequence (5’-3’)	Product Size(bp)	Motif	Tm	Number of alleles	Observed heterozygosity (Hobs)	Expected Heterozygosity (HExp)	Polymorphic in content (PIC)
1	SP02	F TCTTTCATCTTCCTCCTCCTCA	296	{TCA}_5_	60.32	2	0.200	0.556***	0.375
		R ATGAGAAAAAGAGCTTCCCAAC							
2	SP04	F GTTATCTGTGGTGGACCTCCTT	282	{TGC}_5_	60.19	3	0.400	0.622**	0.499
		R AAAAGTGCCACATACTGCTCCT							
3	SP07	F TCTCTTGGGTGAAGTGTGTCC	300	{ATC}_4_	60.62	2	0.250	0.250	0.195
		R GGAAGAGGAAGAACCTCAAAGTG							
4	SP08	F CTCCAGTTGGTCACTGTGTCTG	290	{AT}_7_	60.8	2	0.800	0.533**	0.365
		R TTTTGTGTATGAGCGCAAGTTC							
5	SP09	F CAGAGCAGTAAAGTGGGAGAGA	297	{GA}_5_	59.75	2	1.000	0.556^NE^	0.375
		R TCTCAGAGCAGTGGAGATGAAG							
6	SP11	F CGCGATAGAAAACAAAAAGTCC	294	{AC}_7_	60.13	2	0.200	0.200	0.164
		R TTTCATTCCACAGAGATGGAGA							
7	SP12	F CATTTCCAGACAAACGATCCA	300	{TGG}_4_	60.88	3	0.200	0.378*	0.314
		R CCTCCCACTCCATACAAACC							
8	SP14	F TAAATCCTGGGACAACTCAAGG	244	{CAG}_4_	60.76	2	0.600	0.467**	0.332
		R CGCCTCATCAAATCTCTGAACT							
9	SP15	F CAATGAATCCACTTCAGCAACA	261	{GCA}_4_	61.06	2	1.000	0.556^NE^	0.375
		R GCTGGAACTGTGTATGGTTTTC							
10	SP16	F AGGGACCCAGCAAAACTATTC	288	{GCA}_5_	59.47	2	0.200	0.200	0.164
		R GATCCTCCTCCTAAGTGTGACTG							
11	SP20	F ATGCAGGTAAGAGAAGCCTTTG	294	{TCA}_4_	60.07	3	1	0.644^NE^	0.492
		R GAGGAAGAGGAAGATGATGGTG							
12	SP21	F CTGGAGCTGTCTGACGAGTG	297	{CTG}_5_	61.83	3	1	0.689^NE^	0.548
		R CAGCAGCAACAACAACAACAAG							

***—Highly significant (p≤0.001)

** significant difference (p≤0.05)

* different (p≤0.1)

^NE^- Not Estimated

**Table 8:** Identification of SNPs from rohu contigs.

Total contigs	88,612
Number of contigs having variations	19,402
Total SNPs	52,925
Homozygous SNPs	1,827
Heterozygous SNPs	51,098

### Expression analysis of reproduction-relevant transcripts during *preparatory* and *post-spawning* phases

**Fig 6:**
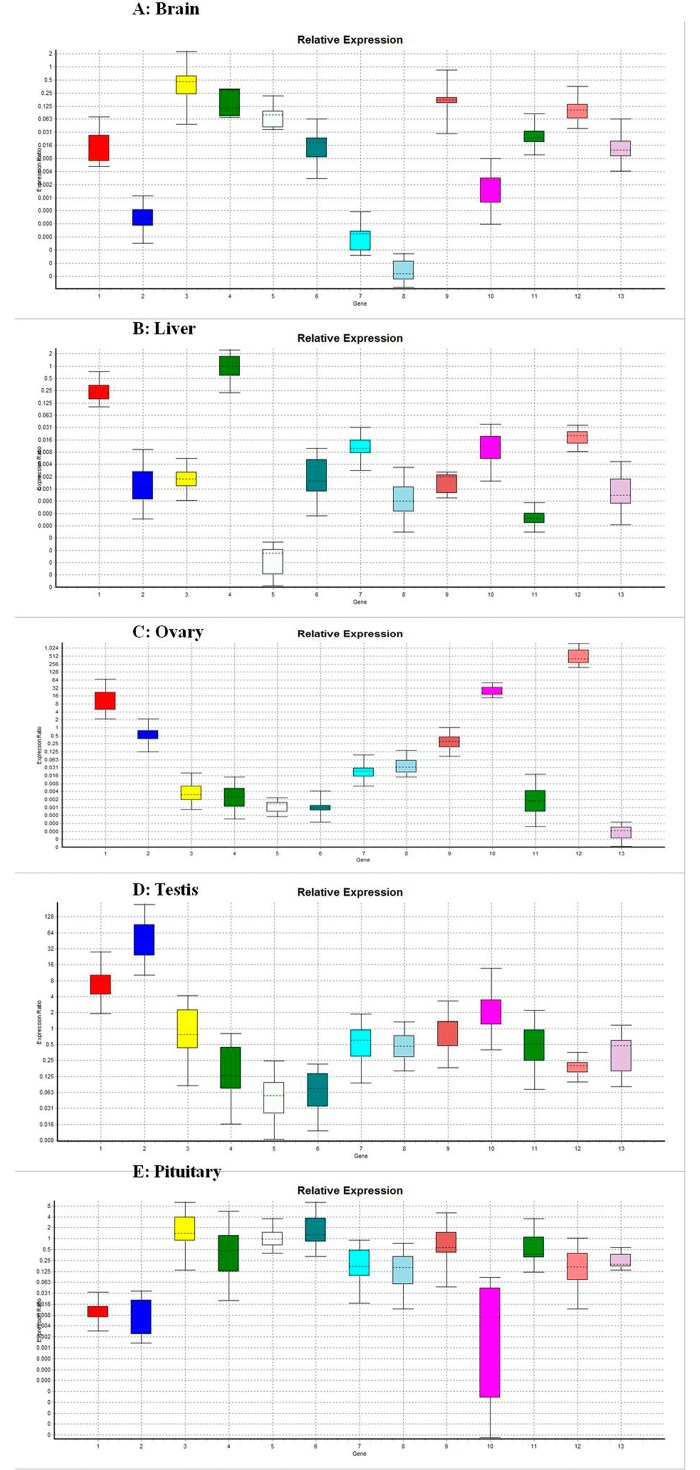
A,B,C,D,E. Comparison of relative expression ratio of 13 known transcripts in different tissues between *post-spawning* and *preparatory* phase using beta actin as reference (whisker-box plots), where the 13 transcripts are, 1 = Estrogen receptor binding site associated antigen 9 variant 1, 2 = Vitellogenin receptor, 3 = Insulin receptor b, 4 = Fibrinogen gamma chain, 5 = Green sensitive cone opsin, 6 = Steroid receptor homolog svp 46, 7 = Spermatogenic glyceraldehyde-3-phosphate dehydrogenase, 8 = Semaphorin 3fa, 9 = Follistatin-like 2, 10 = Cathepsin-Z, 11 = 11-beta-hydroxysteroid dehydrogenase, 12 = Prolactin and 13 = Activin receptor.

**Fig 7:**
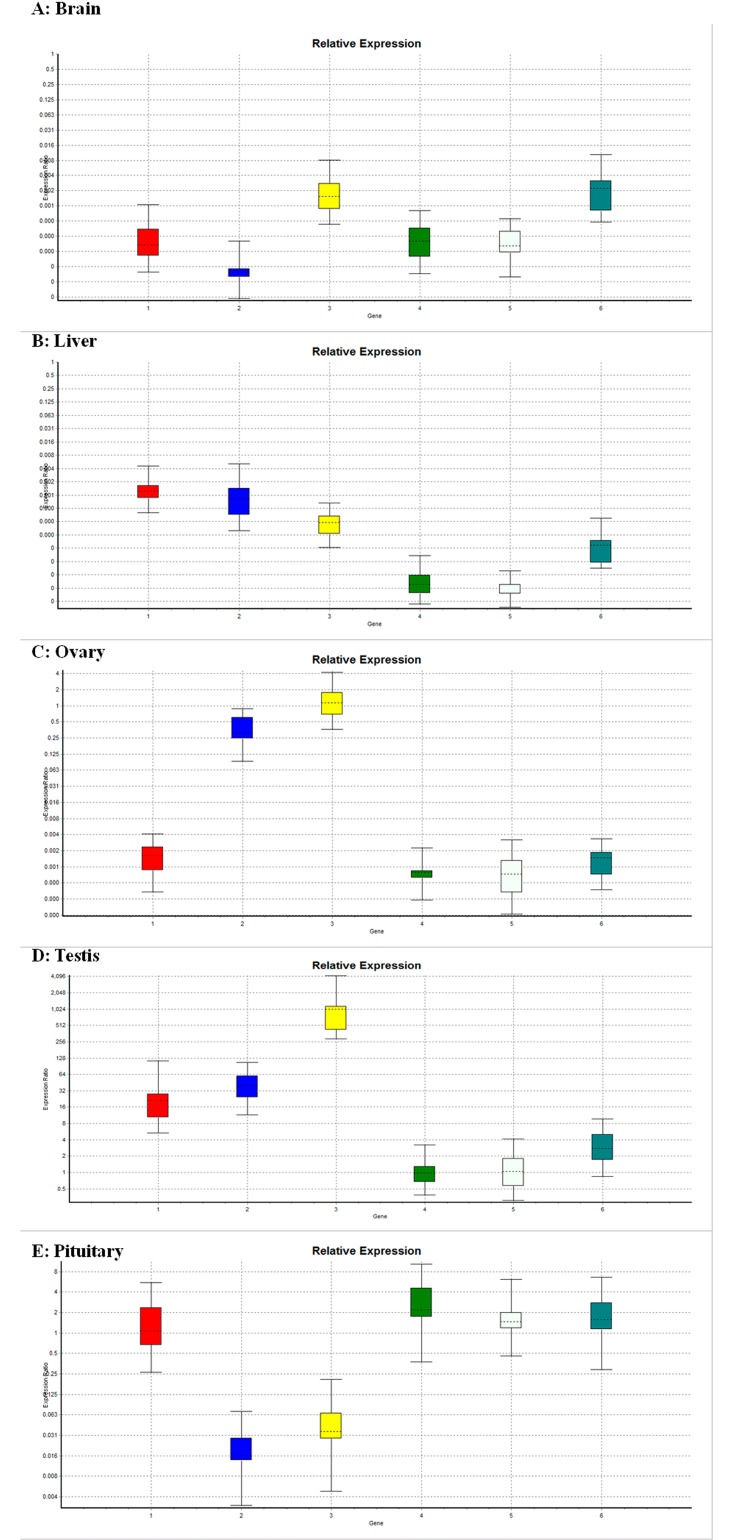
A,B,C,D,E. Comparison of relative expression ratio of 6 un-known transcripts in different tissues between *post-spawning* and *preparatory* phase using beta actin as reference (whisker-box plots), where 6 un-known transcripts are, 1 = Node 19676, 2 = Node 20067, 3 = Node 20271, 4 = Node 6976, 5 = Node 7314 and 6 = Node 19294.

Real-time RT-PCR was performed for 19 unigenes, including 13 known function categories genes (estrogen receptor binding site associated antigen 9 variant 1, vitellogenin receptor, insulin receptor b, fibrinogen gamma chain, green sensitive cone opsin, steroid receptor homolog SVP-46, spermatogenic glyceraldehydes 3-phosphate dehydrogenase, semaphorin3fa, follistatin like-2, cathepsin-Z, 11-beta-hydroxysteroid dehydrogenase, prolactin and activin receptor) (Fig 6) and 6 unknown transcripts (node-19676, node-20067, node-20271, node-6976, node-7314 and node-19294) with putative ORFs (Fig 7). Results showed clear differences in the level of expression in the same tissue between the two phases of reproduction (i.e. *preparatory* and *post-spawning* phase) in rohu. Relative expression analysis showed that expression ratio of estrogen receptor binding site associated antigen 9 variant 1 and follistatin like-2 were significantly (p<0.001 and p<0.038, respectively) up regulated only in ovary (Fig 6C), while estrogen receptor binding site associated antigen 9 variant 1 and vitellogenin receptor were up-regulated (p<0.001 and P<0.012, respectively) in testis (Fig 6D), during *preparatory* phase as compared to respective tissue levels in *post-spawning* phase. On the other hand vitellogenin receptor, fibrinogen gamma chain, green sensitive cone opsin, steroid receptor homolog SVP-46, spermatogenic glyceraldehydes 3-phosphate dehydrogenase, semaphorin3fa, follistatin like-2, cathepsin-Z, 11-beta-hydroxysteroid dehydrogenase, prolactin and activin receptor in brain (p<0.036 each) (Fig 6A), estrogen receptor binding site associated antigen 9 variant 1, vitellogenin receptor, insulin receptor b, green sensitive cone opsin, steroid receptor homolog SVP-46, spermatogenic glyceraldehydes 3-phosphate dehydrogenase, semaphorin3fa, follistatin like-2, cathepsin-Z, 11-beta-hydroxysteroid dehydrogenase, prolactin and activin receptor in liver (p<0.001 each) (Fig 6B), steroid receptor homolog SVP-46 in ovary (p<0.038) (Fig 6C), fibrinogen gamma chain, green sensitive cone opsin, steroid receptor homolog SVP-46 and prolactin in testis (p<0.048, p<0.001, p<0.048 and p<0.001, respectively) (Fig 6D), and estrogen receptor binding site associated antigen 9 variant 1, spermatogenic glyceraldehydes 3-phosphate dehydrogenase, semaphorin3fa, cathepsin-Z and activin receptor in pituitary (p<0.001, p<0.001, p<0.040, p<0.001 and p<0.042, respectively) (Fig 6E) were statistically down-regulated in *preparatory* phase in comparison to *post-spawning* phase.

Expression ratio analysis of 6 unknown putative transcripts showed that (Fig 7), node-19676, node-20067, node-20271 were significantly up-regulated in testes (p <0.001, p <0.001 and p <0.030, respectively) (Fig 7A). Almost all the unknown transcripts were down regulated in brain (p <0.001) and in liver (p <0.001, 0.034, 0.031, 0.034, 0.043 and 0.001, respectively) (Fig 7B),while node-19676, node-20067, node-20271, node-7314 and node-19294 in ovary (p <0.001, p <0.001, p <0.001, p <0.001 and p <0.039, respectively) (Fig 7C), and node-20067 and node-20271 in pituitary (p <0.034 and p <0.001, respectively) (Fig 7E), were down regulated during *preparatory* phase and the levels were statistically different from *post-spawning* phase.

Expression study of these unknown transcripts in other prolific breeder i.e. common carp in similar tissues confirms that they also possess similar sequences. Expression levels were compared between common carp and rohu (S3 Fig) in *preparatory* phase. The results showed that transcript levels of node-19676, node-20067, node-20271, node-6976, node-7314, and node-19294 in brain (p<0.001, p<0.001, p<0.001, p<0.001, p<0.001 and p<0.050, respectively), node-19676, node-20067, node-6976, node-7314 and node-19294 in liver (p<0.001, p<0.001, 6976 (p<0.001, p<0.001 and p<0.023, respectively), node-19676, node-20067, node-6976 in ovary (p<0.001, p<0.001 and p<0.034, respectively) and node-20067, node-20271 and node-6976 in pituitary (p<0.001, p<0.001 and p<0.001, respectively) were up regulated in rohu, as compared to respective tissues in common carp. On the other hand node-7314 level was significantly up regulated (p<0.001) in common carp pituitary than in rohu.

## Discussion

### Sequencing, assembly and mapping of *Labeo rohita* transcriptome

Sequencing and characterization of transcriptome of non model species using RNA-seq is one of the most important applications of NGS technologies [35]. The de novo assembly of short reads without a known reference is considered difficult [36]. In the present study different k-mer results suggested that k-mer length affects inversely to the number of contigs. Out of these the best k-mer value obtained was 37, as it resulted in highest N50 length of 1309 bp, with largest contig length of 16961 bp and average contig length of 709 bp (Fig 1). Similar findings were reported in de-novo assembly of Chickpea transcriptome [36]. 62,283 numbers of contigs with average length of 709bp as compared to 40,596 numbers of contigs with average contig length of 308bp from rohu [5], indicated better quality of reads in the present study. Out of 62,283 contigs, 50.79% (31,637) provided significant BLASTX hit corresponding to 17,925 unique protein accessions in the nr protein database, however, 47.4% contigs showing annotation from rohu in previous study [5]. It also revealed that, about 50% of transcripts did not match in any databases thus are less likely to cover coding regions, or belongs to novel sequences [37]. BLASTX top-hit species distribution of gene annotations showed highest homology with the *Danio rerio*, followed by *Oreochromis niloticus*, and *Cyprinus carpio* with *L rohita* and lesser homology with *Salmo salar*, *Ctenopharyngodon idella*, *Carassius auratus* and *Ictalurus punctatus* (Data not shown). Probably, these results indicated a high level of similarities and conserveness of the *L rohita* gene content with *Danio rerio* and *Cyprinus carpio* than with *Salmo salar* and *Ictalurus punctatus*. However, higher homology of *L rohita* (cyprinidae) sequences with *Oreochromis niloticus* (cichlidae) as compared to *Ctenopharyngodon idella* and *Carassius auratus* (cyprinidae) may be explained on the basis of the fewer number of genes that are currently available in the NCBI database for these species as was also reported in rainbow trout [37]. Blast analysis of rohu transcripts against the mRNA databases showed maximum (31,091) match with zebrafish, followed by salmon (4,894) and catfish (1,071) which further indicated closer relation of rohu with zebrafish (S1 Fig). 27,693 contigs in common carp were found significantly matching with refseq proteins of zebrafish [22]. Based on the above results rohu transcripts were chosen for chromosome wise mapping with complete genome of zebrafish (zv9), which showed that rohu transcripts were distributed in all 25 chromosomes of zebrafish (Fig 4), indicating 25 numbers of chromosomes are also present in rohu [38]. Among them, chromosome 5 and 23 of zebrafish (zv9) showed maximum similarity with rohu transcripts while it was with chromosome 7 and 5 of zebrafish (zv9) in case of common carp transcripts [19].

Out of 17,925 gene orthologues found, a total of 940 important reproduction-relevant genes under reproduction, hormone activity, receptor binding, receptor activity and embryonic development were analyzed and reported for the first time in *L rohita* from the *pre-spawning* phase. These transcripts are important because ovarian recrudescence, responsiveness and stimulatory effect to both steroidogenic and gametogenic functions of the gonad normally occurs during the *pre-spawning* period [10]. Similarly, 2852 genes involved in maturation and development of the ovary from rainbow trout [21], 474 genes from ovary of tilapia [23], 2341 genes from atlantic halibut related to quality of egg parameters [25], 1200 genes from rainbow trout and clawed toad during late oogenesis [21], 275 genes in coho salmon from ovary during primary and early secondary oocyte growth [23] were also reported.

### Analysis of reproduction relevant pathways in *Labeo rohita*

Transcriptome studies help in gene discovery and provide novel insight into various unique species-specific biological process/pathways [36]. The 8,683 sequences (Table 4) along with 328 different enzymes/orthologues representing nine important reproduction-relevant pathways obtained in KEGG analysis may serve as valuable resources for future gene identification and functional analysis, as well as development of microarray for reproduction research for this species. Among different pathways mapped, GnRH signaling is the major reproduction-relevant pathways identified. Reproduction in fishes is dependent on the coordinate actions of various hormones in which gonadotropin-releasing hormone (GnRH), acts as master regulator via the hypothalamic—pituitary—gonadal (HPG) axis [39]. Therefore GnRH signaling pathway study in this species will be of key interest in the context of its seasonal (monsoon) nature of breeding under tropical climate in comparison to prolific breeder like zebrafish [40]. Out of 121 genes mentioned in this pathway in zebrafish, about 40 genes are captured in *L rohita*, which is quite significant and not reported earlier.

Generally, immature oocytes are developed into fertilizable eggs through meiotic maturation induced by specific hormones [41]. While progesterone- mediated oocyte maturation is the major studied pathway in xenopus, it can also be induced by other steroid hormones and the pathway may differ from animal to animal [42]. 46 out of 107 genes of progesterone- mediated oocyte maturation pathway, and 53 out of 137 genes in oocyte meiosis pathway were mapped in rohu in the present study.

Steroid biosynthesis and steroid hormone biosynthesis pathways are important for fish reproduction. The steroid hormones are all derived from cholesterol [43]. Numerous organs are known to have the capacity to synthesize biologically active steroids, including the adrenal gland, testis, ovary, brain, placenta, and adipose tissue. 14 out of 28 genes from steroid biosynthesis pathway and 15 out of 38 genes from steroid hormone biosynthesis pathway of zebrafish were reported for the first time in rohu, which are of immense value for future reproductive biology study.

In retinol metabolism pathways, retinal is the predominant retinoid in eggs and oocytes of marine fish as well as some freshwater fish species and may constitute almost the entire pool of retinoids in these eggs [44]. The cellular pathways for retinoid metabolism are mainly known from studies in mammalian species. These pathways are highly conserved and principally identical among all classes of vertebrates, probably also in fish [44]. Out of 38 genes from retinol metabolism pathways of zebrafish, 17 genes were observed in *L rohita*.

Similarly neurotrophins are growth factors implicated in the development and maintenance of different neuronal populations in the nervous system [45]. No neurotrophin signaling pathways are reported for zebrafish in KEGG database; however, in the present study, 67 genes involved in neurotrophin signaling pathways for rohu were found.

Photoperiodic manipulation has emerged as an effective tool of reproductive management in culture fisheries, and understanding the physiology of photoperiodic regulation of fish reproduction became the priority topic of research in different countries [11]. So study of photo-transduction pathways for this species is of immense value. Recently, significant advancement of gonad maturation of Indian major carp species (rohu, catla and mrigal) was possible through photothermal manipulation [14]. For the photo-transduction pathway in rohu, out of 38 genes, 13 genes were mapped from KEGG pathway databases.

### Expression analysis of reproduction relevant genes by real time PCR

Some of the important gene orthologues i.e, vitellogenin receptor, insulin receptor b, fgg protein, green sensitive cone opsin, steroid receptor homolog svp 46, prolactin, activin receptor, spermatogenic glyceraldehyde-3-phosphate dehydrogenase, semaphorin 3fa, follistatin-like 2, cathepsin- Z and estrogen receptor binding site associated antigen 9 variant 1, were analyzed by qPCR in *preparatory* and *post-spawning* period. All these transcripts were collected during *pre-spawning* phase, but *preparatory* and *post-spawning* phases are equally important to know the events in initiation of gonad maturation in Indian carps [14] and accumulation of any transcript in these stages indicate its role in maturation.

Vitellogenin synthesized and released from the liver, is carried through blood and taken up into oocytes by the vitellogenin receptor; which is an essential process in oviparous animals to ensure successful reproduction [46]. Although different isoform of vitellogenins [47] and their role in gonad maturation [48] are found in the literature, nothing is reported about vitellogenin receptor and their role. Expression of vitellogenin receptor was down regulated in most of the tissues studied (except in testis) in *preparatory* phase as compared to *post-spawning* in *L rohita*. Amounts of vitellogenin receptor were less in individuals with yolked oocytes (ripening stage, May-June) and increased after spawning in July in Atlantic bluefin tuna (*Thunnus thynnus L*.) [49]. Generally Insulin is implicated in growth, development and reproduction in teleosts [50] and expression of different insulin receptor genes were studied [49]. In *L rohita* expression of insulin receptor b was found down regulated particularly in liver and almost no change in other tissues during *preparatory* phase as compared to *post-spawning*, whereas high level of Insulin receptor b mRNA was reported in ovary in rainbow trout [51]. The role of fibrinogen-γ (FGG) in uterine epithelial cells during normal pregnancy, pseudo-pregnancy and in hormone-treated rats is suggested [52]. Fibrinogen gamma chain expression during *preparatory* phase was significantly lower in brain and testis than in *post-spawning* phase and no variation observed in other tissues in rohu. However, in zebra fish higher expression was observed in the embryonic yolk syncytial layer than in the early cells of the developing liver [53]. Photoreceptors mainly consist of rods and cones. In the retina of diurnal primates, cones are further subdivided into three subtypes, the red-, green-, and blue-sensitive cones, whose visual pigments are maximally sensitive to long, middle, and short wavelengths, respectively [54]. Significantly lower expression of green sensitive cone opsin was observed in brain, liver and testis during *preparatory* phase in *L rohita*, where as expression of Pi-green1 and Pi-green2 cone opsins were observed in skin and lateral eyes in *Paracheirodon innesi* [55]. Steroid receptor homolog svp 46 showed significantly reduced expression in brain, liver and testis while spermatogenic glyceraldehyde-3-phosphate dehydrogenase (GAPDH) and Semaphorin 3fa both were down regulated in brain, liver and pituitary during *preparatory* phase in *L rohita*. In human GAPDH protein was expressed in both sertoli cells and elongated sperms [56], whereas high expression levels of semaphorin 3fa domains were observed in human oocyte from the earliest follicle stages [57] but no report was available about their expression level in any fish species. Expression level of follistatin-like 2 was significantly lower in ovary during *preparatory* phase, whereas in xenopus it is reported as an early gastrula expressed gene [58]. Cathepsin-Z level was significantly higher in ovary in *post-spawning* rohu while abundant cathepsin-Z expression was observed in rainbow trout in low quality unfertilized eggs [59]. In most vertebrates, 11-beta-hydroxysteroid dehydrogenase b2 is essential for conferring aldosterone-specific actions in mineralocorticoid target tissues and for protecting glucocorticoid-sensitive tissues during stress [44]. Expression level of 11-beta-hydroxysteroid dehydrogenase was significantly less in brain and liver during *preparatory* phase in rohu; whereas expression of this transcript was observed nearly in all peripheral tissues in zebrafish [44]. Prolactin plays important roles in freshwater fish reproduction [60] and seasonal acclimatization [61]. Expression level of prolactin was significantly lower in brain, liver and testis during *preparatory* phase compared to *post-spawning* in *L rohita*. However in *Cyprinus carpió* higher level of mRNA expression was noticed in summer carp pituitary, as compared to winter carp [61]. Activins are critical components of the signaling network that controls female reproduction and different receptors control their roles and functions in hypothalamus [62]. Activin receptor level was significantly low in brain, liver and pituitary during *preparatory* phase of *L rohita*, whereas in *Ctenopharyngodon idella* activin receptor transcripts shows high expression levels in extra-gonadal tissues, including pituitary, brain, and liver [63]. Report of mRNA encoding estrogen receptor binding site associated antigen 9 variant 1 is present in mammalian oocytes [64], but nothing is known about either expression pattern or function in oocytes during maturation, fertilization, and subsequent embryonic development. A significantly higher expression level of this transcript was found in ovary and testis in *preparatory* rohu in the present study.

Almost all the unknown transcripts were down regulated in brain, liver and ovary (Fig 7), and node-19676, node-20067 and node-20271 were up-regulated in testes during *preparatory* phase. Many unknown transcripts or novel sequences (34.7%) were also reported during transcriptomic analyses in zebrafish gonad and brain [65]. Searching of similar sequences for the unknown transcripts in the present database of common carp was not successful, and always showing only a few bases matching both at BLASTN, tBLASTN as well as in BLASTX and BLASTP as was reported in our previous study [6], but expression of all these transcripts are also observed in these prolific breeders and significantly higher expression (p<0.001) of node-7314 was noticed in common carp pituitary. Level of expression of almost all the 6 unknown transcripts were significantly higher in seasonal breeder rohu in comparison to carp which indicates that there may be some possible role of these transcripts in reproduction of rohu as was reported in our previous study also [6].

### Development of EST-SSR markers and SNPs and validation of EST-SSR markers

Identification of large number of SSRs in the *L rohita* transcripts with frequency of one SSR per 3.41 kb of the sequence is very interesting and will enrich the existing marker resources of rohu to facilitate genetic improvement of this species.

Twelve microsatellite loci associated with the genes involved in reproductive process showed polymorphism in the present study out of 29 identified, while twenty repeat loci were reported to be polymorphic from 128 loci in our previous study [6]. The higher percentage of polymorphic loci in this study could be due to the high polymorphism between mapping parents. However, a high attrition rate of potential microsatellite marker is generally observed in case of EST-SSRs, starting from primer design step following identification of a simple sequence repeat, through agarose gel analysis, to polyacrylamide gel optimization and final analysis. The relatively low success rate of primers showing good amplification may be due at least in part to high intra-specific polymorphism in the *L rohita* genome, as was seen in the analysis of flanking region sequences in this study. Similarly Cameron et al., [66] attributed variation in amplification efficiency of sea urchin microsatellite loci to a high level of genomic polymorphism.

Presence of repeats in some of the corresponding zebrafish sequences indicated that SSR may be present in these sequences across the species although it could not be confirmed in common carp due to lack of sequences in databases. A total of 52925 SNPs including 1827 homozygous and 51098 heterozygous were identified in rohu by comparing with zebrafish (zv9) genome, as no reference sequences are reported for rohu in databases. SNPs were reported for disease resistant and susceptible lines of rohu [17] but the SNPs detected in the present study need further validation.

### Identification of Isoforms

It has been suggested that assembly of velvet followed by oases yields better contigs/transcripts to produce transcript isoforms [36]. Production of 88, 612 transcripts by oases analysis in comparison to the 62,283 contigs resulted in velvet, suggest that rohu transcript showing isoforms. Observation of gene isoforms from reproduction related transcripts in *L rohita* is an interesting finding. Seven isoforms of activin receptors were found in rohu, while six variants were observed in grass carp [63]. TATA box binding proteins have two isoforms in rohu; similar splice variant was observed in human, encoding the polyglutamine-containing N-terminal domain that accumulates in Alzheimer's disease [67]. However no isoforms for these proteins were reported in any other fish species. Transferrin receptor showed two isoforms in rohu, which is quite similar with other vertebrates [68]. Thyroid hormone receptor showed seven isoforms in rohu while two thyroid hormone receptor-α genes (thraa, thrab) were found in zebrafish [69]. Seven isoforms of progesterone receptor membrane component was observed in rohu in contrast to three forms reported in channel catfish [70]. Six, four and three isoforms are observed for Prostaglandin synthase, Beta-galactosyltransferase and Semaphorin respectively in rohu, but no isoforms are reported in other fish species. Three isoforms were found for retinoic acid receptor in rohu as compared to seven isoforms in zebrafish [71]. Six isoforms for estrogen-related receptor and two isoforms for cadherin were found in rohu, number of which varies in mammals [72] and zebrafish [73]. Present study revealed six isoforms of insulin receptor in rohu, whereas zebrafish expressed two isoforms of it (insra and insrb) [74]. Nuclear receptors are a class of proteins found within cells that are responsible for sensing steroid and thyroid hormones and certain other molecules and in rohu twelve isoforms of nuclear receptor were observed. Talin showed two isoforms in rohu, where as talin-1 and talin-2 in model vertebrates produces two talins through alternative mRNA splicing [75]. DNA methyltransferase showed two isoforms both in rohu and zebrafish [76]. It is necessary to mention here that these isoforms in rohu are the product of next generation sequencing in which the sequences were generated as short read 75bp sequence followed by assembly of them by the software. Therefore validity of all these isoforms are highly essential for final conclusion.

## Conclusion

Production of 62,283 high-quality *L rohita* transcriptome derived from brain, pituitary, liver, intestine, kidney, tongue, nose, eye, gill, muscle, heart ovary and testis tissues from *pre-spawning* phase will contribute a significant non-redundant set of ESTs resources. Out of 17,925 important gene orthologues found, a total of 940 reproduction-relevant genes were analyzed and reported for the first time in *L rohita*. In KEGG analysis, 8,683 well-categorized, annotated transcriptome along with 328 different enzymes/orthologues representing nine important reproduction-relevant pathways were obtained. A total of 22,383 SSRs were identified in 17,244 transcripts of rohu and 12 polymorphic loci were identified from 29 reproduction related genes. Difference in tissue expression levels of 13 known genes and 6 unknown putative genes indicates variation between *preparatory* and *post-spawning* phase of these transcripts in monsoon breeder carp *L rohita*. Isoforms for several reproduction related gene transcripts in *L rohita* is an interesting finding. These data may serve as important and valuable resources for *L rohita* genetics and genomics which will be beneficial as a reference set for the production of large-scale transcriptome study in future as well as for gene identification and functional analysis for rohu reproduction.

## Supporting Information

S1 Fig Mapping of rohu transcripts with zebra fish, salmon, and catfish Bottom of Form mRNA. (TIF)

S2 Fig Distribution of SSRs. (TIF)

S3 Fig Comparison of tissue expression ratio of 6 un-known transcripts between rohu and common carp in brain, pituitary, ovary and liver tissues during *preparatory* phase using beta actin as reference (whisker-box plots). (TIF)

S1 Table Transcript specific Forward (F) and reverse (R) primers used in real-time PCR. (DOCX)

S2 Table List of reproduction-relevant transcripts identified in *Labeo rohita*. (DOC)

S3 Table Comparison of repeat types identified in *L*. *rohita* with corresponding gene sequences of *Cyprinus carpio* and *Danio rario*. (DOCX)

S4 Table List of SNPs identified from rohu contigs. (XLSX)
