# Supplementary figures and images for: Construction, De-Novo Assembly and Analysis of Transcriptome for Identification of Reproduction-Related Genes and Pathways from Rohu, Labeo rohita (Hamilton)
Source: PLoS One. 2015 Jul 6;10(7):e0132450. doi: 10.1371/journal.pone.0132450 (PMC4509579; doi:10.1371/journal.pone.0132450)

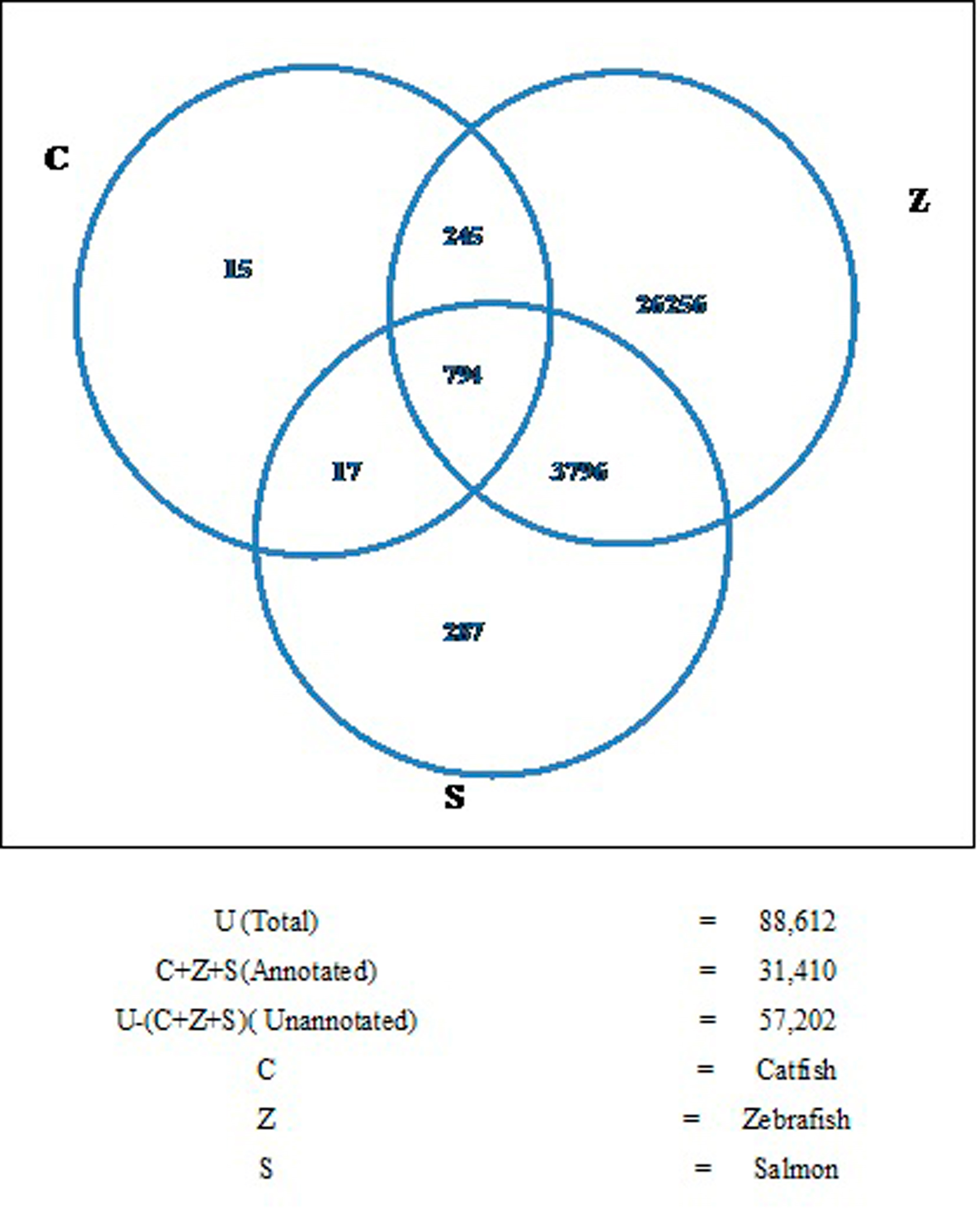

Supplement: S1 Fig — (TIF) [file pone.0132450.s001.tif]

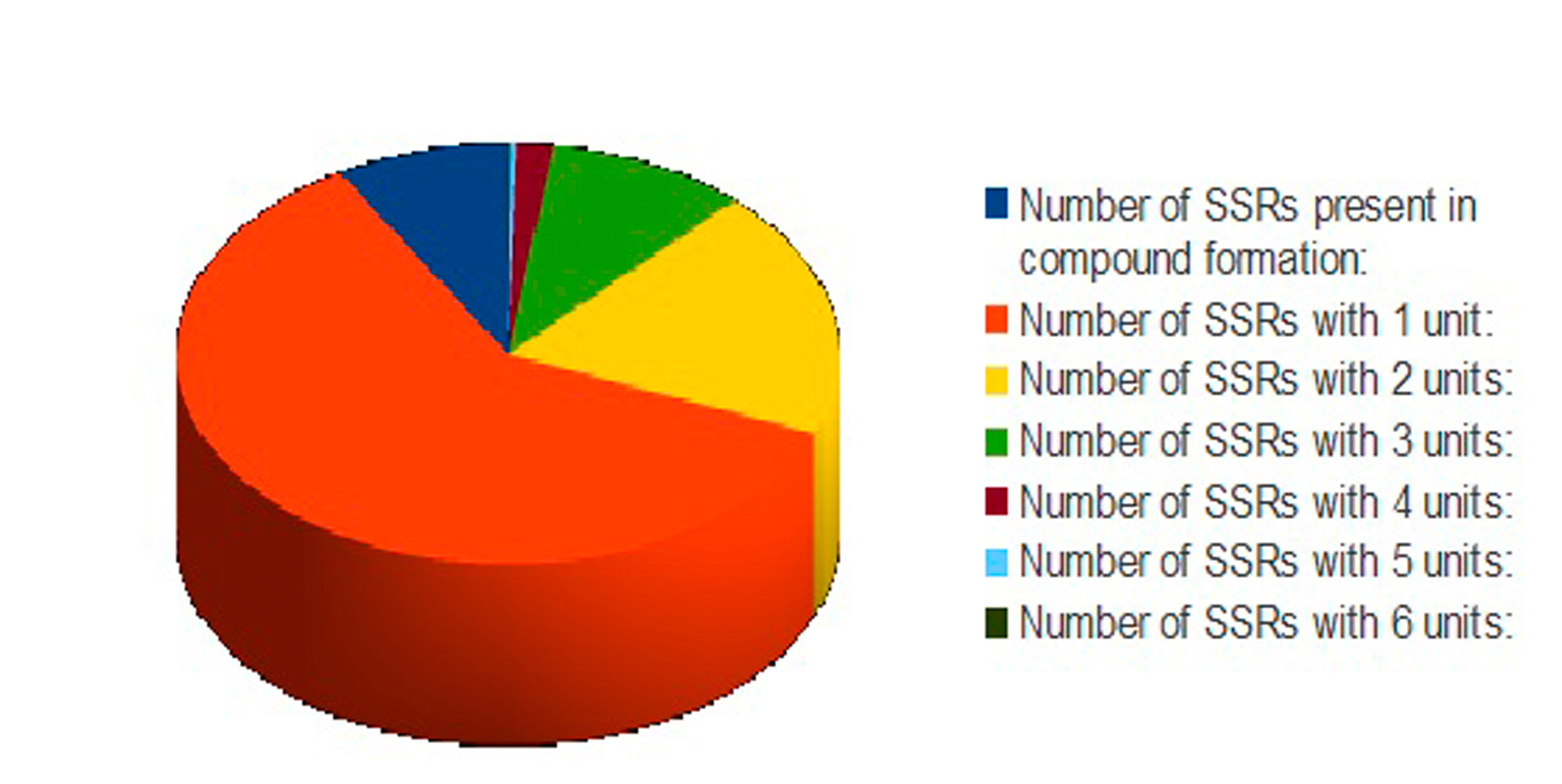

Supplement: S2 Fig — (TIF) [file pone.0132450.s002.tif]

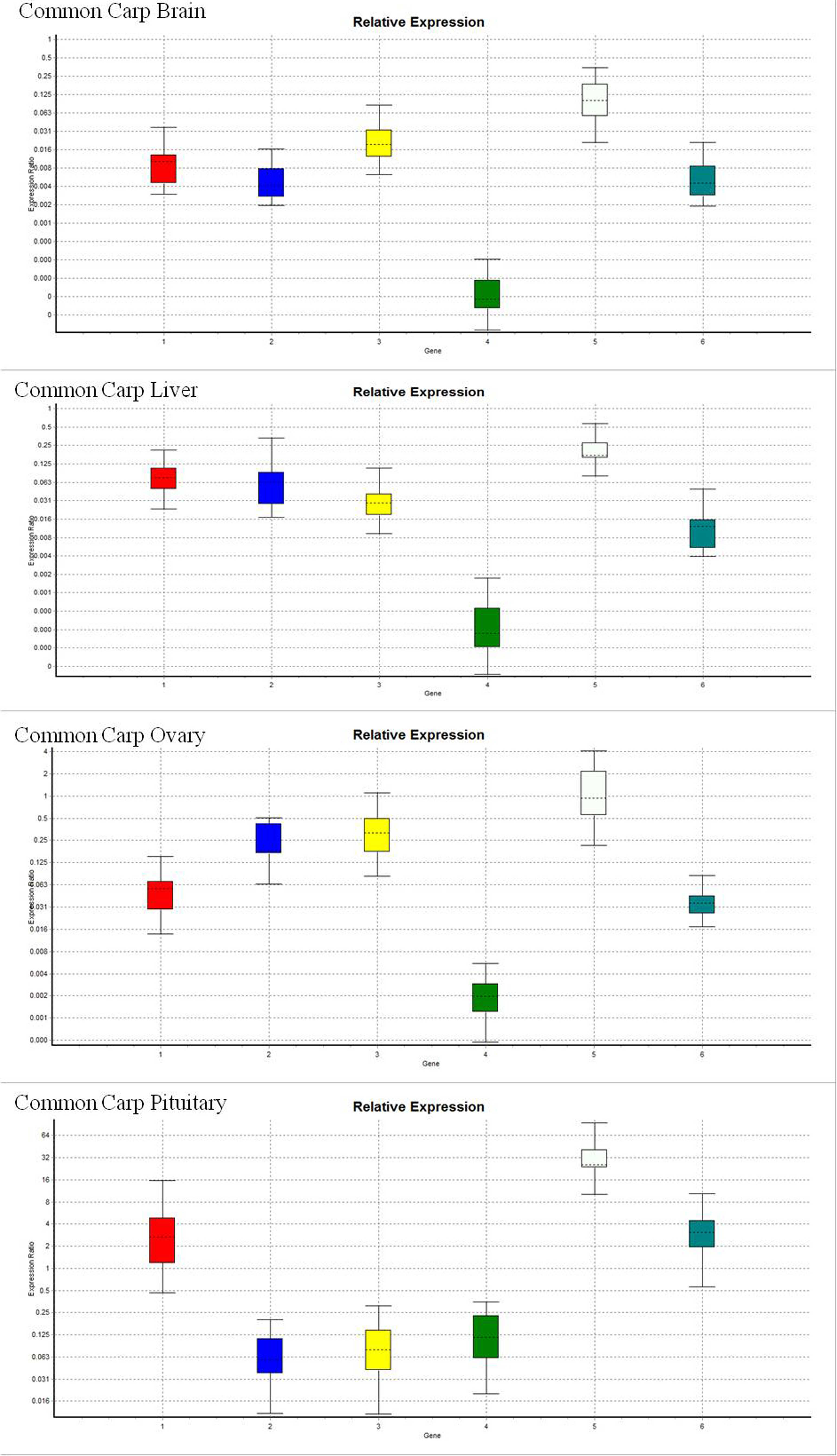

Supplement: S3 Fig — (TIF) [file pone.0132450.s003.tif]
