# Supplementary material for: Construction, De-Novo Assembly and Analysis of Transcriptome for Identification of Reproduction-Related Genes and Pathways from Rohu, Labeo rohita (Hamilton)
Source: PLoS One. 2015 Jul 6;10(7):e0132450. doi: 10.1371/journal.pone.0132450 (PMC4509579; doi:10.1371/journal.pone.0132450)
Supplement: S1 Table — (DOCX) [file pone.0132450.s004.docx]

S1 Table. Transcript specific Forward (F) and reverse (R) primers used in real-time PCR.

| **Sl No.** | **Transcript** | **Primer sequence (5’ *→*3’)** | **Tm (*^◦^*C)** | **Cycles** | **Reaction Efficiency** |
| --- | --- | --- | --- | --- | --- |
| **Known transcript** | | | | | |
|  | β*-*actin | F: TTCGAGCAGGAGATGGGCACTG  R: GCATCCTGTCAGCAATGCCA | 62 | 40 | 1.0 |
|  | Estrogen receptor binding site associated antigen 9 variant 1 | F: GCCATCACACAGTTTCGCCT  R: TCCCATGAGCTCCACTCCTC | 58 | 40 | 0.98 |
|  | Vitellogenin receptor | F: TCAGGGACGAGGCCACAGACA  R: CCGTCGCACTGCCACACAGA | 58 | 40 | 0.97 |
|  | Insulin receptor b | F: CCCGGGGTCAAAATGCAGCT  R: GGGGTAACTCAGGCCACGGA | 58 | 40 | 0.98 |
|  | Fibrinogen gamma chain | F:ACGCCACTTTCAAACTTGGACCA  R: CAGCGGTTCATCCACCAGCCAG | 58 | 40 | 0.94 |
|  | Green sensitive cone opsin | F: GGGCAAAGATGAACGGCACT  R: GGTACCAGCCACAGCCAGGT | 58 | 40 | 0.99 |
|  | Steroid receptor homolog SVP-46 | F: AGAAGCTCTTGCAGCCCTCG  R: CCACAGAACGTGTTGCGGGA | 58 | 40 | 0.93 |
|  | Spermatogenic glyceraldehydes 3-phosphate dehydrogenase | F: GGTAGGGCTTTTCTGCTGTCGCA  R: ATGCCGGTGCAGGCATTTCCCT | 58 | 40 | 0.97 |
|  | Semaphorin3fa | F: CGTCCCCGTGATGTCTGCGA  R: GCAGCTTCCTCGCTCCAGTC | 58 | 40 | 0.98 |
|  | Follistatin like-2 | F: GGAGAACAACCGCCTGCTCA  R: GTCATGGCAGTGCAGACCCAG | 58 | 40 | 0.99 |
|  | Cathepsin-Z | F: GCCCTTGTATGCGCTGGTGAC  R: AGGACCCATCAGCTGTGGCA | 58 | 40 | 0.98 |
|  | 11-beta-hydroxysteroid dehydrogenase | F:TGGAGGAACCTGTCGCTGACG  R:AGCCAATGAGGATCTGAGCCCTG | 58 | 40 | 0.99 |
|  | Prolactin | F: CCATTCACTCCAGAGGCCTTGG  R: TTCACGCAGAACGCGAACAGTC | 58 | 40 | 0.99 |
|  | Activin receptor | F: GTAAGGCTCCCTTCAGCGACAC  R: ATCGGTCCAGGGTCCTGGGT | 58 | 40 | 0.97 |
|  |  |  |  |  |  |
| **Un-known transcript having putative ORFs** | | | | | |
|  | Node-19676 | F:CGTCCTGTTCACTGCACCCAG  R:ATGCCACAGCAGACGTCGCT | 58 | 40 | 0.99 |
|  | Node-20067 | F:GGCTCTATACCAGGTGGTGCA  R:CAGTGGTTCTGGAGTCATGCAGT | 58 | 40 | 0.98 |
|  | Node-20271 | F:TGCAGGTCTCTGTGGTGGTG  R:ACAGCTGGATGCTGGGCAGT | 58 | 40 | 1.0 |
|  | Node-6976 | F: CAGATATGGCCCCAGGACAA  R:TCCAGTGGAAGTAACTGGCACT | 58 | 40 | 0.95 |
|  | Node-7314 | F: ACCTGGTTAGTTTGCGGCGTA  R: TAGAGCACAAGGCGCTGGAT | 58 | 40 | 0.98 |
|  | Node-19294 | F: AGCAGGAAGGATTTGAGGCACT  R: GGGCGCACTTTGCTTCCTCT | 58 | 40 | 0.94 |
