# Supplementary material for: Construction, De-Novo Assembly and Analysis of Transcriptome for Identification of Reproduction-Related Genes and Pathways from Rohu, Labeo rohita (Hamilton)
Source: PLoS One. 2015 Jul 6;10(7):e0132450. doi: 10.1371/journal.pone.0132450 (PMC4509579; doi:10.1371/journal.pone.0132450)
Supplement: S2 Table — (DOC) [file pone.0132450.s005.doc]

S2 Table. List of reproduction-relevant transcripts identified in *Labeo rohita*

| **Sl. No.** | **Gene identity** | | **Transcripts ID** | **Length**  **(bp)** | **Expect** | | |  | | | | |
| --- | --- | --- | --- | --- | --- | --- | --- | --- | --- | --- | --- | --- |
| ***Reproduction related proteins*** | | |  |  |  | | |  | | | | |
|  | Member ras oncogene family | | NODE_11_length_951_cov_715.494202 | 987 | 1.4E-146 | | |  | | | | |
|  | Dihydrolipoamide dehydrogenase | | NODE_507_length_1613_cov_196.046494 | 1649 | 0.0 | | |  | | | | |
|  | Cxcr4b protein | | NODE_559_length_871_cov_817.191711 | 907 | 1.1E-106 | | |  | | | | |
|  | Histone deacetylase 1 | | NODE_797_length_1425_cov_131.437195 | 1461 | 0.0 | | |  | | | | |
|  | tacc3 protein | | NODE_799_length_411_cov_94.447685 | 447 | 9.9E-61 | | |  | | | | |
|  | Moloney leukemia virus 10-like homolog | | NODE_1203_length_2173_cov_30.269672 | 2209 | 0.0 | | |  | | | | |
|  | Gametogenetin binding protein 2 | | NODE_1237_length_2665_cov_52.878426 | 2701 | 0.0 | | |  | | | | |
|  | nme2 protein | | NODE_1513_length_97_cov_757.103088 | 133 | 3.7E-22 | | |  | | | | |
|  | Novel protein vertebrate upstream transcription factor c-fos interacting | | NODE_2007_length_1568_cov_5.557398 | 1604 | 2.6E-73 | | |  | | | | |
|  | c-terminal binding protein 1 | | NODE_2028_length_1003_cov_24.352942 | 1039 | 3.5E-160 | | |  | | | | |
|  | oep protein | | NODE_2201_length_1293_cov_15.348802 | 1329 | 1.5E-103 | | |  | | | | |
|  | Membrane progestin receptor alpha | | NODE_2486_length_1360_cov_7.952206 | 1396 | 0.0 | | |  | | | | |
|  | Novel protein vertebrate polymerase (DNA directed) epsilon | | NODE_2485_length_2153_cov_12.154203 | 2189 | 0.0 | | |  | | | | |
|  | h aca ribonucleoprotein complex non-core subunit naf1 | | NODE_2498_length_1449_cov_32.386475 | 1485 | 9.3E-146 | | |  | | | | |
|  | Histone-arginine n-methyltransferase prmt7 | | NODE_2513_length_1750_cov_28.462856 | 1786 | 0.0 | | |  | | | | |
|  | Discoidin domain receptor member 1 | | NODE_2837_length_4085_cov_9.552754 | 4121 | 0.0 | | |  | | | | |
|  | Sperm associated antigen 9 | | NODE_2964_length_1958_cov_22.445353 | 1994 | 0.0 | | |  | | | | |
|  | af473824_1 hepatocyte nuclear factor 4 alpha | | NODE_3009_length_2219_cov_22.456963 | 2255 | 0.0 | | |  | | | | |
|  | b chain n-terminal domain of lissencephaly-1 protein (lis-1) | | NODE_3165_length_526_cov_41.359314 | 562 | 2.5E-34 | | |  | | | | |
|  | tnfaip3 interacting protein 1 | | NODE_3282_length_162_cov_151.271606 | 198 | 9.0E-26 | | |  | | | | |
|  | ATP-dependent dna helicase pif1 | | NODE_3433_length_2416_cov_101.633690 | 2452 | 0.0 | | |  | | | | |
|  | Ubiquitin-conjugating enzyme e2b | | NODE_3585_length_857_cov_31.256710 | 893 | 1.6E-106 | | |  | | | | |
|  | Insulin-degrading enzyme | | NODE_3731_length_2713_cov_127.505714 | 2749 | 0.0 | | |  | | | | |
|  | Nucleosome assembly protein 1-like 1 | | NODE_3809_length_753_cov_323.112885 | 789 | 1.9E-97 | | |  | | | | |
|  | Rad23 homolog b | | NODE_4108_length_203_cov_116.837440 | 239 | 1.5E-35 | | |  | | | | |
|  | Cell differentiation protein rcd1 homolog | | NODE_4438_length_1109_cov_43.220016 | 1145 | 0.0 | | |  | | | | |
|  | Platelet-activating factor isoform alpha subunit a | | NODE_4697_length_1684_cov_55.204868 | 1720 | 0.0 | | |  | | | | |
|  | cxcr7b protein | | NODE_5512_length_633_cov_48.199051 | 669 | 1.3E-123 | | |  | | | | |
|  | acvr1 activin a type i | | NODE_5557_length_4000_cov_26.475000 | 4036 | 0.0 | | |  | | | | |
|  | Spermatid perinuclear RNA-binding protein | | NODE_5578_length_2723_cov_38.104298 | 2759 | 0.0 | | |  | | | | |
|  | af364811_1 e-cadherin | | NODE_5704_length_207_cov_7.275362 | 243 | 2.3E-32 | | |  | | | | |
|  | eif2b5 protein | | NODE_5917_length_2495_cov_23.915833 | 2531 | 0.0 | | |  | | | | |
|  | UV excision repair protein rad23 homolog b-like isoform 3 | | NODE_6459_length_817_cov_83.768669 | 853 | 6.8E-61 | | |  | | | | |
|  | Xanthine dehydrogenase oxidase | | NODE_6691_length_325_cov_32.446156 | 361 | 1.6E-65 | | |  | | | | |
|  | dazl protein | | NODE_6808_length_1415_cov_60.763252 | 1451 | 3.9E-150 | | |  | | | | |
|  | Zinc finger protein isoform cra_d | | NODE_7906_length_3089_cov_11.035933 | 3125 | 0.0 | | |  | | | | |
|  | Mediator complex subunit 1 | | NODE_7926_length_5143_cov_13.350768 | 5179 | 0.0 | | |  | | | | |
|  | Forkhead box o5 | | NODE_8161_length_2145_cov_91.134735 | 2181 | 0.0 | | |  | | | | |
|  | Peptidylglycine alpha-amidating isoform cra_b | | NODE_8529_length_3314_cov_55.052505 | 3350 | 0.0 | | |  | | | | |
|  | Seven in absentia homolog 1 | | NODE_9008_length_1599_cov_16.423389 | 1635 | 0.0 | | |  | | | | |
|  | Zgc:66298 protein | | NODE_9124_length_3775_cov_15.087682 | 3811 | 0.0 | | |  | | | | |
|  | Prospero-related homeodomain protein 1 | | NODE_9264_length_1698_cov_8.459364 | 1734 | 0.0 | | |  | | | | |
|  | Subfamily member 1 | | NODE_9826_length_2430_cov_28.332098 | 2466 | 0.0 | | |  | | | | |
|  | Centromere protein i | | NODE_10171_length_780_cov_31.092308 | 816 | 6.0E-132 | | |  | | | | |
|  | V-akt murine thymoma viral oncogene homolog 1 | | NODE_10336_length_2899_cov_15.776475 | 2935 | 0.0 | | |  | | | | |
|  | Homolog 2 ( coli) | | NODE_11079_length_2805_cov_35.903744 | 2841 | 0.0 | | |  | | | | |
|  | cAMP-responsive element modulator-like isoform 7 | | NODE_11271_length_358_cov_257.784912 | 394 | 1.6E-48 | | |  | | | | |
|  | Dynein light chain cytoplasmic | | NODE_11550_length_733_cov_32.725784 | 769 | 4.4E-58 | | |  | | | | |
|  | brd2b protein | | NODE_11865_length_1528_cov_18.842278 | 1564 | 4.3E-91 | | |  | | | | |
|  | hsf2 protein | | NODE_11965_length_773_cov_53.031048 | 809 | 1.6E-139 | | |  | | | | |
|  | Rearranged l-myc fusion | | NODE_12695_length_1357_cov_24.341930 | 1393 | 0.0 | | |  | | | | |
|  | b-cell leukemia lymphoma 6 | | NODE_13079_length_1789_cov_26.882616 | 1825 | 0.0 | | |  | | | | |
|  | cre-xnp-1 protein | | NODE_13115_length_880_cov_6.789773 | 916 | 2.4E-26 | | |  | | | | |
|  | Tudor domain-containing protein 1 | | NODE_13783_length_3757_cov_12.966462 | 3793 | 0.0 | | |  | | | | |
|  | Transforming growth factor-beta receptor type i a | | NODE_14140_length_2448_cov_8.219771 | 2484 | 0.0 | | |  | | | | |
|  | Chemokine (c-x-c motif) ligand 12b (stromal cell-derived factor 1) | | NODE_14156_length_1072_cov_9.876865 | 1108 | 5.9E-45 | | |  | | | | |
|  | ptk2b protein tyrosine kinase 2 isoform cra_b | | NODE_14381_length_3391_cov_22.592745 | 3427 | 0.0 | | |  | | | | |
|  | Bone morphogenetic protein type | | NODE_14540_length_3724_cov_8.004565 | 3760 | 0.0 | | |  | | | | |
|  | Testis expressed gene 15 | | NODE_15063_length_4005_cov_8.859176 | 4041 | 0.0 | | |  | | | | |
|  | Chemokine (c-x-c motif) receptor 4a | | NODE_15345_length_1506_cov_34.626163 | 1542 | 0.0 | | |  | | | | |
|  | Bardet-biedl syndrome 4 | | NODE_16118_length_1487_cov_6.589778 | 1523 | 0.0 | | |  | | | | |
|  | Outer dense fiber of sperm tails 2 | | NODE_16526_length_1920_cov_7.717708 | 1956 | 0.0 | | |  | | | | |
|  | Homolog colon nonpolyposis type 2 ( coli) | | NODE_16542_length_2451_cov_27.105671 | 2487 | 0.0 | | |  | | | | |
|  | Upstream binding protein 1 (lbp-1a) isoform cra_a | | NODE_16659_length_2386_cov_40.985332 | 2422 | 8.1E-134 | | |  | | | | |
|  | Cyclin d1 | | NODE_17074_length_2575_cov_10.192233 | 2611 | 0.0 | | |  | | | | |
|  | piwi-like protein 1 | | NODE_17427_length_3336_cov_7.598921 | 3372 | 0.0 | | |  | | | | |
|  | Zinc finger protein isoform cra_b | | NODE_17803_length_875_cov_8.627429 | 911 | 4.9E-141 | | |  | | | | |
|  | Ribosomal protein s6 kinase polypeptide 1 | | NODE_17810_length_3995_cov_15.135920 | 4031 | 0.0 | | |  | | | | |
|  | Zgc:111976 protein | | NODE_19529_length_152_cov_46.684212 | 188 | 10.0E-32 | | |  | | | | |
|  | Phosphate cytidylyltransferase beta isoform | | NODE_19724_length_2200_cov_21.749090 | 2236 | 5.3E-174 | | |  | | | | |
|  | Conserved helix-loop-helix ubiquitous kinase | | NODE_19778_length_3003_cov_29.223444 | 3039 | 0.0 | | |  | | | | |
|  | bmp15 protein | | NODE_20193_length_1994_cov_24.159981 | 2030 | 0.0 | | |  | | | | |
|  | c-terminal binding protein 2 | | NODE_20227_length_965_cov_14.400000 | 1001 | 2.4E-170 | | |  | | | | |
|  | Piwil2 protein | | NODE_20358_length_2541_cov_7.066116 | 2577 | 0.0 | | |  | | | | |
|  | Upstream binding protein 1 (lbp-1a) isoform cra_b | | NODE_20599_length_1349_cov_24.236471 | 1385 | 3.6E-161 | | |  | | | | |
|  | Activin a type 1 | | NODE_20604_length_1463_cov_14.717704 | 1499 | 0.0 | | |  | | | | |
|  | Upstream transcription factor isoform cra_a | | NODE_20631_length_343_cov_56.332363 | 379 | 2.7E-82 | | |  | | | | |
|  | Xanthine dehydrogenase | | NODE_20726_length_3086_cov_15.562864 | 3122 | 0.0 | | |  | | | | |
|  | Glutathione mitochondrial | | NODE_20829_length_1974_cov_19.965551 | 2010 | 0.0 | | |  | | | | |
|  | Diaphanous homolog 2 | | NODE_20847_length_2148_cov_12.801676 | 2184 | 0.0 | | |  | | | | |
|  | Ubiquitin protein ligase E3A | | NODE_20964_length_2962_cov_39.428425 | 2998 | 0.0 | | |  | | | | |
|  | Novel protein vertebrate glutamate receptor interacting protein | | NODE_21274_length_1846_cov_21.080173 | 1882 | 0.0 | | |  | | | | |
|  | Zinc finger matrin-type protein | | NODE_21813_length_2329_cov_19.612709 | 2365 | 4.5E-148 | | |  | | | | |
|  | 1-acylglycerol-3-phosphate o-acyltransferase 6 (lysophosphatidic acid zeta) | | NODE_21982_length_2235_cov_15.554810 | 2271 | 0.0 | | |  | | | | |
|  | Novel protein vertebrate zinc finger protein 261 | | NODE_21986_length_844_cov_7.812796 | 880 | 3.3E-13 | | |  | | | | |
|  | platelet-activating factor acetylhydrolase ib subunit beta | | NODE_22467_length_1034_cov_17.404255 | 1070 | 1.8E-117 | | |  | | | | |
|  | Fk506 binding protein | | NODE_22510_length_114_cov_1113.061401 | 150 | 1.4E-24 | | |  | | | | |
|  | Golgi associated pdz and coiled-coil motif containing | | NODE_22588_length_1633_cov_18.962645 | 1669 | 1.9E-179 | | |  | | | | |
|  | Isoform cra_a | | NODE_22654_length_489_cov_12.212679 | 525 | 1.5E-103 | | |  | | | | |
|  | Beta- -galactosyltransferase | | NODE_22691_length_917_cov_44.261723 | 953 | 4.0E-164 | | |  | | | | |
|  | c-x-c chemokine receptor type 4 | | NODE_22739_length_121_cov_1953.669434 | 157 | 1.9E-28 | | |  | | | | |
|  | Tudor domain containing 9 | | NODE_22950_length_4343_cov_16.561132 | 4379 | 0.0 | | |  | | | | |
|  | c-src c terminus | | NODE_22954_length_950_cov_5.469474 | 986 | 1.6E-28 | | |  | | | | |
|  | Novel protein vertebrate family with sequence similarity member b | | NODE_23284_length_2125_cov_12.305882 | 2161 | 0.0 | | |  | | | | |
|  | Lim domain kinase 2 | | NODE_23441_length_3626_cov_20.996414 | 3662 | 0.0 | | |  | | | | |
|  | mbd2 protein | | NODE_23870_length_1530_cov_39.425491 | 1566 | 3.2E-163 | | |  | | | | |
|  | Bone morphogenetic protein 16 | | NODE_23915_length_2868_cov_6.677127 | 2904 | 0.0 | | |  | | | | |
|  | Cytochrome family subfamily polypeptide 1 | | NODE_114970_length_1702_cov_27.374853 | 1738 | 0.0 | | |  | | | | |
|  | Phosphatidic acid phosphatase type isoform cra_a | | NODE_24049_length_194_cov_20.628866 | 230 | 1.6E-44 | | |  | | | | |
|  | Chromodomain y-like protein isoform d | | NODE_24577_length_1453_cov_12.121129 | 1489 | 0.0 | | |  | | | | |
|  | Relaxin insulin-like family peptide receptor 2 | | NODE_24942_length_598_cov_5.175585 | 634 | 6.1E-131 | | |  | | | | |
|  | Spindle assembly abnormal protein 6 homolog | | NODE_75360_length_1624_cov_25.039410 | 1660 | 0.0 | | |  | | | | |
|  | Feminization 1 homolog b (elegans) | | NODE_26247_length_1467_cov_7.378323 | 1503 | 0.0 | | |  | | | | |
|  | cug triplet RNA binding protein 1 | | NODE_26488_length_844_cov_36.835308 | 880 | 1.0E-167 | | |  | | | | |
|  | Adenosine deaminase domain containing 1 (testis specific) | | NODE_26889_length_1744_cov_15.080276 | 1780 | 0.0 | | |  | | | | |
|  | Transcription factor 21 | | NODE_27022_length_359_cov_9.370474 | 395 | 5.0E-57 | | |  | | | | |
|  | Galactose-1-phosphate uridylyltransferase isoform 1 | | NODE_27033_length_1187_cov_6.138164 | 1223 | 0.0 | | |  | | | | |
|  | TATA- box-binding protein 1 | | NODE_27549_length_1411_cov_10.302622 | 1447 | 3.7E-122 | | |  | | | | |
|  | Groucho-related gene 3 protein | | NODE_27829_length_863_cov_18.682503 | 899 | 9.3E-69 | | |  | | | | |
|  | Activin receptor type-2a-like | | NODE_28159_length_1060_cov_5.473585 | 1096 | 2.8E-63 | | |  | | | | |
|  | Transforming growth beta receptor 1 | | NODE_28631_length_1292_cov_13.009288 | 1328 | 0.0 | | |  | | | | |
|  | Isoform cra_b | | NODE_29304_length_1006_cov_10.224652 | 1042 | 8.4E-15 | | |  | | | | |
|  | Dead end | | NODE_30331_length_1287_cov_15.968143 | 1323 | 0.0 | | |  | | | | |
|  | Novel protein tyrosine kinase | | NODE_32461_length_2325_cov_5.881290 | 2361 | 0.0 | | |  | | | | |
|  | fg repeats 1 | | NODE_32876_length_899_cov_6.711902 | 935 | 1.5E-163 | | |  | | | | |
|  | SOX8 protein | | NODE_33330_length_1062_cov_6.184557 | 1098 | 6.3E-16 | | |  | | | | |
|  | Sperm associated antigen 6 | | NODE_34440_length_442_cov_5.289593 | 478 | 1.2E-73 | | |  | | | | |
|  | Secretory carrier membrane protein isoform cra_b | | NODE_34738_length_832_cov_7.014423 | 868 | 5.2E-115 | | |  | | | | |
|  | Transcription factor ap-4 | | NODE_34896_length_895_cov_6.167598 | 931 | 9.3E-126 | | |  | | | | |
|  | Homologous to the e6-ap carboxyl terminus domain and rcc1 -like domain 2 | | NODE_35136_length_178_cov_5.353932 | 214 | 2.2E-39 | | |  | | | | |
|  | V-ros ur2 sarcoma virus oncogene homolog 1 isoform cra_d | | NODE_36824_length_375_cov_7.250667 | 411 | 6.8E-46 | | |  | | | | |
|  | Breast cancer susceptibility protein 2 | | NODE_37083_length_797_cov_6.342535 | 833 | 9.7E-112 | | |  | | | | |
|  | Ribosomal protein l39 | | NODE_37098_length_103_cov_3313.213623 | 139 | 1.6E-20 | | |  | | | | |
|  | mgc53561 protein | | NODE_37150_length_181_cov_5.287293 | 217 | 3.5E-35 | | |  | | | | |
|  | Membrane-type matrix metalloproteinase 1 alpha | | NODE_37379_length_2924_cov_15.335841 | 2960 | 0.0 | | |  | | | | |
|  | b chain crystal structure of the pwwp domain of human dna (cytosine- 5-)-methyltransferase 3 alpha | | NODE_38684_length_400_cov_5.425000 | 436 | 8.2E-83 | | |  | | | | |
|  | High mobility group protein b2-like isoform 2 | | NODE_40443_length_356_cov_915.210693 | 392 | 1.2E-58 | | |  | | | | |
|  | Novel protein (zgc:56016) | | NODE_41580_length_1302_cov_13.327188 | 1338 | 0.0 | | |  | | | | |
|  | Translation initiation factor eif-2b subunit beta | | NODE_41898_length_1182_cov_29.227581 | 1218 | 0.0 | | |  | | | | |
|  | Fk506 binding protein 4 | | NODE_41917_length_2131_cov_62.588455 | 2167 | 0.0 | | |  | | | | |
|  | Chemokine ligand 12 | | NODE_42382_length_82_cov_1257.487793 | 118 | 2.5E-21 | | |  | | | | |
|  | Solute carrier family 29 (nucleoside transporters) member isoform cra_a | | NODE_42846_length_1663_cov_21.223091 | 1699 | 0.0 | | |  | | | | |
|  | Tia1 cytotoxic granule-associated RNA binding 1 | | NODE_43404_length_1008_cov_66.266869 | 1044 | 2.8E-163 | | |  | | | | |
|  | cAMP responsive element binding protein 1 | | NODE_50504_length_1609_cov_5.231199 | 1645 | 6.5E-165 | | |  | | | | |
|  | cugbp elav-like family member 1 short | | NODE_43876_length_587_cov_31.385008 | 623 | 5.4E-64 | | |  | | | | |
|  | 15 kda selenoprotein | | NODE_44277_length_413_cov_88.610168 | 449 | 1.3E-76 | | |  | | | | |
|  | HIV-1 REV binding protein | | NODE_44355_length_1059_cov_31.671389 | 1095 | 4.5E-122 | | |  | | | | |
|  | Baculoviral iap repeat-containing 2 | | NODE_44613_length_239_cov_123.527199 | 275 | 1.8E-29 | | |  | | | | |
|  | daz-associated protein 1 isoform a | | NODE_44702_length_514_cov_20.264591 | 550 | 1.9E-115 | | |  | | | | |
|  | Excision repair cross-complementing rodent repair complementation group 1 | | NODE_45742_length_1016_cov_28.907480 | 1052 | 0.0 | | |  | | | | |
|  | cd81 molecule | | NODE_46825_length_1088_cov_34.041359 | 1124 | 4.3E-95 | | |  | | | | |
|  | Spindle assembly 6 homolog | | NODE_47426_length_420_cov_23.423809 | 456 | 4.1E-32 | | |  | | | | |
|  | Notch gene homolog 1 | | NODE_47455_length_398_cov_6.894473 | 434 | 9.5E-36 | | |  | | | | |
|  | RNA binding homolog 1 | | NODE_47540_length_2590_cov_24.793051 | 2626 | 0.0 | | |  | | | | |
|  | Novel protein (zgc:110560) | | NODE_48115_length_1336_cov_22.773203 | 1372 | 2.0E-177 | | |  | | | | |
|  | Poliovirus receptor-related 1 | | NODE_48314_length_217_cov_5.119816 | 253 | 3.3E-49 | | |  | | | | |
|  | Platelet-activating factor isoform gamma subunit | | NODE_48767_length_2022_cov_29.719585 | 2058 | 5.4E-142 | | |  | | | | |
|  | Pre-b-cell leukemia transcription factor 4 | | NODE_48823_length_325_cov_10.947692 | 361 | 6.3E-67 | | |  | | | | |
|  | cAMP-responsive element modulator | | NODE_50144_length_615_cov_58.730080 | 651 | 4.1E-13 | | |  | | | | |
|  | Proto-oncogene tyrosine-protein kinase ros | | NODE_53388_length_359_cov_5.267409 | 395 | 3.6E-59 | | |  | | | | |
|  | Transforming growth factor-beta receptor type i b | | NODE_53628_length_314_cov_11.136943 | 350 | 2.7E-34 | | |  | | | | |
|  | mad homolog 5 | | NODE_54948_length_1260_cov_9.707936 | 1296 | 1.7E-138 | | |  | | | | |
|  | cdyl protein | | NODE_54959_length_809_cov_9.002472 | 845 | 1.0E-54 | | |  | | | | |
|  | Gamma-glutamyltransferase isoform cra_a | | NODE_57613_length_88_cov_8.659091 | 124 | 9.9E-14 | | |  | | | | |
|  | Transforming growth beta 3 | | NODE_57843_length_657_cov_8.689498 | 693 | 1.2E-136 | | |  | | | | |
|  | Bucky ball | | NODE_60710_length_219_cov_445.566223 | 255 | 4.5E-29 | | |  | | | | |
|  | TATA box binding protein | | NODE_61822_length_1119_cov_83.555855 | 1155 | 2.0E-141 | | |  | | | | |
|  | Nucleolysin tiar | | NODE_63166_length_83_cov_56.518074 | 119 | 7.0E-18 | | |  | | | | |
|  | Microtubule-associated protein isoform cra_d | | NODE_65607_length_604_cov_16.367550 | 640 | 3.8E-97 | | |  | | | | |
|  | Ankyrin sam and basic leucine zipper domain containing 1 | | NODE_66521_length_1381_cov_14.863867 | 1417 | 0.0 | | |  | | | | |
|  | Doublesex and mab-3 related transcription factor 2a | | NODE_66597_length_610_cov_6.293443 | 646 | 4.8E-71 | | |  | | | | |
|  | wdr33 protein | | NODE_66813_length_610_cov_42.649181 | 646 | 2.1E-153 | | |  | | | | |
|  | Imp2 inner mitochondrial membrane peptidase-like | | NODE_67443_length_407_cov_5.813268 | 443 | 9.3E-32 | | |  | | | | |
|  | Barrier-to-autointegration factor | | NODE_67570_length_199_cov_21.643215 | 235 | 1.7E-21 | | |  | | | | |
|  | Novel protein vertebrate neutralized homolog | | NODE_70204_length_693_cov_5.627706 | 729 | 2.4E-130 | | |  | | | | |
|  | High-mobility group box 2 | | NODE_70605_length_138_cov_1285.413086 | 174 | 6.1E-30 | | |  | | | | |
|  | Vascular endothelial growth factor a short | | NODE_70720_length_312_cov_5.753205 | 348 | 2.0E-15 | | |  | | | | |
|  | DNA methyltransferase 3a | | NODE_70776_length_296_cov_5.358108 | 332 | 1.7E-44 | | |  | | | | |
|  | a chain crystal structure of transforming growth factor-beta2 | | NODE_71859_length_310_cov_5.632258 | 346 | 4.2E-66 | | |  | | | | |
|  | Vascular endothelial growth factor isoform cra_g | | NODE_72073_length_569_cov_5.019332 | 605 | 2.0E-29 | | |  | | | | |
|  | Integrin beta1 subunit-like protein 3 | | NODE_75099_length_120_cov_21.491667 | 156 | 9.7E-19 | | |  | | | | |
|  | Vascular endothelial growth factor aa | | NODE_79814_length_241_cov_7.419087 | 277 | 1.1E-22 | | |  | | | | |
|  | Protein inhibitor of activated stat-1 | | NODE_80749_length_539_cov_5.109462 | 575 | 6.1E-106 | | |  | | | | |
|  | Transforming growth factor beta 3 | | NODE_44319_length_514_cov_12.768482 | 550 | 4.1E-20 | | |  | | | | |
|  | DNA methyltransferase | | NODE_84206_length_236_cov_5.711864 | 272 | 1.4E-49 | | |  | | | | |
|  | DNA(cytosine-5-)-methyltransferase 8 | | NODE_89029_length_1422_cov_7.310127 | 1458 | 0.0 | | |  | | | | |
|  | b chain crystal structure of the complex between the egfr kinase domain and a mig6 peptide | | NODE_94720_length_124_cov_8.209678 | 160 | 4.7E-27 | | |  | | | | |
|  | Euchromatic histone lysine n-methyltransferase 2 | | NODE_100800_length_80_cov_34.537498 | 116 | 1.5E-18 | | |  | | | | |
|  | mgc131363 protein | | NODE_101462_length_188_cov_14.643617 | 224 | 2.9E-42 | | |  | | | | |
|  | Adenosine deaminase | | NODE_102138_length_980_cov_45.965305 | 1016 | 1.2E-32 | | |  | | | | |
|  | af487944_1 deltan p63 alpha | | NODE_109194_length_86_cov_5.151163 | 122 | 4.6E-19 | | |  | | | | |
|  | a chain crystal structure of histone lysine methyltransferase g9a with an inhibitor | | NODE_115480_length_729_cov_30.379972 | 765 | 7.3E-99 | | |  | | | | |
|  | V-src sarcoma (schmidt-ruppin a-2) viral oncogene homolog | | NODE_117886_length_131_cov_5.145038 | 167 | 2.4E-21 | | |  | | | | |
|  | Pre-b-cell leukemia transcription factor 1 isoform 2 | | NODE_138683_length_596_cov_16.835571 | 632 | 1.8E-78 | | |  | | | | |
| ***Hormone and Receptor binding related proteins*** | | | | | |  | | | |  |  |  |
|  | Adrenomedullin 1 | | NODE_1440_length_643_cov_22.214619 | 679 | 1.0E-93 | |  | | | | | |
|  | Adrenomedullin 5 | | NODE_71754_length_546_cov_6.543956 | 582 | 8.6E-18 | |  | | | | | |
|  | Thyroglobulin short | | NODE_1591_length_3619_cov_20.442112 | 3655 | 0.0 | |  | | | | | |
|  | b-type natriuretic peptide | | NODE_12422_length_694_cov_36.087894 | 730 | 4.9E-37 | |  | | | | | |
|  | Relaxin 3 | | NODE_24041_length_634_cov_8.861198 | 670 | 1.6E-32 | |  | | | | | |
|  | Anti-mullerian hormone | | NODE_35787_length_1873_cov_17.813667 | 1909 | 0.0 | |  | | | | | |
|  | Glucose-dependent insulinotropic polypeptide | | NODE_44414_length_337_cov_6.842730 | 373 | 3.2E-50 | |  | | | | | |
|  | dvr1 protein | | NODE_840_length_1229_cov_126.214806 | 1265 | 0.0 | |  | | | | | |
|  | Tuberous sclerosis 2 | | NODE_984_length_6075_cov_14.534486 | 6111 | 0.0 | |  | | | | | |
|  | Transforming growth beta 1 | | NODE_1003_length_2008_cov_22.856075 | 2044 | 0.0 | |  | | | | | |
|  | Vasoactive intestinal polypeptide type ii precursor | | NODE_1081_length_734_cov_6.846049 | 770 | 3.3E-93 | |  | | | | | |
|  | b beta polypeptide | | NODE_1247_length_344_cov_773.258728 | 380 | 6.3E-69 | |  | | | | | |
|  | Intelectin 2 | | NODE_1421_length_304_cov_107.273026 | 340 | 8.0E-22 | |  | | | | | |
|  | Fibrinogen alpha chain | | NODE_1458_length_721_cov_605.546448 | 757 | 4.4E-97 | |  | | | | | |
|  | si:ch211- protein | | NODE_1572_length_166_cov_396.228912 | 202 | 1.7E-31 | |  | | | | | |
|  | Protein phosphatase 2 (formerly 2a) regulatory subunit beta isoform | | NODE_1844_length_2188_cov_55.264168 | 2224 | 0.0 | |  | | | | | |
|  | Tenascin-C | | NODE_1873_length_1112_cov_26.371403 | 1148 | 6.9E-118 | |  | | | | | |
|  | 3-phosphoinositide-dependent protein kinase 1 | | NODE_2005_length_1163_cov_28.089424 | 1199 | 0.0 | |  | | | | | |
|  | Protein kinase beta like | | NODE_2253_length_2626_cov_46.264660 | 2662 | 0.0 | |  | | | | | |
|  | Docking protein 1b | | NODE_2524_length_2516_cov_28.446741 | 2552 | 0.0 | |  | | | | | |
|  | Talin 1 | | NODE_2625_length_5717_cov_90.900124 | 5753 | 0.0 | |  | | | | | |
|  | Sorbin and sh3 domain containing 1 | | NODE_2740_length_2714_cov_43.671333 | 2750 | 1.3E-66 | |  | | | | | |
|  | b chain crystal structure of the tandem phosphatase domains of rptp lar | | NODE_2885_length_804_cov_40.038559 | 840 | 1.3E-116 | |  | | | | | |
|  | Adenosine receptor | | NODE_2891_length_2502_cov_9.877298 | 2538 | 0.0 | |  | | | | | |
|  | Nuclear receptor coactivator 2 short | | NODE_2945_length_1550_cov_19.825806 | 1586 | 0.0 | |  | | | | | |
|  | Mediator complex subunit 12 | | NODE_3006_length_5676_cov_29.883017 | 5712 | 0.0 | |  | | | | | |
|  | Intelectin-1a precursor | | NODE_3042_length_318_cov_65.811317 | 354 | 5.9E-26 | |  | | | | | |
|  | b chain crystal structure of the moesin ferm domain complex | | NODE_3065_length_97_cov_327.082489 | 133 | 8.1E-23 | |  | | | | | |
|  | af130460_1 trp4-associated protein tap1b | | NODE_3224_length_2162_cov_25.283997 | 2198 | 0.0 | |  | | | | | |
|  | Cell proliferation-inducing protein 23 | | NODE_3251_length_611_cov_18.422258 | 647 | 1.9E-127 | |  | | | | | |
|  | Insulin-like growth factor 2 (somatomedin a) | | NODE_3252_length_1163_cov_9.612210 | 1199 | 1.7E-56 | |  | | | | | |
|  | Calmodulin | | NODE_3320_length_641_cov_212.145081 | 677 | 1.3E-93 | |  | | | | | |
|  | Riken cDNA2010204n08 gene | | NODE_3592_length_118_cov_1725.805054 | 154 | 4.5E-17 | |  | | | | | |
|  | Zgc:162290 protein | | NODE_3734_length_3068_cov_27.323338 | 3104 | 0.0 | |  | | | | | |
|  | Zgc:56306 | | NODE_3747_length_2741_cov_39.539585 | 2777 | 0.0 | |  | | | | | |
|  | Zgc:173915 protein | | NODE_80259_length_189_cov_5.894180 | 225 | 1.1E-31 | |  | | | | | |
|  | Mothers against decapentaplegic homolog 3 isoform 5 | | NODE_4663_length_2313_cov_16.620407 | 2349 | 4.9E-60 | |  | | | | | |
|  | Insulin-like growth factor ii | | NODE_4727_length_3245_cov_19.693066 | 3281 | 2.8E-115 | |  | | | | | |
|  | Laminin alpha 4 | | NODE_22762_length_4438_cov_9.737269 | 4474 | 0.0 | |  | | | | | |
|  | Docking protein isoform cra_a | | NODE_5041_length_2016_cov_15.412699 | 2052 | 1.4E-179 | |  | | | | | |
|  | Fibroblast growth factor receptor substrate 2 | | NODE_5179_length_1852_cov_16.860151 | 1888 | 0.0 | |  | | | | | |
|  | Protein phosphatase 2 (formerly 2a) regulatory subunit beta isoform | | NODE_5199_length_2864_cov_147.513962 | 2900 | 0.0 | |  | | | | | |
|  | c-c motif chemokine 25 precursor | | NODE_5256_length_600_cov_245.503326 | 636 | 3.5E-29 | |  | | | | | |
|  | Novel protein similart to vertebrate small inducible cytokine subfamily member 1 (endothelial monocyte-activating) | | NODE_5755_length_1126_cov_37.409412 | 1162 | 2.0E-147 | |  | | | | | |
|  | Zgc:55764 protein | | NODE_5820_length_344_cov_30.735466 | 380 | 5.7E-81 | |  | | | | | |
|  | Dipeptidyl peptidase iv | | NODE_6041_length_2528_cov_71.967560 | 2564 | 0.0 | |  | | | | | |
|  | Proteasome ( macropain) 26s | | NODE_6291_length_462_cov_87.235931 | 498 | 2.2E-85 | |  | | | | | |
|  | Thyroid hormone receptor associated protein isoform cra_b | | NODE_6886_length_4064_cov_39.097195 | 4100 | 0.0 | |  | | | | | |
|  | Insulin receptor substrate 1 | | NODE_6925_length_3276_cov_15.503358 | 3312 | 0.0 | |  | | | | | |
|  | Phospholipase group ib | | NODE_7180_length_187_cov_255.780746 | 223 | 2.3E-33 | |  | | | | | |
|  | Cell division protein kinase 7 | | NODE_7228_length_1206_cov_44.333332 | 1242 | 0.0 | |  | | | | | |
|  | Midkine-related growth factor mdk2 | | NODE_7460_length_667_cov_30.001499 | 703 | 2.1E-66 | |  | | | | | |
|  | Catenin beta-1 | | NODE_7669_length_2463_cov_37.782784 | 2499 | 0.0 | |  | | | | | |
|  | tnf superfamily member 14 | | NODE_7680_length_1465_cov_8.545392 | 1501 | 3.2E-29 | |  | | | | | |
|  | Janus kinase 1 | | NODE_7780_length_5774_cov_42.420677 | 5810 | 0.0 | |  | | | | | |
|  | Chemokine ccl-c20d | | NODE_7788_length_265_cov_83.120758 | 301 | 2.7E-44 | |  | | | | | |
|  | Protein tyrosine kinase jak2b | | NODE_7848_length_3048_cov_24.873360 | 3084 | 0.0 | |  | | | | | |
|  | Growth and differentiation factor 9 | | NODE_8028_length_1545_cov_61.159870 | 1581 | 0.0 | |  | | | | | |
|  | Glia maturation beta | | NODE_8030_length_1922_cov_22.044744 | 1958 | 1.1E-92 | |  | | | | | |
|  | Angiopoietin-like 7 | | NODE_8037_length_1286_cov_137.910568 | 1322 | 0.0 | |  | | | | | |
|  | shc (src homology 2 domain containing) transforming protein 1 | | NODE_8118_length_2663_cov_53.577919 | 2699 | 0.0 | |  | | | | | |
|  | Vascular endothelial growth factor aa | | NODE_8122_length_2341_cov_21.438702 | 2377 | 3.0E-72 | |  | | | | | |
|  | Novel protein (zgc:92871) | | NODE_8557_length_1189_cov_39.865433 | 1225 | 8.3E-90 | |  | | | | | |
|  | Nicotinamide phosphoribosyltransferase | | NODE_8575_length_2148_cov_27.011639 | 2184 | 0.0 | |  | | | | | |
|  | Rabaptin-5 | | NODE_8664_length_1714_cov_23.577013 | 1750 | 1.7E-58 | |  | | | | | |
|  | Protein kinase c beta type short | | NODE_8889_length_4284_cov_22.119047 | 4320 | 0.0 | |  | | | | | |
|  | Laminin alpha 5 | | NODE_9077_length_5037_cov_14.524320 | 5073 | 0.0 | |  | | | | | |
|  | gipc2 protein | | NODE_9194_length_1193_cov_72.201172 | 1229 | 0.0 | |  | | | | | |
|  | Coagulation factor ii | | NODE_9407_length_702_cov_281.987183 | 738 | 2.5E-169 | |  | | | | | |
|  | Chemokine ccl-c5a | | NODE_9892_length_606_cov_35.334984 | 642 | 1.0E-37 | |  | | | | | |
|  | Msn protein | | NODE_10106_length_1049_cov_238.388947 | 1085 | 8.1E-163 | |  | | | | | |
|  | Mannose-binding lectin isoform 2 | | NODE_10194_length_137_cov_43.430656 | 173 | 1.9E-30 | |  | | | | | |
|  | Rabaptin-5 | | NODE_10333_length_2241_cov_20.108879 | 2277 | 0.0 | |  | | | | | |
|  | Cadherin 5 | | NODE_10379_length_1952_cov_31.495390 | 1988 | 0.0 | |  | | | | | |
|  | Mediator of rna polymerase ii transcription subunit 30 | | NODE_10436_length_1122_cov_11.040998 | 1158 | 9.0E-94 | |  | | | | | |
|  | Rab3 GTPase activating protein subunit 2 (non-catalytic) | | NODE_10442_length_3438_cov_14.392961 | 3474 | 0.0 | |  | | | | | |
|  | Talin-2 | | NODE_10563_length_4436_cov_10.835212 | 4472 | 0.0 | |  | | | | | |
|  | Cotamer alpha | | NODE_10791_length_3434_cov_77.394585 | 3470 | 0.0 | |  | | | | | |
|  | Protein inhibitor of activated 2 | | NODE_11310_length_1554_cov_19.981339 | 1590 | 0.0 | |  | | | | | |
|  | Estrogen receptor binding site associated antigen 9 variant 1 | | NODE_11346_length_1357_cov_39.667648 | 1393 | 2.4E-96 | |  | | | | | |
|  | Fibroblast growth factor 14 | | NODE_11438_length_2011_cov_28.071606 | 2047 | 1.2E-126 | |  | | | | | |
|  | b chain interconversion of human lysosomal enzyme specificities | | NODE_11496_length_751_cov_7.769640 | 787 | 4.8E-41 | |  | | | | | |
|  | Chemokine ccl-c25ab | | NODE_11732_length_403_cov_75.995041 | 439 | 2.0E-43 | |  | | | | | |
|  | zgc:136318 protein | | NODE_11834_length_570_cov_168.092987 | 606 | 4.8E-83 | |  | | | | | |
|  | Nuclear receptor co-repressor | | NODE_11934_length_1350_cov_25.988148 | 1386 | 5.5E-143 | |  | | | | | |
|  | Serine threonine kinase receptor associated protein | | NODE_12001_length_1394_cov_76.217361 | 1430 | 0.0 | |  | | | | | |
|  | Mediator complex subunit 13 | | NODE_12160_length_3504_cov_28.152111 | 3540 | 0.0 | |  | | | | | |
|  | Fk506 binding protein 1b | | NODE_12175_length_951_cov_19.435331 | 987 | 3.0E-72 | |  | | | | | |
|  | Nuclear receptor binding set domain protein 1-like | | NODE_12271_length_2919_cov_5.681398 | 2955 | 0.0 | |  | | | | | |
|  | ptp1b protein | | NODE_12597_length_1540_cov_82.621429 | 1576 | 0.0 | |  | | | | | |
|  | Nuclear receptor-interacting protein 1 isoform 1 | | NODE_13273_length_2049_cov_7.367496 | 2085 | 9.4E-78 | |  | | | | | |
|  | Zgc:171687 protein | | NODE_13318_length_205_cov_10.117073 | 241 | 7.5E-22 | |  | | | | | |
|  | Polycystic kidney disease 2 | | NODE_13580_length_2623_cov_12.499047 | 2659 | 0.0 | |  | | | | | |
|  | Ataxin-1 ubiquitin-like interacting protein | | NODE_13612_length_1560_cov_64.609619 | 1596 | 0.0 | |  | | | | | |
|  | smad family member 3 | | NODE_13743_length_1188_cov_19.527779 | 1224 | 0.0 | |  | | | | | |
|  | Novel protein vertebrate docking protein family | | NODE_14094_length_2856_cov_30.504202 | 2892 | 0.0 | |  | | | | | |
|  | af130459_1 trp4-associated protein tap1a | | NODE_14395_length_2056_cov_32.193581 | 2092 | 1.0E-22 | |  | | | | | |
|  | Angiopoietin-related protein 4 precursor | | NODE_14876_length_2279_cov_44.260201 | 2315 | 0.0 | |  | | | | | |
|  | 100 kda thyroid hormone receptor associated protein | | NODE_15072_length_3454_cov_17.016792 | 3490 | 0.0 | |  | | | | | |
|  | Mediator of rna polymerase ii transcription subunit 13-like ame | | NODE_15164_length_4090_cov_30.955257 | 4126 | 0.0 | |  | | | | | |
|  | Fibroblast growth factor receptor substrate 2 | | NODE_15256_length_629_cov_5.788553 | 665 | 6.2E-37 | |  | | | | | |
|  | prp6 pre-mrna splicing factor 6 homolog | | NODE_15372_length_2922_cov_20.798426 | 2958 | 0.0 | |  | | | | | |
|  | Insulin-like 5b | | NODE_15376_length_819_cov_10.401710 | 855 | 8.4E-41 | |  | | | | | |
|  | ncoa1 protein | | NODE_15441_length_2055_cov_7.779562 | 2091 | 0.0 | |  | | | | | |
|  | rab GTPase-binding effector protein 2 | | NODE_15487_length_1260_cov_25.968254 | 1296 | 0.0 | |  | | | | | |
|  | a chain crystal structure of lysine-specific demethylase1 | | NODE_15551_length_771_cov_41.868999 | 807 | 4.4E-171 | |  | | | | | |
|  | Casein kinase ii subunit beta | | NODE_15897_length_1122_cov_107.538322 | 1158 | 5.0E-147 | |  | | | | | |
|  | Endosulfine alpha | | NODE_16236_length_2950_cov_35.659660 | 2986 | 5.2E-69 | |  | | | | | |
|  | Novel protein vertebrate complement component 3 | | NODE_16330_length_512_cov_62.382812 | 548 | 8.1E-101 | |  | | | | | |
|  | Canopy 4 homolog | | NODE_16852_length_1160_cov_12.468103 | 1196 | 2.9E-121 | |  | | | | | |
|  | Hepatoma-derived growth related protein 3 | | NODE_16885_length_1287_cov_22.878012 | 1323 | 4.8E-93 | |  | | | | | |
|  | der1-like domain member 1 | | NODE_16909_length_1030_cov_77.176697 | 1066 | 3.3E-173 | |  | | | | | |
|  | Macrophage colony-stimulating factor-2 | | NODE_16914_length_1311_cov_18.552250 | 1347 | 5.0E-163 | |  | | | | | |
|  | nck1 protein | | NODE_17165_length_2579_cov_20.825127 | 2615 | 0.0 | |  | | | | | |
|  | gphn protein | | NODE_17292_length_959_cov_6.244004 | 995 | 0.0 | |  | | | | | |
|  | 3-phosphoinositide dependent protein kinase-1 | | NODE_17683_length_1752_cov_62.765984 | 1788 | 0.0 | |  | | | | | |
|  | Protein tyrosine non-receptor type 11 | | NODE_39055_length_1335_cov_47.758053 | 1371 | 0.0 | |  | | | | | |
|  | Angiopoietin-like 2b | | NODE_18222_length_1122_cov_10.341354 | 1158 | 1.3E-109 | |  | | | | | |
|  | mark1 protein | | NODE_18880_length_1997_cov_9.462694 | 2033 | 0.0 | |  | | | | | |
|  | Trafficking kinesin binding 2 | | NODE_18897_length_483_cov_22.142857 | 519 | 9.5E-32 | |  | | | | | |
|  | Retinoic acid receptor rxr-gamma-b | | NODE_18910_length_807_cov_26.296158 | 843 | 4.0E-101 | |  | | | | | |
|  | Aryl hydrocarbon receptor nuclear translocator | | NODE_19181_length_1538_cov_44.919376 | 1574 | 0.0 | |  | | | | | |
|  | Hypothetical protein RCJMB04_27h20 | | NODE_19416_length_2159_cov_8.507179 | 2195 | 0.0 | |  | | | | | |
|  | Interleukin-10 | | NODE_19435_length_460_cov_23.660870 | 496 | 5.1E-78 | |  | | | | | |
|  | cAMP-regulated phosphoprotein 19 | | NODE_20499_length_1190_cov_14.756303 | 1226 | 2.6E-57 | |  | | | | | |
|  | Mediator of RNA polymerase ii transcription subunit 17 | | NODE_20595_length_1866_cov_33.781349 | 1902 | 0.0 | |  | | | | | |
|  | psmc3 interacting protein | | NODE_20727_length_924_cov_22.384199 | 960 | 3.7E-132 | |  | | | | | |
|  | a chain crystal structures of peptide complexes of the amino- terminal sh2 domain of the syp tyrosine phosphatase | | NODE_21036_length_342_cov_28.368422 | 378 | 2.1E-23 | |  | | | | | |
|  | b chain crystal structure of the tandem phosphatase domains of rptp lar | | NODE_21518_length_573_cov_29.038395 | 609 | 3.1E-118 | |  | | | | | |
|  | Connective tissue growth factor | | NODE_22316_length_1564_cov_15.947571 | 1600 | 0.0 | |  | | | | | |
|  | Novel protein vertebrate mothers against dpp homolog 6 | | NODE_22352_length_1603_cov_5.230194 | 1639 | 0.0 | |  | | | | | |
|  | Neurexophilin 1 | | NODE_22729_length_1264_cov_5.689082 | 1300 | 1.3E-82 | |  | | | | | |
|  | Cyclin-dependent kinase 5-like isoform 2 | | NODE_22815_length_575_cov_13.093913 | 611 | 2.1E-79 | |  | | | | | |
|  | Transforming growth factor-beta receptor-associated protein 1 homolog | | NODE_23430_length_1158_cov_6.248704 | 1194 | 0.0 | |  | | | | | |
|  | Swi snf matrix actin dependent regulator of subfamily member 3 | | NODE_23457_length_1744_cov_9.740252 | 1780 | 0.0 | |  | | | | | |
|  | Platelet-derived growth factor c | | NODE_23759_length_1681_cov_15.965497 | 1717 | 0.0 | |  | | | | | |
|  | Relaxin 3 | | NODE_24041_length_634_cov_8.861198 | 670 | 1.6E-32 | |  | | | | | |
|  | Glia maturation factor gamma | | NODE_24047_length_1107_cov_42.930443 | 1143 | 1.6E-80 | |  | | | | | |
|  | Zgc:77076 | | NODE_24358_length_398_cov_10.155779 | 434 | 2.3E-52 | |  | | | | | |
|  | Mediator complex subunit 16 | | NODE_24681_length_1998_cov_7.188188 | 2034 | 0.0 | |  | | | | | |
|  | Acta1 protein | | NODE_25364_length_408_cov_11.480392 | 444 | 9.9E-42 | |  | | | | | |
|  | Bromodomain and wd repeat domain containing 2 | | NODE_25769_length_4415_cov_10.135221 | 4451 | 0.0 | |  | | | | | |
|  | Ephrin b2 | | NODE_26046_length_1198_cov_5.716194 | 1234 | 0.0 | |  | | | | | |
|  | Melanocortin-2 receptor accessory protein 2-like | | NODE_26048_length_1081_cov_14.636448 | 1117 | 2.3E-36 | |  | | | | | |
|  | Adenylate cyclase type 6 | | NODE_27409_length_2354_cov_5.779524 | 2390 | 0.0 | |  | | | | | |
|  | Zgc:174864 protein | | NODE_27739_length_264_cov_6.496212 | 300 | 8.7E-50 | |  | | | | | |
|  | Fibrinogen-like 2 | | NODE_27786_length_1296_cov_7.641975 | 1332 | 1.3E-149 | |  | | | | | |
|  | Nuclear receptor binding factor 2 | | NODE_27851_length_461_cov_9.210412 | 497 | 7.3E-67 | |  | | | | | |
|  | Bone morphogenetic protein 1a | | NODE_27966_length_3184_cov_15.309045 | 3220 | 0.0 | |  | | | | | |
|  | Interleukin-1 receptor-like protein | | NODE_28172_length_2005_cov_13.736658 | 2041 | 2.6E-142 | |  | | | | | |
|  | Stomatin -like 2 | | NODE_28229_length_182_cov_25.131868 | 218 | 4.0E-19 | |  | | | | | |
|  | Transforming growth beta receptor iii (2) | | NODE_28390_length_3210_cov_13.556698 | 3246 | 0.0 | |  | | | | | |
|  | smad specific e3 ubiquitin protein ligase 2 | | NODE_28704_length_873_cov_15.255441 | 909 | 4.9E-137 | |  | | | | | |
|  | Catenin beta-1 | | NODE_29364_length_2533_cov_53.795498 | 2569 | 0.0 | |  | | | | | |
|  | prkca-binding protein | | NODE_29469_length_2085_cov_12.277698 | 2121 | 0.0 | |  | | | | | |
|  | cd40 ligand | | NODE_29634_length_1065_cov_6.708920 | 1101 | 4.2E-75 | |  | | | | | |
|  | Novel protein human rab3 GTPase-activating protein | | NODE_29864_length_1489_cov_12.204836 | 1525 | 0.0 | |  | | | | | |
|  | Growth factor receptor-bound protein 14 | | NODE_29990_length_1150_cov_14.118261 | 1186 | 0.0 | |  | | | | | |
|  | Beta-tubulin c terminus | | NODE_30992_length_1074_cov_6.779330 | 1110 | 1.4E-57 | |  | | | | | |
|  | gipc pdz domain containing member isoform cra_b | | NODE_31053_length_430_cov_6.809302 | 466 | 2.4E-63 | |  | | | | | |
|  | Novel protein vertebrate nck adaptor protein 1 | | NODE_31248_length_1521_cov_12.380671 | 1557 | 0.0 | |  | | | | | |
|  | adm2 protein | | NODE_32094_length_1862_cov_16.972610 | 1898 | 3.9E-66 | |  | | | | | |
|  | Fgg protein | | NODE_32128_length_244_cov_724.331970 | 280 | 2.3E-41 | |  | | | | | |
|  | Glucagon a | | NODE_32260_length_676_cov_8.002958 | 712 | 6.8E-78 | |  | | | | | |
|  | c-x-c motif chemokine 14 precursor | | NODE_32390_length_1023_cov_16.409580 | 1059 | 2.3E-67 | |  | | | | | |
|  | Invariant chain-like protein 1 | | NODE_33192_length_81_cov_4845.888672 | 117 | 1.2E-11 | |  | | | | | |
|  | af144689_1 bmal1 | | NODE_33278_length_1241_cov_5.833199 | 1277 | 0.0 | |  | | | | | |
|  | Novel protein vertebrate bone morphogenetic protein | | NODE_33872_length_1049_cov_5.086749 | 1085 | 1.7E-45 | |  | | | | | |
|  | 3-keto-steroid reductase-like | | NODE_34824_length_1448_cov_6.959254 | 1484 | 2.1E-156 | |  | | | | | |
|  | Neuregulin 1 type i isoform | | NODE_35010_length_2168_cov_13.606550 | 2204 | 3.0E-25 | |  | | | | | |
|  | Nuclear receptor binding set domain protein 1 isoform cra_a | | NODE_35603_length_1353_cov_7.852920 | 1389 | 0.0 | |  | | | | | |
|  | Neuroplastin precursor | | NODE_36268_length_1219_cov_6.716981 | 1255 | 1.5E-132 | |  | | | | | |
|  | adcyap1a protein | | NODE_36524_length_1016_cov_5.743110 | 1052 | 3.6E-130 | |  | | | | | |
|  | Angiopoietin 1 | | NODE_36754_length_1923_cov_7.969839 | 1959 | 0.0 | |  | | | | | |
|  | Ras-related c3 botulinum toxin substrate 3 (rho small gtp binding protein rac3) | | NODE_36848_length_825_cov_5.423030 | 861 | 1.5E-139 | |  | | | | | |
|  | Erythropoietin-l2 flags | | NODE_37241_length_615_cov_8.967480 | 651 | 1.9E-115 | |  | | | | | |
|  | Ribonuclease like 2 | | NODE_38197_length_226_cov_8.619469 | 262 | 7.5E-22 | |  | | | | | |
|  | Invariant chain-like protein 1 | | NODE_38659_length_135_cov_3281.281494 | 171 | 6.9E-29 | |  | | | | | |
|  | Solute carrier family 39 (zinc transporter) member 1 | | NODE_38876_length_785_cov_13.284077 | 821 | 5.9E-53 | |  | | | | | |
|  | Protein tyrosine non-receptor type 11 | | NODE_39055_length_1335_cov_47.758053 | 1371 | 0.0 | |  | | | | | |
|  | ATP-binding cassette 1 | | NODE_39057_length_193_cov_5.373057 | 229 | 9.1E-39 | |  | | | | | |
|  | Tumor necrosis factor alpha | | NODE_39247_length_360_cov_5.041667 | 396 | 2.3E-29 | |  | | | | | |
|  | Gonadotropin-releasing hormone 3 | | NODE_39618_length_224_cov_5.008929 | 260 | 1.1E-29 | |  | | | | | |
|  | hbl3 protein | | NODE_39897_length_174_cov_69.327583 | 210 | 4.2E-33 | |  | | | | | |
|  | cc chemokine scya102 | | NODE_40566_length_100_cov_173.029999 | 136 | 9.5E-16 | |  | | | | | |
|  | af149802_1 pleiotrophin 1 | | NODE_42854_length_1260_cov_50.628571 | 1296 | 1.2E-71 | |  | | | | | |
|  | trak1 protein | | NODE_44221_length_278_cov_10.309353 | 314 | 1.9E-44 | |  | | | | | |
|  | Low density lipoprotein receptor-related protein associated protein 1 | | NODE_44424_length_677_cov_50.853767 | 713 | 5.9E-145 | |  | | | | | |
|  | G protein-coupled family group member c | | NODE_45627_length_1473_cov_35.397148 | 1509 | 0.0 | |  | | | | | |
|  | Triadin | | NODE_45668_length_290_cov_5.517241 | 326 | 6.3E-40 | |  | | | | | |
|  | 26s protease regulatory subunit 8 | | NODE_46782_length_733_cov_75.215553 | 769 | 1.7E-163 | |  | | | | | |
|  | wnt11-related protein | | NODE_47832_length_313_cov_5.677316 | 349 | 2.0E-55 | |  | | | | | |
|  | cc chemokine scya112 | | NODE_48432_length_242_cov_60.743801 | 278 | 8.6E-7 | |  | | | | | |
|  | Beta 2c | | NODE_49314_length_808_cov_78.909653 | 844 | 0.0 | |  | | | | | |
|  | Gamma polypeptide | | NODE_49657_length_911_cov_231.578491 | 947 | 4.9E-27 | |  | | | | | |
|  | Riken cDNA 2010204n08 gene | | NODE_50127_length_139_cov_1942.597168 | 175 | 1.3E-22 | |  | | | | | |
|  | b chain crystal structure of the grb14 sh2 domain | | NODE_55726_length_661_cov_16.579426 | 697 | 3.1E-53 | |  | | | | | |
|  | frs2 protein | | NODE_57083_length_693_cov_21.406927 | 729 | 9.6E-51 | |  | | | | | |
|  | Platelet derived growth factor alpha b | | NODE_57480_length_1642_cov_8.023751 | 1678 | 7.4E-103 | |  | | | | | |
|  | Small inducible cytokine a4 | | NODE_58760_length_400_cov_7.680000 | 436 | 9.0E-36 | |  | | | | | |
|  | sacs protein | | NODE_59003_length_124_cov_5.411290 | 160 | 5.4E-17 | |  | | | | | |
|  | Natriuretic peptide precursor a | | NODE_60614_length_710_cov_226.830978 | 746 | 4.7E-58 | |  | | | | | |
|  | Interleukin 15 | | NODE_69005_length_326_cov_20.343557 | 362 | 1.6E-14 | |  | | | | | |
|  | Oncostatin-m-specific receptor subunit beta | | NODE_61839_length_1656_cov_12.043478 | 1692 | 4.7E-12 | |  | | | | | |
|  | Interleukin-1 beta 2 | | NODE_62274_length_174_cov_103.695404 | 210 | 6.0E-36 | |  | | | | | |
|  | a chain fragment based discovery of jak-2 inhibitors | | NODE_62887_length_607_cov_113.678749 | 643 | 6.5E-140 | |  | | | | | |
|  | 106 kda o- c transferase-interacting protein isoform cra_a | | NODE_63459_length_1102_cov_19.018148 | 1138 | 1.6E-150 | |  | | | | | |
|  | Zgc:172115 protein | | NODE_64509_length_820_cov_28.073172 | 856 | 5.8E-46 | |  | | | | | |
|  | Interleukin-8 | | NODE_65176_length_483_cov_14.906833 | 519 | 6.8E-60 | |  | | | | | |
|  | vegfab210 precursor | | NODE_66234_length_993_cov_44.963745 | 1029 | 4.0E-29 | |  | | | | | |
|  | iclp2 protein | | NODE_69208_length_129_cov_2494.209229 | 165 | 2.9E-25 | |  | | | | | |
|  | Galanin prepropeptide | | NODE_72203_length_298_cov_8.983221 | 334 | 4.4E-53 | |  | | | | | |
|  | Phosphoinositide-3- regulatory subunit 1 (p85 alpha) | | NODE_73210_length_474_cov_557.535889 | 510 | 2.6E-116 | |  | | | | | |
|  | a chain ras-GTPase-activating domain of human p120gap | | NODE_79313_length_842_cov_7.570071 | 878 | 3.7E-144 | |  | | | | | |
|  | a chain solution structure of the c-terminal sh2 domain of the p85alpha regulatory subunit of phosphoinositide 3- minimized average structure | | NODE_83053_length_504_cov_496.611115 | 540 | 3.4E-32 | |  | | | | | |
|  | Angiopoietin-like 3 | | NODE_83708_length_2666_cov_22.277569 | 2702 | 0.0 | |  | | | | | |
|  | Novel protein vertebrate glutamate receptor interacting protein 1 | | NODE_84141_length_357_cov_7.417367 | 393 | 3.5E-68 | |  | | | | | |
|  | c-c motif chemokine 28 precursor | | NODE_89061_length_435_cov_8.455173 | 471 | 2.2E-9 | |  | | | | | |
|  | gprc5c protein | | NODE_89933_length_124_cov_21.120968 | 160 | 2.6E-18 | |  | | | | | |
|  | Chemokine (c-c motif) ligand 14 | | NODE_92624_length_151_cov_45.298012 | 187 | 5.1E-15 | |  | | | | | |
|  | Interleukin 8 | | NODE_102581_length_580_cov_16.525862 | 616 | 2.4E-36 | |  | | | | | |
|  | loc100135092 protein | | NODE_114281_length_74_cov_5.270270 | 110 | 2.6E-14 | |  | | | | | |
|  | Fibroblast growth factor 2 | | NODE_121650_length_501_cov_7.788423 | 537 | 1.8E-56 | |  | | | | | |
|  | |  | | | | | | | | | | |
| ***Receptor activity related proteins*** | | | | | |  | | | | | | |
|  | Transportin 3 | | NODE_4_length_3854_cov_54.668655 | 3890 | 0.0 | |  | | | | | |
|  | Neurogenic locus notch homolog protein 1 | | NODE_316_length_1750_cov_8.647429 | 1786 | 0.0 | |  | | | | | |
|  | Aryl hydrocarbon receptor 2 betaelongation factor 1 gamma | | NODE_437_length_4546_cov_76.535194 | 4582 | 0.0 | |  | | | | | |
|  | Beta 4 integrinelongation factor 1 beta 2 | | NODE_485_length_4492_cov_69.673195 | 4528 | 0.0 | |  | | | | | |
|  | Ribosomal protein sa | | NODE_731_length_165_cov_602.042419 | 201 | 4.9E-20 | |  | | | | | |
|  | Sodium-dependent phosphate transporter 1-b | | NODE_855_length_667_cov_109.964020 | 703 | 1.9E-125 | |  | | | | | |
|  | Low-density lipoprotein receptor-related protein 1 | | NODE_968_length_5051_cov_37.337559 | 5087 | 0.0 | |  | | | | | |
|  | Integrin beta-2 precursor | | NODE_1057_length_956_cov_157.246857 | 992 | 0.0 | |  | | | | | |
|  | Coxsackie virus and adenovirus receptor | | NODE_1143_length_2732_cov_29.488653 | 2768 | 0.0 | |  | | | | | |
|  | Nuclear receptor subfamily 2 group b member 3-a | | NODE_1161_length_1736_cov_52.532833 | 1772 | 0.0 | |  | | | | | |
|  | Nuclear receptor subfamily group member 2b | | NODE_1257_length_2227_cov_40.188595 | 2263 | 0.0 | |  | | | | | |
|  | Lysyl oxidase-like 2a | | NODE_1363_length_390_cov_20.897436 | 426 | 1.4E-93 | |  | | | | | |
|  | Semaphorin 3fa | | NODE_1394_length_1617_cov_5.946197 | 1653 | 3.2E-157 | |  | | | | | |
|  | Somatostatin receptor type 5 | | NODE_1478_length_2256_cov_12.318262 | 2292 | 0.0 | |  | | | | | |
|  | Vitellogenin receptor | | NODE_1516_length_2061_cov_54.388645 | 2097 | 0.0 | |  | | | | | |
|  | Tyrosine-protein kinase receptor ufo-like | | NODE_1667_length_6085_cov_20.343468 | 6121 | 0.0 | |  | | | | | |
|  | Novel protein | | NODE_1703_length_283_cov_130.968201 | 319 | 6.2E-15 | |  | | | | | |
|  | Interleukin 17 receptor isoform cra_c | | NODE_1735_length_2130_cov_25.591549 | 2166 | 0.0 | |  | | | | | |
|  | Insulin receptor b | | NODE_1879_length_4415_cov_29.529106 | 4451 | 0.0 | |  | | | | | |
|  | Protein tyrosine non-receptor type 6 | | NODE_1977_length_1118_cov_172.934708 | 1154 | 0.0 | |  | | | | | |
|  | Interleukin-20 receptor alpha | | NODE_1989_length_494_cov_24.993927 | 530 | 2.0E-54 | |  | | | | | |
|  | Renin receptor precursor | | NODE_2029_length_1067_cov_57.963448 | 1103 | 0.0 | |  | | | | | |
|  | Interleukin 2 receptor gamma | | NODE_2032_length_202_cov_18.386139 | 238 | 7.5E-19 | |  | | | | | |
|  | af250042_1 ovarian tnf receptor | | NODE_2087_length_1627_cov_20.993238 | 1663 | 7.5E-107 | |  | | | | | |
|  | Nuclear receptor subfamily group member 1 | | NODE_2123_length_2416_cov_144.900253 | 2452 | 0.0 | |  | | | | | |
|  | Polyribonucleotide 5 -hydroxyl-kinase clp1 | | NODE_2143_length_1819_cov_29.741066 | 1855 | 0.0 | |  | | | | | |
|  | Novel protein vertebrate chemokine (c-c motif) receptor 9 | | NODE_2166_length_1614_cov_370.965912 | 1650 | 0.0 | |  | | | | | |
|  | tpa: interleukin 2 receptor gamma | | NODE_2204_length_187_cov_21.021391 | 223 | 7.7E-26 | |  | | | | | |
|  | v-erb-b2 erythroblastic leukemia viral oncogene-like 3 | | NODE_2364_length_5451_cov_17.099064 | 5487 | 0.0 | |  | | | | | |
|  | Serine protease hepsin | | NODE_2414_length_2070_cov_11.469082 | 2106 | 0.0 | |  | | | | | |
|  | tpa: interleukin 12 receptor beta | | NODE_2431_length_2757_cov_17.457745 | 2793 | 0.0 | |  | | | | | |
|  | Semaphorin 3ga | | NODE_2512_length_2485_cov_27.310261 | 2521 | 0.0 | |  | | | | | |
|  | Novel protein vertebrate protein tyrosine non-receptor type 23 | | NODE_2617_length_3614_cov_40.216934 | 3650 | 0.0 | |  | | | | | |
|  | Peroxisome proliferator activated receptor gamma | | NODE_2630_length_1754_cov_24.132269 | 1790 | 0.0 | |  | | | | | |
|  | tpa: prolactin | | NODE_2774_length_883_cov_53.317101 | 919 | 6.6E-135 | |  | | | | | |
|  | tnfrsf11a protein | | NODE_2785_length_594_cov_8.624579 | 630 | 3.2E-101 | |  | | | | | |
|  | tpa: interleukin 21 receptor | | NODE_2916_length_1509_cov_22.181578 | 1545 | 8.0E-173 | |  | | | | | |
|  | Prostaglandin e2 subtype ep4 receptor | | NODE_2982_length_1655_cov_116.838066 | 1691 | 0.0 | |  | | | | | |
|  | Alanyl aminopeptidase (aminopeptidase aminopeptidase microsomal p150) isoform cra_a | | NODE_3020_length_3066_cov_12.092302 | 3102 | 0.0 | |  | | | | | |
|  | Solute carrier family member 1 | | NODE_3104_length_500_cov_747.453979 | 536 | 1.4E-24 | |  | | | | | |
|  | Plexin a1 | | NODE_3187_length_6688_cov_19.275419 | 6724 | 0.0 | |  | | | | | |
|  | Retinoic acid alpha a | | NODE_3253_length_2259_cov_50.057106 | 2295 | 0.0 | |  | | | | | |
|  | Peroxisome proliferator-activated receptor delta b | | NODE_46418_length_2326_cov_10.727429 | 2362 | 0.0 | |  | | | | | |
|  | Signal recognition particle receptor ( docking protein ) isoform cra_a | | NODE_3298_length_2181_cov_49.646034 | 2217 | 0.0 | |  | | | | | |
|  | Novel protein vertebrate natriuretic peptide receptor a guanylate cyclase a | | NODE_3334_length_1978_cov_9.627907 | 2014 | 0.0 | |  | | | | | |
|  | Novel protein vertebrate stabilin 2 | | NODE_3338_length_3988_cov_9.581745 | 4024 | 0.0 | |  | | | | | |
|  | Receptor-interacting protein 1 | | NODE_3708_length_3392_cov_10.408314 | 3428 | 0.0 | |  | | | | | |
|  | Zgc:165629 protein | | NODE_3746_length_1598_cov_20.768461 | 1634 | 0.0 | |  | | | | | |
|  | Semaphorin 3aa | | NODE_3748_length_4369_cov_17.634470 | 4405 | 0.0 | |  | | | | | |
|  | Nonspecific cytotoxic cell receptor protein 1 | | NODE_3782_length_383_cov_140.686691 | 419 | 2.7E-81 | |  | | | | | |
|  | T-cell receptor beta chain ana | | NODE_3802_length_221_cov_15.823529 | 257 | 4.1E-17 | |  | | | | | |
|  | Protein tyrosine non-receptor type 2 | | NODE_3814_length_1572_cov_46.012722 | 1608 | 0.0 | |  | | | | | |
|  | cd18 protein | | NODE_3945_length_175_cov_271.782867 | 211 | 2.5E-44 | |  | | | | | |
|  | fgfr2 protein | | NODE_4019_length_2204_cov_8.186026 | 2240 | 0.0 | |  | | | | | |
|  | grp1 (general receptor for phosphoinositides 1)-associated scaffold protein | | NODE_4067_length_643_cov_81.171074 | 679 | 1.8E-94 | |  | | | | | |
|  | Adiponectin receptor 2 | | NODE_4121_length_1681_cov_66.983345 | 1717 | 0.0 | |  | | | | | |
|  | af420018_1 alpha e integrin | | NODE_4127_length_243_cov_111.646088 | 279 | 1.8E-45 | |  | | | | | |
|  | Prostaglandin e receptor 2 (subtype ep2)-like | | NODE_4132_length_1748_cov_7.586384 | 1784 | 0.0 | |  | | | | | |
|  | Progesterone receptor membrane component 1 | | NODE_4175_length_850_cov_116.269409 | 886 | 7.7E-106 | |  | | | | | |
|  | G protein-coupled receptor isoform cra_d | | NODE_4190_length_2673_cov_31.565283 | 2709 | 0.0 | |  | | | | | |
|  | Integrin alpha v | | NODE_4215_length_4085_cov_58.173073 | 4121 | 0.0 | |  | | | | | |
|  | Immunoglobulin-like domain containing receptor 1 | | NODE_4487_length_2592_cov_14.534722 | 2628 | 0.0 | |  | | | | | |
|  | Transient receptor potential melastatin 7 | | NODE_4490_length_1164_cov_48.221649 | 1200 | 0.0 | |  | | | | | |
|  | GABA receptor associated protein | | NODE_4497_length_380_cov_174.231583 | 416 | 2.9E-83 | |  | | | | | |
|  | Platelet-derived growth factor receptor alpha short | | NODE_4575_length_2651_cov_6.897397 | 2687 | 0.0 | |  | | | | | |
|  | Low density lipo protein 6 | | NODE_4607_length_2872_cov_11.156337 | 2908 | 0.0 | |  | | | | | |
|  | Myeloid differentiation primary response gene | | NODE_4819_length_1761_cov_26.538330 | 1797 | 1.5E-179 | |  | | | | | |
|  | ephrin type-a receptor 2-like | | NODE_4829_length_3448_cov_12.906902 | 3484 | 0.0 | |  | | | | | |
|  | Ryanodine receptor domain and socs box containing 4b | | NODE_4854_length_772_cov_33.358807 | 808 | 0.0 | |  | | | | | |
|  | Transmembrane serine 3 | | NODE_4884_length_917_cov_165.958557 | 953 | 3.6E-179 | |  | | | | | |
|  | Frizzled-6 short | | NODE_4887_length_1726_cov_21.903824 | 1762 | 5.5E-38 | |  | | | | | |
|  | Zgc:55307 protein | | NODE_7183_length_1138_cov_303.343597 | 1174 | 0.0 | |  | | | | | |
|  | Vitamin d receptor a | | NODE_5123_length_2396_cov_29.707430 | 2432 | 0.0 | |  | | | | | |
|  | Novel protein vertebrate tumor necrosis factor receptor member 21 | | NODE_5242_length_1593_cov_25.491526 | 1629 | 2.0E-179 | |  | | | | | |
|  | G protein-coupled receptor kinase 5 | | NODE_5253_length_2919_cov_21.049332 | 2955 | 0.0 | |  | | | | | |
|  | Translocon-associated protein alpha | | NODE_5300_length_1094_cov_148.306213 | 1130 | 3.3E-130 | |  | | | | | |
|  | Transient receptor potential melastatin 7 | | NODE_5368_length_2422_cov_47.771675 | 2458 | 0.0 | |  | | | | | |
|  | Sterile alpha and heat armadillo motif containing 1 | | NODE_5492_length_160_cov_5.487500 | 196 | 8.2E-22 | |  | | | | | |
|  | Nuclear receptor subfamily group member 4 | | NODE_5634_length_1588_cov_16.292191 | 1624 | 0.0 | |  | | | | | |
|  | bai1-associated protein 2-like 1 | | NODE_5708_length_2511_cov_15.760255 | 2547 | 0.0 | |  | | | | | |
|  | Beta 5 | | NODE_5741_length_2963_cov_32.253120 | 2999 | 0.0 | |  | | | | | |
|  | Nuclear receptor subfamily 3 group b member 3 | | NODE_5821_length_1038_cov_13.599229 | 1074 | 5.0E-178 | |  | | | | | |
|  | G-protein coupled receptor 182 | | NODE_5845_length_1908_cov_30.011005 | 1944 | 0.0 | |  | | | | | |
|  | Discoidin domain receptor member 2 | | NODE_5854_length_2996_cov_9.639520 | 3032 | 0.0 | |  | | | | | |
|  | p2y purinoceptor 1 | | NODE_5862_length_274_cov_49.025547 | 310 | 7.1E-57 | |  | | | | | |
|  | Integrin beta | | NODE_5872_length_3282_cov_10.023461 | 3318 | 0.0 | |  | | | | | |
|  | Frizzled homolog 8a | | NODE_5963_length_615_cov_6.052032 | 651 | 2.3E-157 | |  | | | | | |
|  | Hyaluronan-mediated motility receptor | | NODE_5976_length_2813_cov_31.388908 | 2849 | 0.0 | |  | | | | | |
|  | Interleukin-7 receptor subunit alpha | | NODE_6071_length_1871_cov_11.646713 | 1907 | 4.8E-127 | |  | | | | | |
|  | Structural maintenance of chromosomes 2 | | NODE_6108_length_4052_cov_45.547382 | 4088 | 0.0 | |  | | | | | |
|  | Transcriptional coactivator tubedown-100 | | NODE_6127_length_1932_cov_43.273293 | 1968 | 0.0 | |  | | | | | |
|  | Major facilitator superfamily domain-containing protein 6-a | | NODE_6179_length_1140_cov_34.457016 | 1176 | 0.0 | |  | | | | | |
|  | Component of sp100-rs | | NODE_6201_length_1567_cov_16.862158 | 1603 | 6.1E-156 | |  | | | | | |
|  | Membrane guanylyl cyclase | | NODE_6235_length_3404_cov_9.056698 | 3440 | 0.0 | |  | | | | | |
|  | nr1d2a protein | | NODE_6253_length_784_cov_202.017853 | 820 | 6.4E-96 | |  | | | | | |
|  | Autocrine motility factor receptor | | NODE_6286_length_3297_cov_51.961178 | 3333 | 0.0 | |  | | | | | |
|  | Receptor accessory protein 3 | | NODE_6301_length_400_cov_51.927502 | 436 | 3.0E-28 | |  | | | | | |
|  | Chemokine (c-c motif) receptor 6a | | NODE_6354_length_650_cov_23.587692 | 686 | 1.6E-130 | |  | | | | | |
|  | Retinoic acid receptor gamma | | NODE_6355_length_3533_cov_44.780922 | 3569 | 0.0 | |  | | | | | |
|  | Tpa: oncostatin m receptor | | NODE_6534_length_1147_cov_15.544900 | 1183 | 3.4E-61 | |  | | | | | |
|  | G protein-coupled receptor 128 | | NODE_6553_length_213_cov_34.009388 | 249 | 2.2E-19 | |  | | | | | |
|  | Acyl-coenzyme a binding domain containing 3 | | NODE_6579_length_3292_cov_17.157959 | 3328 | 0.0 | |  | | | | | |
|  | Scavenger receptor class member 2 | | NODE_6646_length_652_cov_89.157974 | 688 | 1.0E-17 | |  | | | | | |
|  | Zgc:158862 protein | | NODE_6676_length_845_cov_6.001184 | 881 | 3.3E-81 | |  | | | | | |
|  | Interleukin 13 alpha 2 | | NODE_6771_length_1134_cov_50.741623 | 1170 | 0.0 | |  | | | | | |
|  | prolactin receptor a | | NODE_6844_length_1925_cov_56.584934 | 1961 | 0.0 | |  | | | | | |
|  | p2ry5 protein | | NODE_6926_length_670_cov_6.567164 | 706 | 2.8E-88 | |  | | | | | |
|  | af487829_1 flk1 | | NODE_6937_length_2764_cov_45.797394 | 2800 | 0.0 | |  | | | | | |
|  | Activin a type ib | | NODE_7064_length_1401_cov_41.286224 | 1437 | 0.0 | |  | | | | | |
|  | Loc100158317 protein | | NODE_7146_length_1228_cov_15.845277 | 1264 | 0.0 | |  | | | | | |
|  | nlrx1 receptor | | NODE_7153_length_2950_cov_21.148813 | 2986 | 0.0 | |  | | | | | |
|  | Mannose-6-phosphate receptor-binding protein 1 | | NODE_7154_length_1609_cov_43.435673 | 1645 | 0.0 | |  | | | | | |
|  | Zgc:162182 protein | | NODE_7250_length_467_cov_20.220556 | 503 | 4.1E-42 | |  | | | | | |
|  | si:dkey- protein | | NODE_7254_length_2432_cov_63.379112 | 2468 | 0.0 | |  | | | | | |
|  | Zgc:162129 protein | | NODE_7298_length_3144_cov_19.770992 | 3180 | 0.0 | |  | | | | | |
|  | Cryptochrome 1a | | NODE_7372_length_2218_cov_47.241207 | 2254 | 0.0 | |  | | | | | |
|  | Integrin beta1 subunit-like protein 2 | | NODE_7396_length_74_cov_50.972973 | 110 | 6.9E-13 | |  | | | | | |
|  | Green sensitive cone opsin | | NODE_7437_length_1355_cov_38.895943 | 1391 | 0.0 | |  | | | | | |
|  | Protein nlrc5 | | NODE_7488_length_4002_cov_11.457771 | 4038 | 0.0 | |  | | | | | |
|  | Epidermal growth factor receptor | | NODE_7547_length_4435_cov_43.540474 | 4471 | 0.0 | |  | | | | | |
|  | Activin a receptor type ii-like isoform cra_b | | NODE_7617_length_2523_cov_43.517639 | 2559 | 0.0 | |  | | | | | |
|  | Nuclear receptor 2c2-associated protein ame | | NODE_7628_length_589_cov_20.093378 | 625 | 7.6E-77 | |  | | | | | |
|  | sortilin 1 | | NODE_7707_length_3785_cov_43.530514 | 3821 | 0.0 | |  | | | | | |
|  | spla ryanodine receptor domain and socs box containing 1 | | NODE_7757_length_1791_cov_33.816303 | 1827 | 0.0 | |  | | | | | |
|  | Vitronectin protein 1 | | NODE_7816_length_1673_cov_65.071129 | 1709 | 0.0 | |  | | | | | |
|  | Purinergic receptor fksg79-like | | NODE_7896_length_1638_cov_32.867523 | 1674 | 6.4E-180 | |  | | | | | |
|  | Integrator complex subunit 6 | | NODE_7952_length_3534_cov_16.518110 | 3570 | 0.0 | |  | | | | | |
|  | Interleukin-17 receptor a precursor | | NODE_8002_length_3311_cov_10.254304 | 3347 | 0.0 | |  | | | | | |
|  | Kinase insert domain receptor (a type iii receptor tyrosine kinase) b | | NODE_8012_length_4369_cov_20.674753 | 4405 | 0.0 | |  | | | | | |
|  | Novel protein vertebrate coxsackie virus and adenovirus receptor | | NODE_8033_length_1529_cov_28.666449 | 1565 | 0.0 | |  | | | | | |
|  | Coagulation factor ii receptor-like 1 | | NODE_8107_length_2979_cov_34.347431 | 3015 | 8.4E-162 | |  | | | | | |
|  | Solute carrier family 7 (cationic amino acid y+ system) member 1 | | NODE_8158_length_3362_cov_20.899464 | 3398 | 0.0 | |  | | | | | |
|  | Translocon-associated protein subunit beta precursor | | NODE_8232_length_3141_cov_76.319328 | 3177 | 0.0 | |  | | | | | |
|  | kit receptor | | NODE_8244_length_4732_cov_10.497887 | 4768 | 0.0 | |  | | | | | |
|  | Leukemia inhibitory factor receptor alpha | | NODE_8268_length_2855_cov_10.755867 | 2891 | 0.0 | |  | | | | | |
|  | Novel protein vertebrate g protein-coupled receptor 43 | | NODE_8303_length_1296_cov_149.464508 | 1332 | 0.0 | |  | | | | | |
|  | Macrophage receptor with collagenous structure-like | | NODE_8408_length_655_cov_298.911438 | 691 | 1.2E-38 | |  | | | | | |
|  | Lymphocyte antigen 6 locus g6e | | NODE_8443_length_641_cov_206.886108 | 677 | 1.9E-29 | |  | | | | | |
|  | fc49b10 g-protein coupled receptor | | NODE_8459_length_240_cov_81.550003 | 276 | 4.3E-48 | |  | | | | | |
|  | Gravin | | NODE_8465_length_6654_cov_30.655396 | 6690 | 0.0 | |  | | | | | |
|  | Cytokine receptor family member b4 | | NODE_8493_length_1386_cov_46.581528 | 1422 | 8.3E-35 | |  | | | | | |
|  | Novel protein vertebrate protein tyrosine receptor f | | NODE_8527_length_767_cov_11.561930 | 803 | 1.2E-169 | |  | | | | | |
|  | f11 receptor | | NODE_8542_length_225_cov_173.115555 | 261 | 8.0E-44 | |  | | | | | |
|  | G-protein coupled receptor 183-a | | NODE_8650_length_1952_cov_34.926743 | 1988 | 0.0 | |  | | | | | |
|  | sema immunoglobulin domain short basic 3d | | NODE_8719_length_349_cov_5.704871 | 385 | 6.5E-75 | |  | | | | | |
|  | Activin receptor | | NODE_9462_length_2467_cov_8.956223 | 2503 | 0.0 | |  | | | | | |
|  | af177465_1 estrogen receptor beta2 | | NODE_12081_length_2767_cov_15.284062 | 2803 | 0.0 | |  | | | | | |
|  | Progestin and adipoq receptor family member ix | | NODE_12085_length_2674_cov_14.673897 | 2710 | 7.7E-102 | |  | | | | | |
|  | Progesterone receptor membrane component 2 | | NODE_12355_length_1672_cov_60.053829 | 1708 | 1.1E-88 | |  | | | | | |
|  | Opioid growth factor receptor-like protein 1 | | NODE_12919_length_1598_cov_49.220901 | 1634 | 0.0 | |  | | | | | |
|  | Glucocorticoid receptor | | NODE_14099_length_1517_cov_14.073830 | 1553 | 0.0 | |  | | | | | |
|  | Nuclear progesterone receptor pgr | | NODE_14163_length_3032_cov_11.524736 | 3068 | 0.0 | |  | | | | | |
|  | Estrogen receptor 2a | | NODE_14555_length_874_cov_7.112128  NODE_65608_length_1354_cov_6.203840 | 910  1390 | 1.2E-164  0.0 | |  | | | | | |
|  | Specifically androgen-regulated gene protein | | NODE_14770_length_2474_cov_15.392886 | 2510 | 1.3E-43 | |  | | | | | |
|  | 100 kda thyroid hormone receptor associated protein | | NODE_15072_length_3454_cov_17.016792 | 3490 | 0.0 | |  | | | | | |
|  | Thyroid hormone receptor-associated protein complex 240 kda component-like protein | | NODE_15164_length_4090_cov_30.955257 | 4126 | 0.0 | |  | | | | | |
|  | Growth factor receptor-bound protein 2 | | NODE_15702_length_1112_cov_105.783272 | 1148 | 1.1E-159 | |  | | | | | |
|  | Estrogen receptor 1 | | NODE_16674_length_2112_cov_24.489584 | 2148 | 0.0 | |  | | | | | |
|  | Growth factor receptor-bound protein 10 | | NODE_17527_length_2631_cov_25.741543 | 2667 | 0.0 | |  | | | | | |
|  | Estrogen-related receptor alpha | | NODE_19469_length_2520_cov_27.731747 | 2556 | 0.0 | |  | | | | | |
|  | Estrogen-related receptor beta type 1 | | NODE_19889_length_336_cov_5.964286 | 372 | 2.8E-69 | |  | | | | | |
|  | Prostaglandin e receptor 1 subtype ep1b precursor-like protein | | NODE_20504_length_1900_cov_7.765263 | 1936 | 0.0 | |  | | | | | |
|  | GABA receptor-associated 2 | | NODE_21433_length_712_cov_72.394661 | 748 | 3.5E-78 | |  | | | | | |
|  | Growth hormone receptor | | NODE_25648_length_2781_cov_27.524271 | 2817 | 0.0 | |  | | | | | |
|  | Thyroid hormone receptor alpha | | NODE_26661_length_1568_cov_6.948342 | 1604 | 0.0 | |  | | | | | |
|  | Thyroid hormone receptor beta | | NODE_31438_length_1147_cov_9.989538 | 1183 | 2.1E-161 | |  | | | | | |
|  | Neurogenic locus notch homolog protein 1 | | NODE_26810_length_1374_cov_9.961427 | 1410 | 0.0 | |  | | | | | |
|  | Receptor for egg jelly protein 9 | | NODE_26990_length_435_cov_34.694252 | 471 | 6.1E-67 | |  | | | | | |
|  | Steroid receptor homolog svp 46 | | NODE_27694_length_2582_cov_24.310999 | 2618 | 0.0 | |  | | | | | |
|  | Dihydrotestosterone receptor testicular feminization spinal and bulbar muscular atrophy kennedy disease | | NODE_28201_length_2051_cov_14.548025 | 2087 | 0.0 | |  | | | | | |
|  | Progestin and adipoq receptor family member iii | | NODE_30206_length_673_cov_10.537890 | 709 | 4.8E-79 | |  | | | | | |
|  | Thyroid hormone receptor interactor 13 | | NODE_54396_length_768_cov_15.725261 | 804 | 7.9E-101 | |  | | | | | |
|  | Insulin-like growth factor 1a receptor | | NODE_69830_length_418_cov_5.143541 | 454 | 4.6E-13 | |  | | | | | |
|  | Insulin receptor substrate 1 | | NODE_64907_length_489_cov_5.732106 | 525 | 4.3E-78 | |  | | | | | |
|  | Thyroid hormone receptor interactor 3 | | NODE_66605_length_690_cov_12.269566 | 726 | 6.7E-81 | |  | | | | | |
|  | Ovarian cancer g-protein coupled receptor 1 | | NODE_68813_length_364_cov_5.071429 | 400 | 3.6E-40 | |  | | | | | |
|  | Transferrin receptor 1a | | NODE_10318_length_1196_cov_126.369568 | 1232 | 0.0 | |  | | | | | |
|  | Transferrin receptor 1b | | NODE_11407_length_1124_cov_73.443947 | 1160 | 0.0 | |  | | | | | |
| ***Embryonic development related Proteins*** | | | | | | | | |  | | | |
|  | Neurogenic locus notch homolog protein 1 flags: precursor | | NODE_316_length_1750_cov_8.647429 | 1786 | 0.0 | |  | | | | | |
|  | Eukaryotic translation elongation factor 2 | | NODE_392_length_1124_cov_1985.912842 | 1160 | 0.0 | |  | | | | | |
|  | Ribosomal protein s4 | | NODE_400_length_103_cov_3943.485352 | 139 | 5.8E-25 | |  | | | | | |
|  | 40s ribosomal protein x isoform | | NODE_443_length_119_cov_4379.335938 | 155 | 7.6E-28 | |  | | | | | |
|  | Nipped-b like b | | NODE_465_length_6536_cov_31.942013 | 6572 | 0.0 | |  | | | | | |
|  | Novel protein vertebrate radixin | | NODE_9565_length_1878_cov_41.744942 | 1914 | 3.9E-65 | |  | | | | | |
|  | Ribosomal protein l5a | | NODE_1836_length_320_cov_1350.671875 | 356 | 2.3E-40 | |  | | | | | |
|  | Prostaglandin e synthase | | NODE_1635_length_1302_cov_23.970047 | 1338 | 2.7E-87 | |  | | | | | |
|  | N-acetylglucosamine-1-phosphotransferase subunits alpha beta | | NODE_1742_length_2142_cov_14.718020 | 2178 | 0.0 | |  | | | | | |
|  | Na+ K+ alpha 1 polypeptide | | NODE_1865_length_768_cov_732.575500 | 804 | 9.8E-158 | |  | | | | | |
|  | v-maf musculoaponeurotic fibrosarcoma oncogene protein b | | NODE_1868_length_778_cov_64.951157 | 814 | 2.8E-76 | |  | | | | | |
|  | cop9 constitutive photomorphogenic homolog subunit 3 | | NODE_1915_length_1599_cov_57.241402 | 1635 | 0.0 | |  | | | | | |
|  | Zgc:103456 protein | | NODE_2068_length_272_cov_200.922791 | 308 | 1.1E-65 | |  | | | | | |
|  | brahma protein 1 | | NODE_2176_length_4930_cov_51.235092 | 4966 | 0.0 | |  | | | | | |
|  | Type i enveloping like | | NODE_2234_length_427_cov_353.121765 | 463 | 2.1E-87 | |  | | | | | |
|  | Nonmuscle myosin heavy chain | | NODE_2345_length_3338_cov_40.361893 | 3374 | 0.0 | |  | | | | | |
|  | af297180_1 na pi cotransporter i-iib2 | | NODE_2447_length_2538_cov_13.425926 | 2574 | 0.0 | |  | | | | | |
|  | D-box binding protein 2 | | NODE_2502_length_1915_cov_43.324802 | 1951 | 1.3E-155 | |  | | | | | |
|  | Methyl- binding domain protein 3 | | NODE_2543_length_878_cov_52.275627 | 914 | 1.1E-138 | |  | | | | | |
|  | ezrin like | | NODE_2571_length_1552_cov_110.239044 | 1588 | 8.0E-154 | |  | | | | | |
|  | sparc precursor | | NODE_2810_length_1113_cov_282.271332 | 1149 | 0.0 | |  | | | | | |
|  | myst histone acetyltransferase (monocytic leukemia) 3 | | NODE_3023_length_5308_cov_30.818388 | 5344 | 0.0 | |  | | | | | |
|  | swi snf matrix actin dependent regulator of subfamily member 5 | | NODE_3123_length_2660_cov_71.039848 | 2696 | 0.0 | |  | | | | | |
|  | dsh homolog 2 | | NODE_3176_length_661_cov_10.717095 | 697 | 1.1E-18 | |  | | | | | |
|  | Novel myosin family protein | | NODE_56782_length_641_cov_6.854914 | 677 | 3.8E-125 | |  | | | | | |
|  | Fibronectin 1b | | NODE_3218_length_3844_cov_165.552551 | 3880 | 0.0 | |  | | | | | |
|  | swi snf matrix actin dependent regulator of subfamily member isoform cra_c | | NODE_3311_length_434_cov_32.327190 | 470 | 1.7E-22 | |  | | | | | |
|  | Heavy polypeptide cardiac alpha | | NODE_3316_length_5853_cov_53.863148 | 5889 | 0.0 | |  | | | | | |
|  | Angiomotin-like 2 | | NODE_3623_length_4028_cov_20.284012 | 4064 | 0.0 | |  | | | | | |
|  | Novel protein with hect-domain (ubiquitin-transferase) | | NODE_3869_length_4258_cov_53.915218 | 4294 | 0.0 | |  | | | | | |
|  | af350072_1 erythroid band 3 anion exchanger 1 | | NODE_3889_length_1817_cov_371.706665 | 1853 | 0.0 | |  | | | | | |
|  | Ell associated factor 2 | | NODE_3943_length_1498_cov_26.965286 | 1534 | 2.5E-98 | |  | | | | | |
|  | Follistatin-like 2 | | NODE_4071_length_764_cov_69.573296 | 800 | 1.0E-90 | |  | | | | | |
|  | Transcription factor val ame: full=valentino | | NODE_4086_length_616_cov_24.735390 | 652 | 7.0E-57 | |  | | | | | |
|  | Secreted frizzled-related protein 1 | | NODE_4129_length_1511_cov_6.187293 | 1547 | 6.1E-158 | |  | | | | | |
|  | SIP1a | | NODE_4406_length_3158_cov_25.731159 | 3194 | 0.0 | |  | | | | | |
|  | Zinc transporter liv1 | | NODE_4570_length_1362_cov_76.772392 | 1398 | 3.4E-136 | |  | | | | | |
|  | Ribosomal protein s8 | | NODE_4620_length_102_cov_4321.019531 | 138 | 4.8E-25 | |  | | | | | |
|  | Caveolin 1 | | NODE_4704_length_1151_cov_41.848827 | 1187 | 1.7E-127 | |  | | | | | |
|  | Transcription factor 7-like 1-a | | NODE_4810_length_1837_cov_12.156233 | 1873 | 0.0 | |  | | | | | |
|  | Homeodomain protein cdx1a | | NODE_4865_length_993_cov_15.887211 | 1029 | 7.0E-81 | |  | | | | | |
|  | Erythrocyte membrane protein band like 5 | | NODE_4875_length_2424_cov_23.218235 | 2460 | 6.5E-165 | |  | | | | | |
|  | Eukaryotic translation initiation factor 3 subunit e-a | | NODE_4915_length_156_cov_222.903839 | 192 | 1.2E-31 | |  | | | | | |
|  | Coatomer protein subunit beta 1 | | NODE_4983_length_1179_cov_105.280746 | 1215 | 0.0 | |  | | | | | |
|  | pou domain protein class v transcription factor 1 | | NODE_5208_length_1394_cov_116.715927 | 1430 | 0.0 | |  | | | | | |
|  | Myosin viia | | NODE_5219_length_3122_cov_13.249840 | 3158 | 0.0 | |  | | | | | |
|  | af281858_1 endothelin 1 | | NODE_5228_length_1328_cov_20.155121 | 1364 | 7.1E-87 | |  | | | | | |
|  | map3k7 protein | | NODE_7462_length_2078_cov_55.478825 | 2114 | 1.6E-86 | |  | | | | | |
|  | sfrs1l protein | | NODE_5478_length_207_cov_85.594200 | 243 | 8.9E-42 | |  | | | | | |
|  | Non-erythrocytic isoform cra_d | | NODE_5650_length_7292_cov_85.123421 | 7328 | 0.0 | |  | | | | | |
|  | 5-aminolevulinate erythroid- mitochondrial | | NODE_5769_length_1156_cov_442.923004 | 1192 | 0.0 | |  | | | | | |
|  | Solute carrier family 12 (potassium chloride transporters) member 2 | | NODE_66574_length_235_cov_34.982979 | 271 | 1.6E-52 | |  | | | | | |
|  | T-box 2b | | NODE_6372_length_703_cov_8.533428 | 739 | 7.2E-150 | |  | | | | | |
|  | Cytochrome subfamily polypeptide 1 | | NODE_6411_length_1747_cov_44.004578 | 1783 | 0.0 | |  | | | | | |
|  | af286374_1 na+ k+ atpase alpha subunit isoform 3 | | NODE_61390_length_784_cov_45.423470 | 820 | 4.2E-139 | |  | | | | | |
|  | Axis inhibition protein 2 | | NODE_6705_length_3657_cov_13.113754 | 3693 | 0.0 | |  | | | | | |
|  | Ring finger protein 2 | | NODE_6839_length_776_cov_82.706184 | 812 | 4.8E-123 | |  | | | | | |
|  | stat3 protein | | NODE_6884_length_1105_cov_78.871490 | 1141 | 0.0 | |  | | | | | |
|  | Disrupted in schizophrenia 1 | | NODE_6978_length_2687_cov_9.831410 | 2723 | 0.0 | |  | | | | | |
|  | Transforming growth factor beta-1-induced transcript 1 protein | | NODE_7026_length_3688_cov_20.525217 | 3724 | 0.0 | |  | | | | | |
|  | Erythrocyte membrane protein band (elliptocytosis rh-linked) | | NODE_7053_length_2595_cov_51.759537 | 2631 | 5.0E-173 | |  | | | | | |
|  | Autophagy beclin 1 regulator 1 | | NODE_7340_length_4133_cov_12.173240 | 4169 | 0.0 | |  | | | | | |
|  | Tripartite motif protein trim33 | | NODE_7345_length_3203_cov_21.656260 | 3239 | 0.0 | |  | | | | | |
|  | Exostoses 1b | | NODE_7419_length_2220_cov_32.839638 | 2256 | 0.0 | |  | | | | | |
|  | Polymerase i and transcript release factor | | NODE_7434_length_2936_cov_51.850819 | 2972 | 1.5E-170 | |  | | | | | |
|  | N-myristoyltransferase 1 | | NODE_78667_length_1823_cov_43.592430 | 1859 | 0.0 | |  | | | | | |
|  | kelch-like protein 12 | | NODE_7540_length_2787_cov_14.510943 | 2823 | 0.0 | |  | | | | | |
|  | Ribosomal protein s3a | | NODE_7551_length_127_cov_3577.811035 | 163 | 6.6E-29 | |  | | | | | |
|  | RNA polymerase-associated protein ctr9 homolog | | NODE_7654_length_3850_cov_41.250389 | 3886 | 0.0 | |  | | | | | |
|  | sec23 homolog b | | NODE_7712_length_3247_cov_54.876808 | 3283 | 0.0 | |  | | | | | |
|  | sec23a | | NODE_8116_length_1541_cov_28.861130 | 1577 | 0.0 | |  | | | | | |
|  | proteasome ( macropain) 26s 4 | | NODE_7928_length_1571_cov_83.301720 | 1607 | 0.0 | |  | | | | | |
|  | Lysocardiolipin acyltransferase 1 | | NODE_8332_length_956_cov_19.351465 | 992 | 1.5E-169 | |  | | | | | |
|  | Autosomal dominant 5 | | NODE_8409_length_794_cov_50.080605 | 830 | 2.8E-128 | |  | | | | | |
|  | Suppressor of ty 6 homolog | | NODE_8504_length_5946_cov_34.487892 | 5982 | 0.0 | |  | | | | | |
|  | traf2 and nck interacting kinase like | | NODE_8617_length_1727_cov_20.308628 | 1763 | 2.9E-33 | |  | | | | | |
|  | Transcription factor sox9a | | NODE_8667_length_256_cov_5.714844 | 292 | 2.0E-16 | |  | | | | | |
|  | RNA polymerase-associated protein rtf1 homolog | | NODE_8757_length_2083_cov_51.028324 | 2119 | 0.0 | |  | | | | | |
|  | Small ubiquitin-related modifier 1 precursor | | NODE_8936_length_1094_cov_85.652649 | 1130 | 1.3E-61 | |  | | | | | |
|  | Receptor tyrosine kinase-like orphan receptor 2 | | NODE_9002_length_2693_cov_15.329001 | 2729 | 0.0 | |  | | | | | |
|  | Exostosin-2 | | NODE_9086_length_3221_cov_17.276932 | 3257 | 0.0 | |  | | | | | |
|  | Cysteine and glycine-rich protein 1 | | NODE_9671_length_1013_cov_83.985191 | 1049 | 5.7E-75 | |  | | | | | |
|  | Excisionn repair cross-complementing rodent repair complementation group 2 | | NODE_9371_length_2524_cov_9.979398 | 2560 | 0.0 | |  | | | | | |
|  | Calcitenin receptor-like receptor | | NODE_9383_length_2598_cov_12.233642 | 2634 | 0.0 | |  | | | | | |
|  | Trans-acting transcription factor 1 | | NODE_9389_length_1667_cov_51.117577 | 1703 | 2.0E-169 | |  | | | | | |
|  | mgc115546 protein | | NODE_9581_length_999_cov_24.711712 | 1035 | 5.5E-170 | |  | | | | | |
|  | smad4 protein | | NODE_9590_length_110_cov_5.563636 | 146 | 3.4E-29 | |  | | | | | |
|  | Novel protein vertebrate amyloid beta precursor-like protein 2 | | NODE_9744_length_616_cov_142.092529 | 652 | 1.5E-64 | |  | | | | | |
|  | Solute carrier family 4 anion exchanger member 1b | | NODE_9853_length_3186_cov_10.538607 | 3222 | 0.0 | |  | | | | | |
|  | Cyclase-associated protein-1 | | NODE_10400_length_2033_cov_185.793411 | 2069 | 0.0 | |  | | | | | |
|  | Mitogen-activated protein kinase 14a | | NODE_10457_length_407_cov_74.977890 | 443 | 2.3E-55 | |  | | | | | |
|  | wnt8-like protein 1 | | NODE_10617_length_1674_cov_10.999403 | 1710 | 0.0 | |  | | | | | |
|  | sp3 transcription isoform cra_d | | NODE_10699_length_1372_cov_39.741253 | 1408 | 5.4E-98 | |  | | | | | |
|  | swi snf matrix actin dependent regulator of subfamily member 1 | | NODE_10726_length_1209_cov_57.379654 | 1245 | 0.0 | |  | | | | | |
|  | mad homolog 2 | | NODE_10796_length_2260_cov_47.934513 | 2296 | 0.0 | |  | | | | | |
|  | smg5 homolog nonsense mediated mrna decay factor protein | | NODE_10836_length_2163_cov_18.321775 | 2199 | 0.0 | |  | | | | | |
|  | Mitogen-activated protein kinase 1 | | NODE_10873_length_2067_cov_51.475086 | 2103 | 0.0 | |  | | | | | |
|  | Germ cell nuclear factor b short=gcnf-b | | NODE_11344_length_98_cov_5.102041 | 134 | 6.7E-20 | |  | | | | | |
|  | hnf1 homeobox b | | NODE_11358_length_722_cov_17.965374 | 758 | 4.9E-121 | |  | | | | | |
|  | Serine arginine-rich splicing factor 1b | | NODE_11433_length_922_cov_61.752712 | 958 | 2.3E-91 | |  | | | | | |
|  | slc25a37 protein | | NODE_11448_length_433_cov_24.958429 | 469 | 1.3E-56 | |  | | | | | |
|  | Hematopoietically expressed homeobox | | NODE_11570_length_726_cov_45.601929 | 762 | 7.0E-98 | |  | | | | | |
|  | Mediator complex subunit 21 | | NODE_11705_length_739_cov_28.523680 | 775 | 3.2E-90 | |  | | | | | |
|  | Guanine nucleotide binding alpha 12-like | | NODE_11907_length_910_cov_51.138462 | 946 | 1.8E-87 | |  | | | | | |
|  | Transcription factor 2 | | NODE_12179_length_1983_cov_49.621784 | 2019 | 0.0 | |  | | | | | |
|  | Twisted gastrulation protein homolog 1-a precursor | | NODE_12182_length_656_cov_52.025913 | 692 | 6.2E-108 | |  | | | | | |
|  | Vasodilator-stimulated phosphoprotein | | NODE_12286_length_1118_cov_23.375671 | 1154 | 4.3E-84 | |  | | | | | |
|  | Novel protein vertebrate hir histone cell cycle regulation defective homolog a | | NODE_12345_length_3642_cov_22.375343 | 3678 | 0.0 | |  | | | | | |
|  | gnas complex locus | | NODE_12346_length_1349_cov_18.476650 | 1385 | 1.4E-38 | |  | | | | | |
|  | lim domain binding 3b | | NODE_12386_length_718_cov_73.990250 | 754 | 1.4E-58 | |  | | | | | |
|  | mgc107891 protein | | NODE_12600_length_2428_cov_74.960876 | 2464 | 0.0 | |  | | | | | |
|  | la ribonucleoprotein domain member 6 | | NODE_12716_length_102_cov_14.205882 | 138 | 2.5E-22 | |  | | | | | |
|  | ets variant gene 4 (e1a enhancer binding e1af) | | NODE_12720_length_1824_cov_21.197916 | 1860 | 0.0 | |  | | | | | |
|  | af437316_1 frizzled-7a | | NODE_12947_length_2640_cov_28.784090 | 2676 | 0.0 | |  | | | | | |
|  | Caudal type homeodomain protein | | NODE_13131_length_1123_cov_21.739983 | 1159 | 4.2E-144 | |  | | | | | |
|  | Cadherin-2 | | NODE_13149_length_1142_cov_5.307355 | 1178 | 0.0 | |  | | | | | |
|  | sp3 protein | | NODE_13264_length_1637_cov_27.122786 | 1673 | 5.9E-101 | |  | | | | | |
|  | Prospero-related homeobox 1 | | NODE_13271_length_857_cov_7.607934 | 893 | 5.1E-176 | |  | | | | | |
|  | Endothelial pas domain protein 1 | | NODE_13374_length_1194_cov_61.391121 | 1230 | 0.0 | |  | | | | | |
|  | Synovial apoptosis inhibitor synoviolin | | NODE_13443_length_1092_cov_33.299450 | 1128 | 0.0 | |  | | | | | |
|  | aplp1 protein | | NODE_13491_length_2661_cov_20.647125 | 2697 | 1.6E-58 | |  | | | | | |
|  | Zgc:153035 protein ( hepatic leukemia factor) | | NODE_13565_length_1057_cov_15.063387 | 1093 | 1.5E-77 | |  | | | | | |
|  | Integrin alpha5 | | NODE_13579_length_3439_cov_40.119511 | 3475 | 0.0 | |  | | | | | |
|  | smt3 suppressor of mif two 3 homolog 3 | | NODE_13602_length_678_cov_55.255161 | 714 | 2.6E-61 | |  | | | | | |
|  | Coiled-coil domain containing 47 | | NODE_13682_length_1855_cov_78.380592 | 1891 | 0.0 | |  | | | | | |
|  | E1A binding protein p300 | | NODE_13962_length_4551_cov_14.710833 | 4587 | 0.0 | |  | | | | | |
|  | top2a protein | | NODE_14143_length_4336_cov_16.406136 | 4372 | 0.0 | |  | | | | | |
|  | Protein chameleon | | NODE_14151_length_4548_cov_9.412049 | 4584 | 0.0 | |  | | | | | |
|  | Myosin regulatory light chain interacting protein | | NODE_14186_length_1170_cov_48.779488 | 1206 | 0.0 | |  | | | | | |
|  | Mind bomb | | NODE_14195_length_2295_cov_19.090631 | 2331 | 0.0 | |  | | | | | |
|  | pbxy homeodomain protein | | NODE_14285_length_448_cov_12.859375 | 484 | 1.2E-90 | |  | | | | | |
|  | Transforming protein precursor | | NODE_14388_length_678_cov_252.911499 | 714 | 5.0E-139 | |  | | | | | |
|  | Yes-related kinase | | NODE_14459_length_132_cov_29.393940 | 168 | 1.3E-30 | |  | | | | | |
|  | Notch1a protein | | NODE_14548_length_735_cov_7.895238 | 771 | 0.0 | |  | | | | | |
|  | kif23 protein | | NODE_14686_length_1293_cov_126.636505 | 1329 | 0.0 | |  | | | | | |
|  | Lunatic fringe | | NODE_14707_length_2746_cov_14.130371 | 2782 | 0.0 | |  | | | | | |
|  | Sphingosine 1-phosphate receptor 2 | | NODE_14794_length_3050_cov_13.731148 | 3086 | 0.0 | |  | | | | | |
|  | udp-glucose dehydrogenase | | NODE_14999_length_1839_cov_15.584013 | 1875 | 0.0 | |  | | | | | |
|  | Surfeit locus protein 1 | | NODE_15246_length_1052_cov_14.157795 | 1088 | 0.0 | |  | | | | | |
|  | Fibronectin 1a isoform 1 | | NODE_15252_length_4255_cov_26.515158 | 4291 | 0.0 | |  | | | | | |
|  | Zgc:158737 protein | | NODE_15259_length_831_cov_24.981949 | 867 | 4.1E-95 | |  | | | | | |
|  | Topoisomerase like | | NODE_15269_length_888_cov_25.935810 | 924 | 9.1E-179 | |  | | | | | |
|  | smg-7 nonsense mediated mrna decay factor | | NODE_15295_length_4172_cov_27.960930 | 4208 | 0.0 | |  | | | | | |
|  | E3 ubiquitin-protein ligase mib1 | | NODE_15561_length_1430_cov_21.147552 | 1466 | 0.0 | |  | | | | | |
|  | b chain smad1 crystal structure reveals the details of bmp signaling pathway | | NODE_15617_length_561_cov_6.333333 | 597 | 3.6E-139 | |  | | | | | |
|  | Mak10 amino-acid n-acetyltransferase subunit | | NODE_15677_length_2365_cov_15.689641 | 2401 | 0.0 | |  | | | | | |
|  | Knypek | | NODE_15898_length_4533_cov_27.250607 | 4569 | 0.0 | |  | | | | | |
|  | TATA-binding protein 2 | | NODE_15984_length_1368_cov_21.042398 | 1404 | 0.0 | |  | | | | | |
|  | Sodium-potassium-chloride cotransporter 1 | | NODE_16084_length_1007_cov_35.990070 | 1043 | 3.3E-81 | |  | | | | | |
|  | Adducin 1 | | NODE_16180_length_372_cov_33.301075 | 408 | 8.8E-74 | |  | | | | | |
|  | mbd3 protein | | NODE_16302_length_1576_cov_9.986675 | 1612 | 4.7E-165 | |  | | | | | |
|  | tie1 protein | | NODE_16388_length_4166_cov_11.218435 | 4202 | 0.0 | |  | | | | | |
|  | Sprouty homolog 2 | | NODE_16412_length_1165_cov_23.967382 | 1201 | 9.5E-76 | |  | | | | | |
|  | Methylcytosine dioxygenase tet2 | | NODE_16603_length_6820_cov_7.963343 | 6856 | 0.0 | |  | | | | | |
|  | Lim domain transcription factor lmo4 | | NODE_16773_length_1329_cov_7.566591 | 1365 | 1.4E-109 | |  | | | | | |
|  | pou-2 | | NODE_16943_length_80_cov_32.012501 | 116 | 4.3E-8 | |  | | | | | |
|  | Enabled homolog | | NODE_17171_length_1141_cov_22.082384 | 1177 | 8.7E-82 | |  | | | | | |
|  | Mitogen-activated protein kinase kinase kinase 7 interacting protein 1 | | NODE_17205_length_1293_cov_13.637278 | 1329 | 0.0 | |  | | | | | |
|  | Neogenin | | NODE_17451_length_322_cov_5.978261 | 358 | 8.4E-46 | |  | | | | | |
|  | af152001_1 notch3 | | NODE_17725_length_6515_cov_7.343976 | 6551 | 0.0 | |  | | | | | |
|  | protein canopy-1 | | NODE_17782_length_718_cov_42.703342 | 754 | 1.5E-84 | |  | | | | | |
|  | Tubby-like protein 3 | | NODE_17841_length_1204_cov_14.345515 | 1240 | 6.6E-63 | |  | | | | | |
|  | Chromodomain-helicase-DNA-binding protein 7 | | NODE_17898_length_1652_cov_22.571428 | 1688 | 1.3E-134 | |  | | | | | |
|  | Novel protein vertebrate pygopus homolog 2 | | NODE_17927_length_933_cov_25.493032 | 969 | 2.1E-18 | |  | | | | | |
|  | fa48a_chick | | NODE_18004_length_4036_cov_17.519325 | 4072 | 0.0 | |  | | | | | |
|  | Rapamycin-insensitive companion of mtor | | NODE_18047_length_6422_cov_12.049361 | 6458 | 0.0 | |  | | | | | |
|  | Delta-like protein c | | NODE_18054_length_2684_cov_10.381893 | 2720 | 0.0 | |  | | | | | |
|  | Novel protein endonuclease g | | NODE_18270_length_1225_cov_7.087347 | 1261 | 0.0 | |  | | | | | |
|  | E74-like factor 3 | | NODE_18393_length_1851_cov_40.715290 | 1887 | 0.0 | |  | | | | | |
|  | Transcription factor 7-like 1-b | | NODE_18823_length_2047_cov_32.954567 | 2083 | 0.0 | |  | | | | | |
|  | Heavy chain non-muscle | | NODE_19015_length_5229_cov_52.669727 | 5265 | 0.0 | |  | | | | | |
|  | Kinesin family member 1b | | NODE_19054_length_1675_cov_5.497313 | 1711 | 0.0 | |  | | | | | |
|  | Nonmuscle myosin heavy chain b | | NODE_19318_length_1276_cov_27.305643 | 1312 | 0.0 | |  | | | | | |
|  | bmi1 polycomb ring finger oncogene | | NODE_19508_length_1766_cov_20.849377 | 1802 | 0.0 | |  | | | | | |
|  | wd and tetratricopeptide repeats isoform cra_b | | NODE_20091_length_2681_cov_13.860499 | 2717 | 0.0 | |  | | | | | |
|  | Churchill domain containing 1 | | NODE_20330_length_685_cov_13.912409 | 721 | 2.8E-79 | |  | | | | | |
|  | Ubiquinone biosynthesis protein coq7 homolog | | NODE_20402_length_1253_cov_10.851556 | 1289 | 8.0E-98 | |  | | | | | |
|  | Zinc finger homeobox isoform cra_a | | NODE_20467_length_1357_cov_30.464996 | 1393 | 2.4E-96 | |  | | | | | |
|  | Hepatocyte nuclear factor 1-beta-b | | NODE_20666_length_310_cov_25.729033 | 346 | 2.2E-30 | |  | | | | | |
|  | Myosin via | | NODE_20694_length_1225_cov_24.188572 | 1261 | 1.3E-121 | |  | | | | | |
|  | fmr1 protein | | NODE_20804_length_1449_cov_29.527260 | 1485 | 0.0 | |  | | | | | |
|  | Orthodenticle homolog 1 | | NODE_20984_length_1177_cov_32.444351 | 1213 | 9.6E-145 | |  | | | | | |
|  | hand2 protein | | NODE_21162_length_1079_cov_6.640408 | 1115 | 2.0E-117 | |  | | | | | |
|  | Proprotein convertase subtilisin kexin type 5 | | NODE_21184_length_2279_cov_9.766564 | 2315 | 0.0 | |  | | | | | |
|  | bmp type iia receptor | | NODE_21268_length_824_cov_6.768204 | 860 | 3.6E-95 | |  | | | | | |
|  | af305882_1 kh domain containing RNA-binding protein fmr1 | | NODE_21338_length_188_cov_26.781916 | 224 | 5.3E-18 | |  | | | | | |
|  | Zinc finger protein 703 | | NODE_21427_length_664_cov_6.667169 | 700 | 7.7E-85 | |  | | | | | |
|  | Activin receptor iib | | NODE_21589_length_1525_cov_8.146230 | 1561 | 0.0 | |  | | | | | |
|  | Notch homolog translocation-associated | | NODE_21592_length_1976_cov_10.645243 | 2012 | 0.0 | |  | | | | | |
|  | b chain crystal structure of the btb domain from the miz-1zbtb17 transcription regulator | | NODE_21601_length_1037_cov_13.643202 | 1073 | 7.5E-136 | |  | | | | | |
|  | gata1 protein | | NODE_21602_length_1651_cov_28.755905 | 1687 | 0.0 | |  | | | | | |
|  | Guanine nucleotide binding protein (g protein) alpha activating activity olfactory type | | NODE_21967_length_242_cov_17.289257 | 278 | 2.6E-29 | |  | | | | | |
|  | Zinc finger transcription factor 24hpf | | NODE_21974_length_3961_cov_13.698309 | 3997 | 0.0 | |  | | | | | |
|  | ephrin b1 | | NODE_22196_length_922_cov_7.433839 | 958 | 1.9E-60 | |  | | | | | |
|  | Zgc:64042 protein | | NODE_22277_length_2748_cov_11.578603 | 2784 | 0.0 | |  | | | | | |
|  | apelin receptor a | | NODE_22609_length_1640_cov_13.284756 | 1676 | 0.0 | |  | | | | | |
|  | Myocyte enhancer factor 2a | | NODE_22663_length_132_cov_34.742424 | 168 | 2.1E-33 | |  | | | | | |
|  | Neurofibromin 2 | | NODE_22748_length_2818_cov_25.389282 | 2854 | 0.0 | |  | | | | | |
|  | RNA binding motif protein 22 | | NODE_52365_length_1820_cov_12.468681 | 1856 | 0.0 | |  | | | | | |
|  | lrp5 protein | | NODE_23052_length_1937_cov_8.942179 | 1973 | 0.0 | |  | | | | | |
|  | lim domain-binding protein 1-like | | NODE_23104_length_969_cov_7.358101 | 1005 | 0.0 | |  | | | | | |
|  | t-cell factor 7 isoform c | | NODE_23495_length_1212_cov_36.190594 | 1248 | 7.2E-167 | |  | | | | | |
|  | Survival of motor neuron protein interacting protein 1 | | NODE_23783_length_1127_cov_16.415262 | 1163 | 2.1E-170 | |  | | | | | |
|  | Protein tyrosine receptor r isoform 2 | | NODE_23908_length_706_cov_15.953258 | 742 | 3.0E-52 | |  | | | | | |
|  | Transcription termination RNA polymerase ii | | NODE_24045_length_3675_cov_17.391020 | 3711 | 0.0 | |  | | | | | |
|  | Myosin xv | | NODE_24145_length_1201_cov_5.190674 | 1237 | 0.0 | |  | | | | | |
|  | rplp0 protein | | NODE_24320_length_183_cov_2430.366211 | 219 | 2.8E-38 | |  | | | | | |
|  | Synembryn-a | | NODE_24409_length_651_cov_5.732719 | 687 | 8.4E-129 | |  | | | | | |
|  | Endothelial sphingolipid g-protein-coupled 1 | | NODE_24614_length_668_cov_8.488024 | 704 | 6.4E-98 | |  | | | | | |
|  | b chain crystal structure of edeya2 | | NODE_24787_length_1289_cov_6.463925 | 1325 | 1.2E-150 | |  | | | | | |
|  | nemo-like kinase | | NODE_24860_length_1857_cov_9.355412 | 1893 | 0.0 | |  | | | | | |
|  | Lysyl oxidase-like protein 1 | | NODE_25091_length_1497_cov_8.156313 | 1533 | 8.5E-117 | |  | | | | | |
|  | Cysteine-rich motor neuron 1 | | NODE_25239_length_206_cov_9.485436 | 242 | 4.9E-39 | |  | | | | | |
|  | Transport protein sec23a | | NODE_25305_length_954_cov_18.194969 | 990 | 0.0 | |  | | | | | |
|  | mediator of RNA polymerase ii transcription subunit 12 | | NODE_25566_length_822_cov_31.559610 | 858 | 4.8E-70 | |  | | | | | |
|  | Thrombomodulin precursor | | NODE_25788_length_1063_cov_6.557855 | 1099 | 5.2E-21 | |  | | | | | |
|  | Leucine-zipper protein | | NODE_25872_length_3248_cov_15.681034 | 3284 | 6.4E-92 | |  | | | | | |
|  | csrp2 binding protein | | NODE_26129_length_1921_cov_10.309734 | 1957 | 0.0 | |  | | | | | |
|  | zbtb16 protein | | NODE_26403_length_2361_cov_22.793732 | 2397 | 4.3E-63 | |  | | | | | |
|  | Neurogenic locus notch homolog protein 1 flags: precursor | | NODE_26810_length_1374_cov_9.961427 | 1410 | 0.0 | |  | | | | | |
|  | Glucocorticoid receptor DNA binding factor 1 | | NODE_27087_length_1857_cov_12.987076 | 1893 | 0.0 | |  | | | | | |
|  | Forkhead box h1 | | NODE_27172_length_1978_cov_32.449444 | 2014 | 0.0 | |  | | | | | |
|  | Ligase ATP-dependent | | NODE_27468_length_837_cov_5.215054 | 873 | 6.3E-165 | |  | | | | | |
|  | snai1a protein | | NODE_28170_length_862_cov_8.734339 | 898 | 7.4E-157 | |  | | | | | |
|  | gcn5 general control of amino acid synthesis-like 2 | | NODE_28243_length_3480_cov_11.605747 | 3516 | 0.0 | |  | | | | | |
|  | myc target protein 1 homolog | | NODE_28319_length_461_cov_5.902386 | 497 | 6.9E-20 | |  | | | | | |
|  | Twisted gastrulation protein homolog 1-a flags: precursor | | NODE_28478_length_491_cov_8.439919 | 527 | 2.0E-77 | |  | | | | | |
|  | pdlim7 protein | | NODE_28491_length_743_cov_8.325706 | 779 | 5.3E-80 | |  | | | | | |
|  | AT-rich interactive domain-containing protein 5b | | NODE_29073_length_613_cov_6.378467 | 649 | 4.9E-123 | |  | | | | | |
|  | Connexin 43 | | NODE_29106_length_1170_cov_17.161539 | 1206 | 0.0 | |  | | | | | |
|  | Guanine nucleotide binding protein (g protein) alpha 13 | | NODE_29279_length_1979_cov_34.262760 | 2015 | 0.0 | |  | | | | | |
|  | mkl myocardin-like isoform cra_d | | NODE_29442_length_650_cov_9.781538 | 686 | 1.0E-64 | |  | | | | | |
|  | Forkhead box c1a | | NODE_29591_length_849_cov_6.246172 | 885 | 1.7E-87 | |  | | | | | |
|  | Forkhead box a2 | | NODE_29601_length_1641_cov_7.378428 | 1677 | 0.0 | |  | | | | | |
|  | Na+ K+ beta 2a polypeptide | | NODE_29686_length_655_cov_5.424428 | 691 | 6.3E-108 | |  | | | | | |
|  | sry (sex determining region y)-box 7 | | NODE_30022_length_666_cov_10.244744 | 702 | 2.6E-113 | |  | | | | | |
|  | b chain crystal structure of the rhogap domain of human glucocorticoid receptor dna-binding factor 1 | | NODE_30467_length_666_cov_5.498498 | 702 | 5.5E-140 | |  | | | | | |
|  | lim domain only 4 | | NODE_30482_length_1599_cov_9.318950 | 1635 | 2.9E-108 | |  | | | | | |
|  | mgc68699 protein | | NODE_30680_length_149_cov_2312.919434 | 185 | 1.9E-33 | |  | | | | | |
|  | mad homolog 1 | | NODE_31046_length_974_cov_5.394250 | 1010 | 4.0E-114 | |  | | | | | |
|  | G-protein alpha 12 | | NODE_31253_length_882_cov_6.157597 | 918 | 4.1E-96 | |  | | | | | |
|  | af329830_1 t-box transcription factor eomesodermin | | NODE_31868_length_1155_cov_46.689178 | 1191 | 0.0 | |  | | | | | |
|  | swi snf-related matrix-associated actin-dependent regulator of chromatin subfamily a-like protein 1 | | NODE_32078_length_2363_cov_8.107491 | 2399 | 0.0 | |  | | | | | |
|  | eph receptor a2 | | NODE_32081_length_4416_cov_10.886549 | 4452 | 0.0 | |  | | | | | |
|  | Topoisomerase I | | NODE_32181_length_658_cov_6.003039 | 694 | 2.5E-114 | |  | | | | | |
|  | Chordin | | NODE_32367_length_1790_cov_7.497766 | 1826 | 0.0 | |  | | | | | |
|  | Sphingosine-1-phosphate receptor 1 | | NODE_32535_length_920_cov_9.279347 | 956 | 4.0E-114 | |  | | | | | |
|  | Transmembrane anterior posterior transformation protein 1 homolog | | NODE_32607_length_2205_cov_6.627211 | 2241 | 0.0 | |  | | | | | |
|  | dvl3 protein | | NODE_34292_length_1316_cov_16.599545 | 1352 | 5.9E-149 | |  | | | | | |
|  | Amyotrophic lateral sclerosis 2 protein homolog | | NODE_34617_length_1407_cov_7.312011 | 1443 | 0.0 | |  | | | | | |
|  | Serine threonine-protein kinase plk4 | | NODE_35068_length_541_cov_14.757855 | 577 | 1.8E-79 | |  | | | | | |
|  | gli-kruppel family member gli2a | | NODE_35084_length_1145_cov_5.758079 | 1181 | 0.0 | |  | | | | | |
|  | Cytochrome c oxidase subunit vaa | | NODE_35692_length_709_cov_365.513397 | 745 | 8.8E-92 | |  | | | | | |
|  | Paired box gene 2a | | NODE_35827_length_1055_cov_7.461611 | 1091 | 0.0 | |  | | | | | |
|  | Zinc finger protein 830 | | NODE_35842_length_379_cov_33.269131 | 415 | 4.6E-34 | |  | | | | | |
|  | Wingless-type mmtv integration site member 5b | | NODE_36176_length_954_cov_5.278826 | 990 | 1.9E-17 | |  | | | | | |
|  | irx3a protein | | NODE_36779_length_347_cov_6.043228 | 383 | 1.7E-67 | |  | | | | | |
|  | Vang-like protein 2 | | NODE_37715_length_520_cov_6.246154 | 556 | 1.1E-23 | |  | | | | | |
|  | af391125_1 runx1 transcription factor | | NODE_37954_length_926_cov_9.173866 | 962 | 2.3E-122 | |  | | | | | |
|  | Basic transcription factor 3 | | NODE_37968_length_260_cov_1017.130798 | 296 | 9.5E-31 | |  | | | | | |
|  | Zgc:136689 | | NODE_38154_length_684_cov_5.694445 | 720 | 1.2E-139 | |  | | | | | |
|  | Fork head domain protein fkd4 | | NODE_38361_length_921_cov_6.155266 | 957 | 8.5E-136 | |  | | | | | |
|  | Mitogen activated protein kinase 9 | | NODE_38472_length_533_cov_7.711070 | 569 | 1.4E-128 | |  | | | | | |
|  | Ribosomal protein l11 | | NODE_38589_length_199_cov_2367.738770 | 235 | 1.2E-45 | |  | | | | | |
|  | Vacuolar atp synthase 16 kda proteolipid subunit | | NODE_38775_length_335_cov_338.865662 | 371 | 3.8E-44 | |  | | | | | |
|  | Low density lipoprotein receptor-related protein 6 | | NODE_49740_length_569_cov_5.889279 | 605 | 8.1E-131 | |  | | | | | |
|  | T-box transcription factor tbx2 | | NODE_39321_length_1880_cov_9.192021 | 1916 | 1.3E-169 | |  | | | | | |
|  | myh9 protein | | NODE_39542_length_564_cov_109.297874 | 600 | 1.9E-83 | |  | | | | | |
|  | Zgc:162969 protein | | NODE_40316_length_1392_cov_107.057472 | 1428 | 0.0 | |  | | | | | |
|  | Fibronectin 3 | | NODE_40332_length_558_cov_243.677414 | 594 | 9.9E-105 | |  | | | | | |
|  | Ribosomal protein l6 | | NODE_40390_length_151_cov_2020.033081 | 187 | 5.5E-22 | |  | | | | | |
|  | 60s ribosomal protein l6 | | NODE_40392_length_151_cov_4111.867676 | 187 | 2.1E-33 | |  | | | | | |
|  | 60s ribosomal protein l35a | | NODE_40407_length_151_cov_2441.536377 | 187 | 2.1E-31 | |  | | | | | |
|  | Ribosomal protein s29 | | NODE_41042_length_589_cov_769.572144 | 625 | 1.6E-32 | |  | | | | | |
|  | Zgc:65879 | | NODE_41894_length_1176_cov_14.411565 | 1212 | 0.0 | |  | | | | | |
|  | Cytochrome c oxidase subunit mitochondrial precursor | | NODE_42205_length_663_cov_190.684769 | 699 | 8.8E-92 | |  | | | | | |
|  | hes1 protein | | NODE_42831_length_1334_cov_17.198650 | 1370 | 8.9E-177 | |  | | | | | |
|  | a chain ATPase | | NODE_42865_length_303_cov_41.871288 | 339 | 4.2E-61 | |  | | | | | |
|  | cbp p300-interacting with glu asp-rich carboxy-terminal 2 | | NODE_43303_length_879_cov_73.076225 | 915 | 1.2E-133 | |  | | | | | |
|  | upf1 regulator of nonsense transcripts homolog | | NODE_43348_length_2649_cov_78.436394 | 2685 | 0.0 | |  | | | | | |
|  | cdx4 protein | | NODE_43424_length_1551_cov_14.032882 | 1587 | 8.4E-162 | |  | | | | | |
|  | af219949_1 forkhead transcription factor | | NODE_43941_length_313_cov_6.083067 | 349 | 1.9E-7 | |  | | | | | |
|  | tec protein tyrosine kinase | | NODE_44104_length_514_cov_32.959145 | 550 | 1.3E-99 | |  | | | | | |
|  | lmo2 protein | | NODE_44457_length_1306_cov_19.149311 | 1342 | 1.9E-112 | |  | | | | | |
|  | Collagen type alpha 1 | | NODE_44612_length_528_cov_5.051136 | 564 | 6.3E-129 | |  | | | | | |
|  | Hairy and enhancer of split 6 | | NODE_44661_length_849_cov_12.690224 | 885 | 3.9E-105 | |  | | | | | |
|  | Alanine--glyoxylate aminotransferase 2-like 1 | | NODE_44956_length_1714_cov_17.865227 | 1750 | 0.0 | |  | | | | | |
|  | Dual specificity phosphatase 4 | | NODE_45061_length_1592_cov_17.722990 | 1628 | 0.0 | |  | | | | | |
|  | Transcriptional intermediary factor 1 gamma | | NODE_45127_length_496_cov_30.891129 | 532 | 1.4E-15 | |  | | | | | |
|  | mgc81374 protein | | NODE_45411_length_446_cov_23.946188 | 482 | 1.0E-31 | |  | | | | | |
|  | mps one binder kinase activator-like 1b | | NODE_45438_length_1243_cov_72.197105 | 1279 | 1.0E-152 | |  | | | | | |
|  | rpl6 protein | | NODE_45710_length_107_cov_3085.663574 | 143 | 3.0E-17 | |  | | | | | |
|  | ell associated factor 1 | | NODE_45736_length_1623_cov_10.607517 | 1659 | 3.6E-135 | |  | | | | | |
|  | Heavy polypeptide non-muscle | | NODE_45743_length_440_cov_13.568182 | 476 | 2.6E-52 | |  | | | | | |
|  | Erythroid-specific mitochondrial mitoferrin | | NODE_45882_length_359_cov_31.860723 | 395 | 2.1E-85 | |  | | | | | |
|  | Nuclear autoantigenic sperm protein (histone-binding) | | NODE_46114_length_556_cov_604.248230 | 592 | 2.0E-76 | |  | | | | | |
|  | Chromodomain helicase DNA binding protein 8 | | NODE_46998_length_995_cov_18.078392 | 1031 | 3.3E-167 | |  | | | | | |
|  | wnt-11 protein | | NODE_48111_length_1005_cov_5.587065 | 1041 | 7.6E-166 | |  | | | | | |
|  | Membrane palmitoylated 5a (maguk p55 subfamily member 5a) | | NODE_48213_length_3619_cov_16.488256 | 3655 | 0.0 | |  | | | | | |
|  | b chain crystal structure of a smad4-ski complex | | NODE_48232_length_590_cov_6.450848 | 626 | 3.0E-27 | |  | | | | | |
|  | Amyloid beta a4 precursor protein-binding family a member 1 | | NODE_48808_length_1261_cov_12.835052 | 1297 | 6.8E-166 | |  | | | | | |
|  | Receptor tyrosine kinase flk-1 vegfr-2 | | NODE_49288_length_369_cov_53.680218 | 405 | 2.7E-23 | |  | | | | | |
|  | sufu protein | | NODE_50788_length_1130_cov_28.018583 | 1166 | 0.0 | |  | | | | | |
|  | Homeobox iro protein | | NODE_52400_length_341_cov_6.187683 | 377 | 7.5E-48 | |  | | | | | |
|  | eya2 protein | | NODE_53160_length_248_cov_5.705645 | 284 | 6.0E-55 | |  | | | | | |
|  | Enhancer trap locus 4 | | NODE_53865_length_403_cov_6.657568 | 439 | 3.5E-64 | |  | | | | | |
|  | t-box 3b | | NODE_54139_length_130_cov_6.092308 | 166 | 8.1E-25 | |  | | | | | |
|  | Zgc:171551 protein | | NODE_56075_length_98_cov_11.591837 | 134 | 1.0E-14 | |  | | | | | |
|  | 60s ribosomal protein l24 | | NODE_56533_length_70_cov_1916.914307 | 106 | 5.5E-13 | |  | | | | | |
|  | cyclin-dependent kinase 5 | | NODE_56534_length_627_cov_8.665071 | 663 | 1.2E-122 | |  | | | | | |
|  | Deleted in malignant brain tumors 1 | | NODE_57126_length_268_cov_12.302238 | 304 | 9.3E-27 | |  | | | | | |
|  | Myeloid ecotropic viral integration 1 | | NODE_57586_length_2556_cov_7.250391 | 2592 | 0.0 | |  | | | | | |
|  | MDS1 and EVI1 complex locus protein EVI1 | | NODE_70554_length_472_cov_5.050848 | 508 | 1.9E-114 | |  | | | | | |
|  | Oct1 transcription factor | | NODE_58237_length_677_cov_12.785820 | 713 | 3.7E-134 | |  | | | | | |
|  | GATA-binding protein 2a | | NODE_58492_length_485_cov_6.274227 | 521 | 4.6E-59 | |  | | | | | |
|  | af302936_1 transcription factor sox18 | | NODE_58645_length_808_cov_5.170792 | 844 | 1.4E-121 | |  | | | | | |
|  | Homeobox protein dlx4b | | NODE_59345_length_181_cov_5.845304 | 217 | 3.7E-28 | |  | | | | | |
|  | Eukaryotic translation initiation factor subunit a | | NODE_60376_length_1195_cov_162.039337 | 1231 | 0.0 | |  | | | | | |
|  | Hypothetical microtubule-associated protein 1b | | NODE_69440_length_222_cov_5.391892 | 258 | 4.0E-38 | |  | | | | | |
|  | Translocator protein | | NODE_60732_length_298_cov_85.023491 | 334 | 8.7E-15 | |  | | | | | |
|  | Phosphotidylinositol phosphatase ptprq | | NODE_60961_length_311_cov_5.443730 | 347 | 2.4E-63 | |  | | | | | |
|  | Lamin b receptor | | NODE_61895_length_2187_cov_56.240971 | 2223 | 0.0 | |  | | | | | |
|  | af191577_1 zinc finger transcription factor gata4 | | NODE_62837_length_619_cov_5.126009 | 655 | 2.8E-39 | |  | | | | | |
|  | ptrf protein | | NODE_62878_length_363_cov_41.082645 | 399 | 6.3E-13 | |  | | | | | |
|  | aof2 protein | | NODE_62939_length_501_cov_30.594810 | 537 | 4.8E-20 | |  | | | | | |
|  | lim-domain binding factor 3a | | NODE_63533_length_446_cov_29.031389 | 482 | 4.4E-67 | |  | | | | | |
|  | Zinc finger and btb domain-containing protein 16-a | | NODE_65284_length_300_cov_21.639999 | 336 | 3.1E-70 | |  | | | | | |
|  | Adducin 1 isoform cra_a | | NODE_65475_length_1456_cov_23.548763 | 1492 | 0.0 | |  | | | | | |
|  | GATA zinc finger domain containing 2a | | NODE_65572_length_315_cov_55.031746 | 351 | 5.7E-66 | |  | | | | | |
|  | pdz and lim domain 7 | | NODE_65979_length_2872_cov_31.269150 | 2908 | 0.0 | |  | | | | | |
|  | abl interactor 1 isoform 7 | | NODE_66741_length_671_cov_22.931446 | 707 | 1.3E-139 | |  | | | | | |
|  | Zgc:136731 protein | | NODE_66833_length_1007_cov_5.658391 | 1043 | 2.5E-111 | |  | | | | | |
|  | Insulin-like growth factor binding protein 3 | | NODE_68299_length_541_cov_6.092422 | 577 | 1.1E-37 | |  | | | | | |
|  | Novel protein draculin | | NODE_70664_length_165_cov_7.066667 | 201 | 1.2E-22 | |  | | | | | |
|  | Forkhead box i3b | | NODE_71062_length_352_cov_8.863636 | 388 | 6.0E-71 | |  | | | | | |
|  | af457191_1 transcription factor ap2 alpha 1 | | NODE_71768_length_1093_cov_10.100640 | 1129 | 2.9E-177 | |  | | | | | |
|  | Na+ K+ atpase alpha1a1 subunit | | NODE_72843_length_131_cov_555.725220 | 167 | 1.2E-25 | |  | | | | | |
|  | Elongation factor RNA polymerase ii | | NODE_76868_length_538_cov_26.518587 | 574 | 1.1E-108 | |  | | | | | |
|  | Myosin regulatory light chain interacting protein b | | NODE_77430_length_489_cov_7.787321 | 525 | 2.8E-95 | |  | | | | | |
|  | ADP-ribosylation factor-related protein 1 | | NODE_78247_length_866_cov_31.633949 | 902 | 1.8E-136 | |  | | | | | |
|  | Vascular endothelial growth factor aa | | NODE_79814_length_241_cov_7.419087 | 277 | 1.1E-22 | |  | | | | | |
|  | Protein o-fucosyltransferase 1 | | NODE_84522_length_678_cov_21.502951 | 714 | 4.3E-153 | |  | | | | | |
|  | c20orf45 homolog | | NODE_86328_length_252_cov_97.317459 | 288 | 2.1E-41 | |  | | | | | |
|  | N-acetyltransferase nat13 | | NODE_86910_length_803_cov_31.894148 | 839 | 8.1E-117 | |  | | | | | |
|  | nrf2-associated protein keap1b | | NODE_87674_length_181_cov_10.685082 | 217 | 8.7E-41 | |  | | | | | |
|  | Heavy polypeptide fast muscle specific | | NODE_88339_length_222_cov_7.585586 | 258 | 8.1E-25 | |  | | | | | |
|  | DNA (cytosine-5-)-methyltransferase 8 | | NODE_89029_length_1422_cov_7.310127 | 1458 | 0.0 | |  | | | | | |
|  | Amyloid beta precursor protein family member isoform cra_b | | NODE_89661_length_1820_cov_7.029670 | 1856 | 0.0 | |  | | | | | |
|  | Stem cell antigen 2 | | NODE_96834_length_250_cov_9.564000 | 286 | 6.2E-19 | |  | | | | | |
|  | rho GTPase-activating protein 7 | | NODE_106287_length_1965_cov_18.552671 | 2001 | 0.0 | |  | | | | | |
|  | Neogenin isoform 3 | | NODE_106894_length_238_cov_7.113445 | 274 | 2.5E-45 | |  | | | | | |
|  | af441285_1 nr13 | | NODE_111324_length_245_cov_248.261230 | 281 | 1.0E-56 | |  | | | | | |
|  | Kinesin family member 23 | | NODE_112255_length_992_cov_52.459679 | 1028 | 2.3E-94 | |  | | | | | |
|  | vang-like 2 | | NODE_141347_length_86_cov_7.779070 | 122 | 2.2E-17 | |  | | | | | |
|  | Paxillin | | NODE_152977_length_706_cov_45.232296 | 742 | 0.0 | |  | | | | | |
