# Supplementary material for: Construction, De-Novo Assembly and Analysis of Transcriptome for Identification of Reproduction-Related Genes and Pathways from Rohu, Labeo rohita (Hamilton)
Source: PLoS One. 2015 Jul 6;10(7):e0132450. doi: 10.1371/journal.pone.0132450 (PMC4509579; doi:10.1371/journal.pone.0132450)
Supplement: S3 Table — (DOCX) [file pone.0132450.s006.docx]

| S3 Table. Comparison of repeat types identified in *L. rohita* with corresponding gene sequences of *Cyprinus carpio* and *Danio rario*. | | | | | | |
| --- | --- | --- | --- | --- | --- | --- |
| **Sl No.** | **Genes Identified from *Labeo rohita*** | **Repeat Type in rohu** | ***Cyprinus carpio*** | **Repeat Type in cyprinus** | ***Danio rario*** | **Repeat Type in Danio** |
| **Embryonic development related Proteins** | | | | | | |
|  | brahma protein 1 | (AAG)5 | No Match | - | XM_009302072.1 | (GGA)5AGA)4AAG)5GAG)4AGA)4GAG)5TGA)4 |
|  | myst histone acetyltransferase (monocytic leukemia) 3 | (TCA)5(TCC)4 | No Match | - | NM_001123312.4 | (AG)8GAG)4GGA)5GTG)4GGT)4GAG)4GAT)4CAA)13GAT)5CAA)4TAT)5 |
|  | Tripartite motif protein trim33 | (TGC)5(CCG)4 | No Match | - | NM_001002871.2 | (GGC)4TCC)4(CCA)4CAA)4CAA)8CAA)4TAT)7 |
|  | N-myristoyltransferase 1 | (AAG)4 | No Match | - | NM_001020480.1 |  |
|  | Suppressor of ty 6 homolog | (ATC)4 | No Match | - | NM_145118.1 | (TGA)4GAT)4GAT)4AGA)5AGG)5 |
|  | sp3 transcription isoform cra_d | (AT)7(GTGC)3 | No Match | - | XM_005165801.2 | TA)6AT)5AT)5AC)10 |
|  | smg5 homolog nonsense mediated mrna decay factor protein | (GA)6(GAT)4 | No Match | - | NM_001024923.1 | CT)6GAG)9TCA)6 |
|  | Mitogen-activated protein kinase 1 | (AC)7 | AB006038.1 | - | AB030902.1 | - |
|  | Zgc:153035 protein ( hepatic leukemia factor) | (TGG)4 | No Match | - | NM_001077334.2 | - |
|  | E1A binding protein p300 | (GCA)4(CAG)4(GCA)7(GCA)4(CAG)7(GCA)5(CAA)4(CAG)5(TAT)7 | No Match | - | XM_009306693.1 | - |
|  | smg-5 nonsense mediated mrna decay factor | (CAA)_4_(CCTCT)_3_ | No Match | - | NM_001045083.2 | - |
|  | Tubby-like protein 3 | (GT)10 | No Match | - | XM_689489.6 | - |
|  | Chromodomain-helicase-DNA-binding protein 7 | (TCA)4 | No Match | - | XM_692864.6 | - |
|  | Heavy chain non-muscle | (AAAAT)3 | AB231800.1 | - | XM_005165695.2 | - |
|  | Mediator of RNA polymerase ii transcription subunit 12 | (CTG)5(CTG)4(CTG)4(CTG)4(TTG)5(TGC)4 | No Match | - | XM_009291038.1 | - |
|  | Leucine-zipper protein | (TTCT)3(AACAA)3 | No Match | - | No Match | - |
| **Hormone and Receptor binding related proteins** | | | | | | |
|  | Docking protein isoform cra_a | (CCA)_7_ | No Match | - | XM_002664489.3 | - |
|  | Hepatoma-derived growth related protein 3 | (TG)_7_ | No Match | - | NM_001012372.1 | - |
|  | Ephrin b2 | (TCA)_6_ | No Match | - | NM_131023.1 | - |
|  | Neuregulin 1 type i isoform | (TTAT)_4_ | No Match |  | FJ593487.1 |  |
| **Receptor activity related proteins** | | | | | | |
|  | T-cell receptor beta chain ana | (AC)_7_ | No Match | - | No Match | - |
|  | Protein tyrosine non-receptor type 2 | (GCA)_4_ | No Match | - | NM_200466.1 | - |
|  | Gravin | (TC)_6_ | No Match | - | EF539208.1 | - |
|  | af177465_1 estrogen receptor beta2 | (TC)_6_ | AB334723.1 | - | NM_174862.3 | - |
|  | Growth factor receptor-bound protein 10 | (AC)_6_(TAA)_4_ | No Match | - | NM_001004287.1 | - |
| **Reproduction related proteins** | | | | | | |
|  | b-cell leukemia lymphoma 6 | (ATA)_5_ | No Match | - | [NP_957028.1](http://www.ncbi.nlm.nih.gov/protein/41152254?report=genbank&log$=prottop&blast_rank=2&RID=JU6ETRET013) | - |
|  | Diaphanous homolog 2 | (AACA)_3_ | No Match | - | XM_009291232.1 | - |
|  | HIV-1 REV binding protein | (CAG)4(GCA)_6_ | No Match | - | No Match | - |
|  | TATA box binding protein | (TGCTGT)_3_ | No Match | - | NM_200096.1 | - |
